# The Genome of *Spraguea lophii* and the Basis of Host-Microsporidian Interactions

**DOI:** 10.1371/journal.pgen.1003676

**Published:** 2013-08-22

**Authors:** Scott E. Campbell, Tom A. Williams, Asim Yousuf, Darren M. Soanes, Konrad H. Paszkiewicz, Bryony A. P. Williams

**Affiliations:** 1 Biosciences, College of Life and Environmental Sciences, University of Exeter, Devon, United Kingdom; 2 Institute for Cell and Molecular Biosciences, University of Newcastle, Newcastle upon Tyne, Tyne and Wear, United Kingdom; Duke University Medical Center, United States of America

## Abstract

Microsporidia are obligate intracellular parasites with the smallest known eukaryotic genomes. Although they are increasingly recognized as economically and medically important parasites, the molecular basis of microsporidian pathogenicity is almost completely unknown and no genetic manipulation system is currently available. The fish-infecting microsporidian *Spraguea lophii* shows one of the most striking host cell manipulations known for these parasites, converting host nervous tissue into swollen spore factories known as xenomas. In order to investigate the basis of these interactions between microsporidian and host, we sequenced and analyzed the *S. lophii* genome. Although, like other microsporidia, *S. lophii* has lost many of the protein families typical of model eukaryotes, we identified a number of gene family expansions including a family of leucine-rich repeat proteins that may represent pathogenicity factors. Building on our comparative genomic analyses, we exploited the large numbers of spores that can be obtained from xenomas to identify potential effector proteins experimentally. We used complex-mix proteomics to identify proteins released by the parasite upon germination, resulting in the first experimental isolation of putative secreted effector proteins in a microsporidian. Many of these proteins are not related to characterized pathogenicity factors or indeed any other sequences from outside the Microsporidia. However, two of the secreted proteins are members of a family of RICIN B-lectin-like proteins broadly conserved across the phylum. These proteins form syntenic clusters arising from tandem duplications in several microsporidian genomes and may represent a novel family of conserved effector proteins. These computational and experimental analyses establish *S. lophii* as an attractive model system for understanding the evolution of host-parasite interactions in microsporidia and suggest an important role for lineage-specific innovations and fast evolving proteins in the evolution of the parasitic microsporidian lifecycle.

## Author Summary

Microsporidia are unusual intracellular parasites that infect a broad range of animal cells. In comparison to their fungal relatives, microsporidian genomes have shrunk during evolution, encoding as few as 2000 proteins. This minimal molecular repertoire makes them a reduced model system for understanding host-parasite interactions. A number of microsporidian genomes have now been sequenced, but the lack of a system for genetic manipulation makes it difficult to translate these data into a better understanding of microsporidian biology. Here we present a deep sequencing project of *Spraguea lophii*, a fish-infecting microsporidian that is abundantly available from environmental samples. We use our sequence data combined with germination protocols and complex-mix proteomics to identify proteins released by the cell at the earliest stage of germination, representing potential pathogenicity factors. We profile the RNA expression pattern of germinating cells and identify a set of highly transcribed hypothetical genes. Our study provides new insight into the importance of uncharacterized, lineage-specific and/or fast evolving proteins in microsporidia and provides new leads for the investigation of virulence factors in these enigmatic parasites.

## Introduction

Microsporidia are a diverse phylum of obligate intracellular parasites related to fungi. Over 1300 species have been described in approximately 160 genera and, as is the case for other microbial eukaryotes, a vast undescribed diversity is thought to exist in the environment [1]. Microsporidia are important pathogens of a broad range of animal groups: they can infect immunocompromized humans, such as those with HIV/AIDS, and are major pathogens of fish and invertebrates, representing a significant threat to sericulture [2] and fisheries [3]. Their unusual lifecycle has also attracted attention, particularly the unique mechanism by which microsporidia gain entrance to host cells. Outside the host cell, microsporidia exist as a resistant spore containing a coiled polar tube. Upon coming into contact with a host cell, or appropriate stimulus, the spore rapidly everts this tube, penetrating the host cell membrane and delivering the spore contents to the host cytoplasm, where proliferation and the next round of spore production occurs [4].

In addition to their importance as parasites of animals, Microsporidia have attracted much attention as eukaryotic model systems for reductive genome evolution. The 2.9 Mb genome of the microsporidian *Encephalitozoon cuniculi* was one of the first eukaryotic genomes to be sequenced [5]. Analysis of the *E. cuniculi* genome revealed a highly reduced and streamlined genome which had lost or simplified many biochemical pathways, had truncated genes, shortened intergenic spaces and had almost entirely lost introns and repetitive DNA [5]; its close relative *Encephalitozoon intestinalis* has an even smaller genome, at 2.3 Mb [6]. Interestingly, while microsporidian genomes are consistently smaller than those of their opisthokont relatives, there is a ten-fold difference in genome size within the phylum, with some genomes as large as 24 Mb [7]. To date, it has proven difficult to relate this genomic variation to differences in parasite biology or host preference. This is because all microsporidia sequenced so far share a broadly conserved core proteome, with differences in genome size due largely to changes in gene density, transposon content, and expansions of uncharacterized, lineage-specific or fast evolving protein families [8]–[10]. As the main differences in coding capacity among sequenced microsporidian lineages, it seems reasonable to hypothesize that these lineage-specific or fast evolving proteins play a role in mediating host-parasite interactions. However, because they lack any detectable similarity to genes from model eukaryotes, the functions of these proteins are difficult to predict using bioinformatics. Combined with the current lack of a system for genetic manipulation in these parasites, this makes understanding the basis of host-microsporidian interactions extremely challenging. Beyond identification of proteins of the spore wall and polar tube, very little known about molecular basis of spore germination and eversion of the polar tube, or the exact details of how the microsporidian sporoplasm is transferred into the host cell. Even though microsporidia can have drastic effects on the organization of the host cell, we know little of the virulence factors and effector proteins that bring about these changes or how they are delivered into the host cell environment either directly or via the parasitophorous vacuole in human infective species such as *E. cuniculi* and *E. intestinalis*.

**Figure 1:**
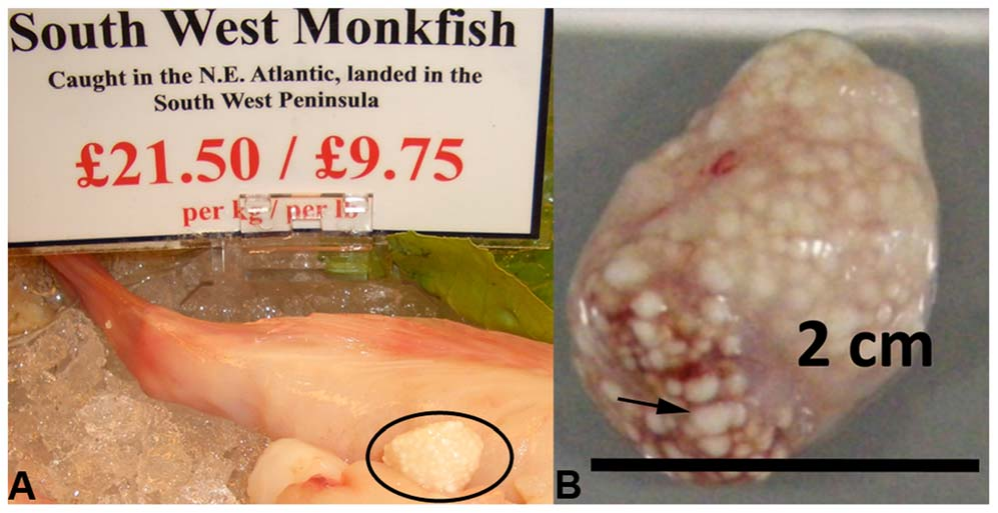
Microsporidian xenomas *in situ*. *S. lophii* grows and replicates inside large cysts visible to the naked eye; these cysts are comprised of swollen, spore-filled fish cells called xenomas. **A**. Cyst of *S. lophii* xenomas *in situ* in commercially available monkfish (circled). **B**. Close up of a cyst of xenomas. Arrow points to a single enlarged fish cell (xenoma).

*Spraguea lophii* is a microsporidian that infects the monkfish *Lophius piscatorius* and *Lophius budegassa*, inhabiting both the North Atlantic and Mediterranean regions [11]. Compared to those microsporidia that have been sequenced so far, *S. lophii* is an attractive model for identifying microsporidian effector proteins and investigating host-parasite interactions, despite the current lack of an *in vitro* culture system [12]. Infection with *S. lophii* results in the formation of xenomas, large clusters of spore-filled cells in the vagal nerves of the fish that can be several centimeters in diameter and contain spores in various stages of development (Figure 1). In the monkfish, no fitness effects are known to be associated with *Spraguea* infection, and the prevalence rate can be as high as 83% [13], [14]. However, xenoma formation in salmon infected with related microsporidia commonly localizes to the gills and is a considerable threat in aquaculture [15]. *S. lophii* spores are easily purified from xenomas in large quantities, providing an opportunity to study germination and to perform experiments that are difficult or impossible in other microsporidia due to the difficulty of obtaining sufficient amounts of parasite material. In addition, *S. lophii* is the first microsporidian parasite of fish to be sequenced, with the potential to provide new insights into host-parasite interactions in these economically important vertebrates. Our aim here is to provide a genomic resource that will facilitate future work on this promising model microsporidian.

*S. lophii* was the first microsporidian to be explored with genome-scale sequencing, and 120 Kb of the genome has previously been published [16]. Here we present 4.98 Mb of unique sequence from the *S. lophii* genome as determined by Illumina sequencing, representing 70–80% of the complete genome (estimated at 6.2–7.3 Mb [17]–[19]) and, based on our analyses, the great majority of the coding DNA. We investigated the evolution of the *S. lophii* proteome, using OrthoMCL [20] to identify proteins that are unique to *S. lophii* and may therefore be associated with the unique xenoma formation seen in microsporidian infections of fish. To explore the utility of *S. lophii* as a model for understanding core features of microsporidian biology, we combined complex mix proteomics with TruSeq transcriptomics to characterize the proteins expressed and secreted during spore germination, a key time point in the lifecycle of this intracellular parasite. Our results highlighted the importance of microsporidia-specific and fast evolving proteins in germination and host interaction.

## Results

### Genome architecture of *Spraguea lophii*

The sequence data are summarized in Table 1. Our sequencing resulted in 1392 contigs over 500 bp in length to give a total of 4982 Kb (This Whole Genome Shotgun project has been deposited at DDBJ/EMBL/GenBank under the accession ATCN00000000. The version described in this paper is version ATCN01000000). These contigs had a maximum length of 46788 bp and an N50 length of 5923. The average coverage across these contigs is 70× (Mode = 29×, Median = 59×). The overall GC content is low at 23.4%, rising slightly to 25.7% in protein coding regions. The karyotype has been investigated both by pulse field electrophoresis and by using an ultrathin multiwire proportional chamber-based detector [17]–[19]. This predicts a variable karyotype between isolates from different geographic regions, but the consensus is that there are 15 chromosomes, of which 10–13 are unique, and the overall genome size is estimated at 6.2 to 7.3 Mb. In our assembly we identified 2,573 predicted open reading frames or fragments of, which made up 52% of our assembly. This gives it an intermediate coding density amongst microsporidia, which is consistent with an emerging pattern for microsporidian genomes where coding density decreases with increasing genome size. For example, *Trachipleistophora hominis* has 34% coding DNA and an estimated genome size of 8.5–11.6 Mb, while 86% of the 2.9 Mb *E. cuniculi* genome is made up of coding DNA [5], [8]. Our assembly covers 70–80% of the *S. lophii* genome based on size estimates, but is likely enriched for coding regions. To evaluate the completeness of our assembly, we searched the *S. lophii* genome for the presence of genes involved in a range of core metabolic pathways as described previously [8], [21] (Table S1). Our assembly encodes most major metabolic pathways in full, including glycolysis and trehalose metabolism as well as a full complement of transfer RNAs (tRNAs) and protein kinases (Table S1). We identified only a few absences, many of which are also absent in related microsporidia such as *T. hominis*, lending support to our coverage of the protein-coding component of the genome. As an independent check on the completeness of our assembly, we compared the transcripts from our *de novo* transcriptome assembly (see below) to the genes predicted on the genome. Based on BLAST similarity to genes from other microsporidia, there were only 20 additional *S. lophii* genes in the transcriptome that did not map to the genome assembly, 5 of which represent transcripts from LTR retrotransposons. Taken together, these analyses suggest that our assembly represents a largely complete sampling of the coding component of the *S. lophii* genome.

**Table 1:** Summary of sequence data.

Total number of analysed contigs (over 500 bp)	1392
Average length	3578
Total amount of assembled sequence data in contigs >500 bp	4982267
Total number with open reading frames	1116
Total number of annotated proteins or truncated proteins	2543
Total number of tRNAs	53

### Loss and retention of metabolic pathways in *Spraguea lophii*

**Figure 2:**
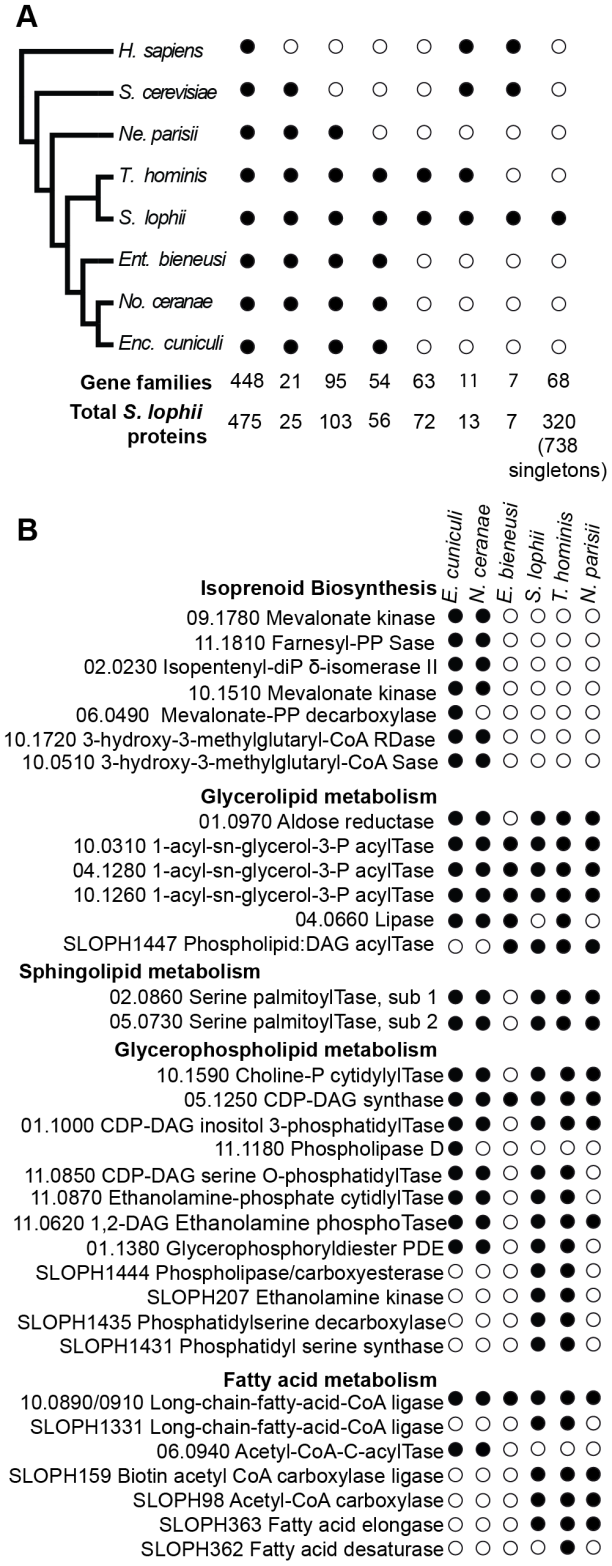
Comparison of the gene content of *S. lophii* to published microsporidian genomes. **A**. Phylogenetic distribution of annotated *S. lophii* proteins. Gene families (OrthoMCL clusters) were mapped on to a multigene phylogeny of microsporidia and their opisthokont relatives (see also Figure 7A.). **B**. Graphical illustration of the variation in fatty acid synthesis enzymes encoded by six different microsporidian genomes. Although the *S. lophii* genome is larger than that of *E. cuniculi*, it has lost some components of the pathway that are conserved in the smaller genome, such as isoprenoid biosynthesis. Reference numbers are given for *E. cuniculi* or *S. lophii* locus ID's.

To investigate the evolution of gene content in *Spraguea*, we mapped the taxonomic distribution of microsporidian gene families onto a cladogram (Figure 2A) (derived from a multiprotein phylogeny - see below). We built gene families using OrthoMCL [20] on a broad sampling of microsporidian genomes, with *Homo sapiens* and *Saccharomyces cerevisiae* as opisthokont outgroups. Our analysis included genomes covering a broad taxonomic spectrum of sequenced microsporidia, including *Nematocida parisii* Ertm1 [22], *T. hominis* [8], *Nosema ceranae* [23], *E. cuniculi* [5], and *Enterocytozoon bieneusi* [24]. The results indicated that 19% of the predicted proteins are shared with all sampled opisthokonts, 1% are specific to sampled fungi, 4% are specific to microsporidia and conserved across the group, 3% are found in clusters of proteins only present in *T. hominis* and *S. lophii*. 42% the 2499 analysed *S. lophii* proteins, do not cluster with proteins from any of the other organisms in our analysis and of these 30% cluster with other *S. lophii* proteins, indicating that they are part of multiprotein families within the *S. lophii* genome. Table S2 gives a complete classification of the *S. lophii* proteome by OrthoMCL analysis.

*E. cuniculi* and other microsporidia have a reduced lipid metabolism repertoire [5], [24]. A key missing step is the initial reaction of fatty acid synthesis, the carboxylation of acetyl-CoA to malonyl-CoA by acetyl-CoA carboxylase. Homologues of both acetyl-CoA carboxylase and the biotin-[acetyl-CoA-carboxylase] ligase that it depends upon are present in the *S. lophii* genome as well as in the *T. hominis* and *N. parisii* genomes, suggesting that they are capable of performing this reaction. However, as in *E. cuniculi*, no fatty acid synthase is evident, though both a fatty acid elongase and desaturase are present (Figure 2B). Interestingly, the presence of these additional components in *S. lophii* was predicted on the basis of comparative liquid chromatography of the lipid composition of *E. cuniculi* and *S. lophii* spores which showed a higher level of docosahexaenoic acid, an unsaturated fatty acid, in *S. lophii* than *E. cuniculi* [25]. In comparison to *E. cuniculi*, the *S. lophii* genome also encodes more enzymes for glycerophospholipid synthesis, allowing for a greater variety of interconversions between different types of phospholipid for membrane integration. In contrast, and despite more complexity in some aspects of fatty acid metabolism, no components of the isoprenoid biosynthesis pathway were present in the *S. lophii* genome, though these are encoded in the *E. cuniculi* and *N. ceranae* genomes [5], [23] (Figure 2B). These are also absent from our transcriptome data, and from the genomes of *T. hominis*, *N. parisii* and *E. bieneusi*. Taken together, these results suggest that several lineages of microsporidia have independently lost the ability to biosynthesize isoprenoids, a capability that is otherwise conserved across the tree of life [24]. This pathway has been shown to be essential in prokaryotes, and whilst some parasitic Apicomplexa have replaced the classical mevalonate biosynthesis pathway with the alternative MEP pathway [26], it too is absent from these microsporidian genomes. These data suggest that some microsporidia scavenge sterols from the environment. This pathway has been lost in microsporidia with diverse hosts: *E. bieneusi* (mammals, insects), *T. hominis* (mammals, insects), *N. parisii* (worms) and *S. lophii* (fish), suggesting that there is nothing specific about the host biochemical environment that is driving the loss of this pathway.

Key components of the RNAi system are encoded by the *S. lophii* genome, including a dicer protein, an argonaute protein and fragments of an RNA dependent RNA polymerase. This is consistent with emerging genomic data for microsporidia with larger genomes such as *N. ceranae* and *T. hominis* that possess transposable elements [8], [23]. This suggests that RNAi was present in the common ancestor of microsporidia but has been secondarily lost in highly reduced genomes such as *E. cuniculi* and *E. bieneusi* [5], [8], [24]. A comparable situation exists in ascomycete fungi, where RNAi has been lost from the compact *S. cerevisiae* genome but is conserved in other budding yeasts [27].

Relatively few proteins with homology to characterized domains are found in *S. lophii* but no other microsporidia. There are proteins annotated on the basis of similarity to PFAM domains such as kinases, phosphatases and acetyltransferases, which are difficult to relate to specific functions within the cell. One notable exception is a glutamate-ammonia ligase domain containing protein, which can catalyze the generation of glutamine from glutamate and ammonia [28]. This protein is nested between genes with homologs in other microsporidian genomes (a DNA binding protein and acetyl-CoA carboxylase) (Figure S1) and in phylogenetic analysis does not fall into a clade with other fungi, but rather with prokaryotes meaning that it is not clear whether this gene was acquired by lateral transfer or by vertical inheritance. Fish excrete their nitrogenous waste products as ammonia across gills, but glutamate-ammonia ligases are expressed in the brains of fish and other vertebrates to protect from fluctuations in ammonia levels [29]. This protein may have a similar role in protection against ammonia stress in the microsporidian: Whilst the fish is alive the microsporidia may be protected by host ammonia defense mechanisms, however, once the fish dies, microbial degradation of the fish can increase ammonia levels [30]. As the spores of *S. lophii* are embedded deeply within the nervous tissue of the monkfish, they may have to be liberated after the death of the fish, and this glutamate-ammonia ligase may allow the spores to survive fluctuations in ammonia levels in the decaying fish tissue.

Despite the relatively close relationship of *T. hominis* to *S. lophii* (Figure 2A), these two species share few genes that are not found in other microsporidian genomes in our comparison, and most of these are uncharacterized, lineage-specific or fast evolving genes with no similarity to genes in other lineages. A handful of genes shared between *T. hominis* and *S. lophii* have a function that can be predicted on the basis of homology to characterized proteins from model organisms. These include an arsenite transporting ATPase, which might act as an efflux pump in the cell membrane and a cold shock domain protein, which could allow the cell to survive at lower than optimal temperatures [31].

### Leucine rich repeat (LRR) proteins

**Figure 3:**
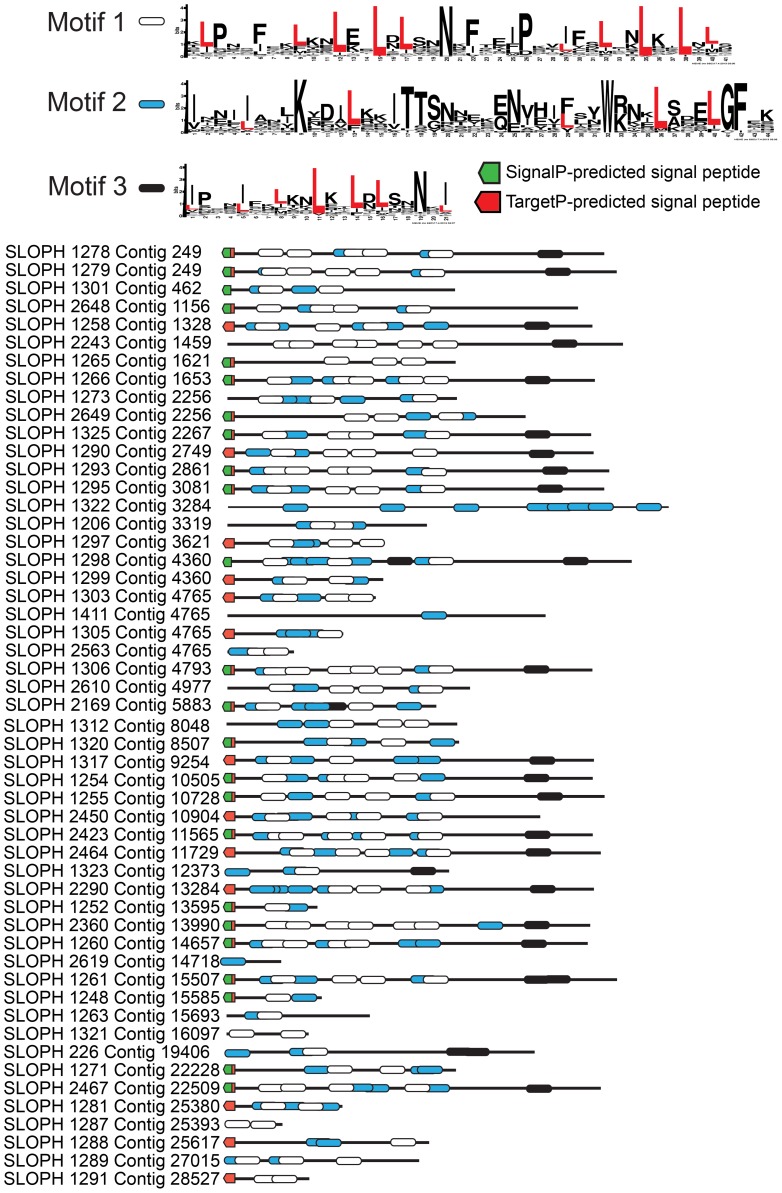
An expanded family of 52 complete leucine-rich repeat proteins in the *S. lophii* genome. This family represents the largest *S. lophii*-specific protein family expansion, and interestingly, many of its members have N-terminal signal peptides, raising the possibility that some may be secreted or targeted to the parasite cell surface for host interactions. Leucine-rich motifs at non-overlapping sites with a p-value lower than 0.0001 were identified using MEME [34] and signal peptides identified using SignalP 4.1 (green) and TargetP (red) [37].

The largest protein family expansion present in *S. lophii* is a family of proteins containing LRRs (Figure 3). Fungal genomes generally encode fewer LRR proteins than their animal relatives [32] though expansion of LRR protein families as pathogenicity factors is known in pathogenic fungi [33]. The genome of *E. cuniculi* encodes just 9 LRR genes in total [5], yet in stark contrast, we have found 97 ORFs encoding fragments of LRR proteins in the *S. lophii* genome, 52 of which appear complete (that is, they have a predicted start and stop codon), and 35 of which appear in our transcriptome data. We used PFAM and MEME [34], [35] to identify common conserved motifs, SignalP 4.1 and TargetP 1.1 to look for presence or absence of a secretion signal, and TMHMM 2.0 [36], [37] to look for evidence of transmembrane domains that could anchor the proteins in the membrane of the parasite as a potential LRR receptor protein. MEME analysis shows the presence of three different leucine-enriched motifs in the proteins (Figure 3). We also found that the majority of the proteins (37/52) have a predicted signal peptide but no transmembrane domain, meaning that they are potentially a family of secreted parasite effector proteins (Figure 3). Leucine rich repeat proteins often mediate protein-protein interactions, particularly through the formation of dimers [38]. One possibility is that, if these microsporidian LRR proteins are secreted into the host, they could potentially interfere with the formation of dimers of host proteins, disturbing the functional dimer-monomer cycle by sequestering them into inactive dimers, a mechanism seen in mammalian cells [39]. Perhaps surprisingly, similar sequences are also found in the distantly related microsporidian parasite of humans, *Vittaforma corneae*, but in no other microsporidian species for which there is an available genome sequence (Table S3). A large family of leucine-rich proteins was recently reported in another microsporidian, *T. hominis*, although the two families are not related and no predicted secretion signals were reported for the *T. hominis* family [8].

Although the *S. lophii* genome encodes 97 LRR-containing ORFs, we have evidence of expression for only 35. Therefore, we cannot exclude the possibility that some family members are never expressed and are pseudogenes. In other eukaryotic parasites such as *Trypanosoma* and *Giardia*, large protein families contain many pseudogenes which provide the genetic variation for the ongoing process of antigenic switching [40], [41]. If microsporidian multigene families interact with the host, switching of expression from gene to gene may allow escape from the fish adaptive immune response over the course of infection [42].

### The transcriptome of germinated *S. lophii* spores

The cDNAs of artificially germinated spores were sequenced by the non strand-specific TruSeq approach using Illumina sequencing. This Transcriptome Shotgun Assembly project has been deposited at DDBJ/EMBL/GenBank under the accession GALE00000000. The version described in this paper is the first version, GALE01000000. We used Trinity RNA-Seq [43] to assemble the spore transcriptome, and RSEM [44] to quantify the relative abundance of transcripts. Our *de novo* transcriptome assembly contained 12,932 unique transcripts, of which 2,896 mapped to the *S. lophii* genome. Although relatively small in number, these transcripts made up 67.7% of the transcriptome by abundance, and likely represent the majority of the *S. lophii* genes in the dataset. 2,514 of these transcripts mapped to existing *S. lophii* genes, so that 1,598 of the predicted 2,539 open reading frames had at least one matching transcript. A small proportion of transcripts (398) mapped to regions of the genome assembly without gene predictions; manual inspection of these cases provided evidence for 30 additional genes that had been missed by the initial annotation process.

The remaining third of transcripts that did not map to the *S. lophii* genome assembly might represent contaminants, unmapped *S. lophii* genes, or artifacts of the assembly process. To distinguish between these possibilities, we searched these transcripts against the NCBI nr database using BLASTX [45]. Many showed high sequence identity to *Pseudomonas* and *Flavobacterium* genes and likely represent contaminants. We did, however, identify 20 additional genes with best BLAST hits among the Microsporidia, particularly *T. hominis* and *Vavraia culicis*; these are most likely *S. lophii* genes from the unassembled portion of the genome (Table S4). Interestingly, this set of 20 genes included one LTR and four non-LTR retrotransposons with similarity to those found on the *T. hominis* genome [8]. While the *T. hominis* retrotransposons are largely fragmented and pseudogenized, these results demonstrate that at least some of their homologues in *S. lophii* remain active.

Overall, our analyses of the *S. lophii* transcriptome provided support for the completeness of our genome assembly and for our gene calling approach, and also provided some new insights into microsporidian biology. Of the 1,986 genes in our genomic data that have complete open reading frames, that is, they have a start and a stop codon, 265 have complete coverage and for these, the RNA transcript was on average 132 base pairs longer than the gene. Five of these show more than one gene in the transcripts. However, given the short read-length of Illumina sequences and the possibility that transcripts of adjacent divergent genes could erroneously assemble, it is not possible to say whether these transcripts are overrunning into downstream genes as seen in other microsporidian species [46], [47].

**Figure 4:**
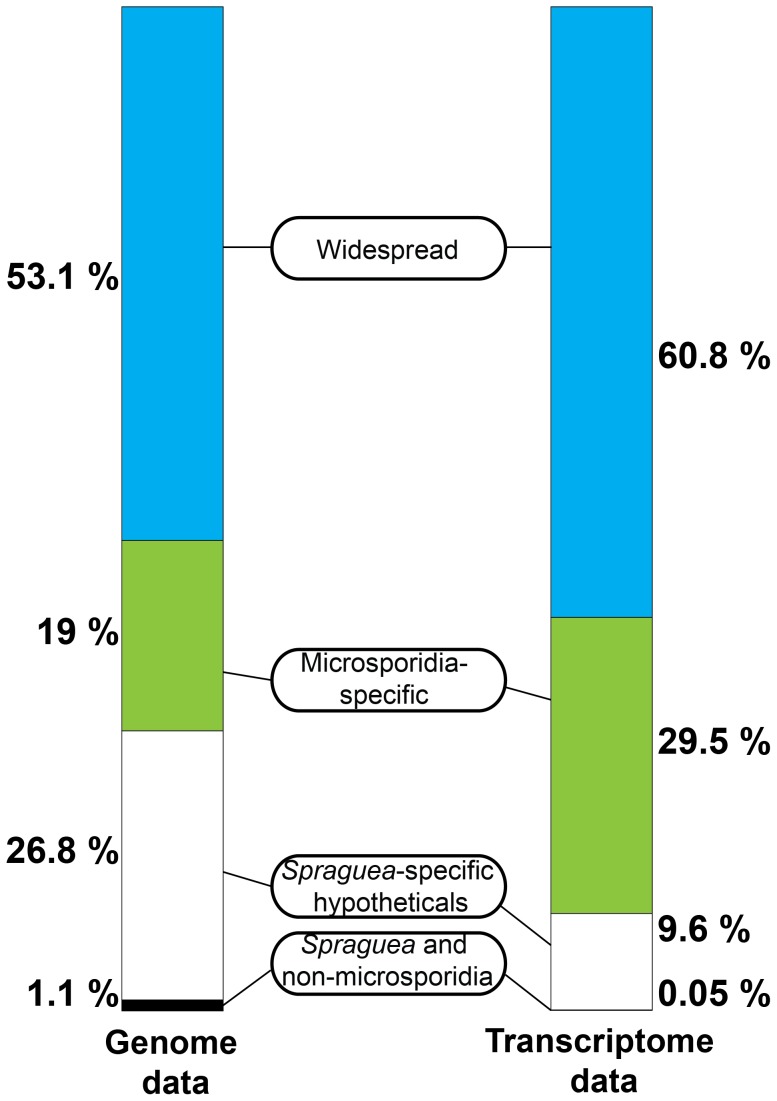
Taxonomic distribution of predicted ORFs in the genome and transcriptome of *Spraguea*. For taxonomic profiling, BLASTP was used to search against the NCBI nr database in January 2013, with a cutoff value of e<1×10^−5^. ORFs were classified into the four illustrated categories according to the results. Microsporidia- and *S. lophii*-specific ORFs and fast-evolving proteins with no similarity to proteins from other lineages make up a substantial proportion of the transcriptome, suggesting an important role for lineage-specific (or fast evolving) genes in the evolution of microsporidia.

Interestingly, although the most abundant transcripts corresponded to 18S ribosomal RNA (Table 2), the most highly expressed protein in *S. lophii* spores is an uncharacterized ORF, with homologues found only in a limited number of other microsporidia. Indeed, while the list of highly transcribed proteins contained many of the expected candidates (ribosomal proteins, ATP-binding proteins, transcription factors, and proteins involved in energy metabolism), these were intermingled with a number of uncharacterized proteins annotated as hypotheticals (see Table 2), strongly suggesting an important role for novel, lineage-specific or very fast evolving proteins in *S. lophii* and microsporidian biology. An interesting observation is that microsporidia-specific proteins conserved in multiple species make up a larger proportion of the transcriptome than *Spraguea*-specific proteins (Figure 4), and a larger proportion of the transcriptomic data than they do of the genomic data. These potentially novel proteins originating in the common ancestor of microsporidia may be good targets for future experimental work on the maintenance of the parasitic lifecycle both in *S. lophii* and other microsporidian species.

**Table 2:** The twenty most abundant protein-coding transcripts in the *S. lophii* transcriptome.

Protein ID	Protein annotation	Abundance as percentage of transcriptome
SLOPH 2267	hypothetical protein SLOPH 2267	3.2715733
SLOPH 2328	ADP-ribosylation factor	3.133362
SLOPH 857	hypothetical protein SLOPH 857	2.7781082
*SLOPH 54	60S acidic ribosomal protein P2	2.127622
SLOPH 997	hypothetical protein SLOPH 997	1.9514969
*SLOPH 2039	ubiquitin/40s ribosomal protein S27a fusion	1.7599442
+SLOPH 655	hypothetical protein SLOPH 655	1.5733076
SLOPH 2438	GTP-binding protein rho1	1.4452069
SLOPH 1855	hypothetical protein SLOPH 1855	1.245524
*SLOPH 1540	RAS GTPase	1.0318709
*SLOPH 2369	14-3-3 protein	0.969516
+SLOPH 431	Histone H3	0.9530647
SLOPH 2171	40S ribosomal protein S28	0.8755324
SLOPH 128	AN1-like Zinc finger protein	0.7700206
SLOPH 1788	hypothetical protein SLOPH 1788	0.7537534
*SLOPH 101	actin	0.7490758
SLOPH 2162	rRNA binding protein	0.644547
SLOPH 1405	nucleoside diphosphate kinase	0.6361997
SLOPH 1378	myosin regulatory light chain	0.6137582
SLOPH 1203	hypothetical protein SLOPH 1203	0.6130135

+ Also found in secreted proteomics data.

* Also found in germinated or non-germinated proteome data.

### Splicing and introns in *S. lophii*

**Figure 5:**
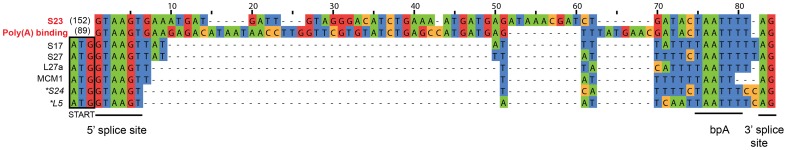
Putative introns in *S. lophii*. The regions corresponding to the conserved microsporidian motifs that interact with the splicing machinery (5′ and 3′ splice sites, branch point bpA) are indicated [49]. The splicing of introns highlighted in red (S23 and poly(A) binding protein) was confirmed at the transcriptome level; transcripts from the other genes retained the intron sequence, suggesting that they are rarely, if ever, spliced in *S. lophii*. The actively spliced introns are located 152 and 89 nucleotides downstream of the translation start site, while all others are immediately adjacent to the start codon (outlined in black). Genes marked with an asterisk could, in principle, be read through to produce a short insertion in the protein sequence, but the others cannot be read through because they contain an in-frame stop codon.

Our *de novo* transcriptome assembly also enabled us to investigate the splicing of putative introns in *S. lophii* protein-coding genes. The number of introns in microsporidian genomes is greatly reduced compared to their opisthokont relatives [48]. *E. cuniculi*, *N. ceranae*, and *T. hominis* encode a relatively small (6–78) number of spliceosomal introns, which are largely confined to ribosomal proteins, while the *Nematocida* genus appears to have lost both introns and the splicing machinery entirely [22]. *S. lophii* does encode conserved components of the splicing machinery, so we searched its coding sequences for introns using a two-step approach. First, we scanned the genome with a consensus microsporidian intron motif built from comparisons of the introns in *E. cuniculi* and *T. hominis* [5], [8], [49]. This search returned hits to 8 genes, 6 of which encode ribosomal proteins (Figure 5); the two non-ribosomal proteins included genes encoding the DNA replication licensing factor Mcm1 and a poly(A) binding protein. We then searched the *S. lophii* transcriptome for transcripts containing deletions relative to the genome assembly, which might also indicate the presence of introns. Surprisingly, this analysis identified only two transcripts from which the predicted intron sequence had been spliced, corresponding to two of the eight genes identified by our motif scan (ribosomal protein S23 and poly(A) binding protein, Figure 5), transcripts for the other six genes still contained the putative intron motif. A comparison of the sequences of the two actively spliced introns with those of the other intron-like sequences revealed three striking differences (Figure 5): the spliced introns are much longer, are out of frame with the coding sequence, and are located further downstream from the 5′ end of the gene (89 and 152 nucleotides 3′ of the start codon, as opposed to directly adjacent to that codon in all other cases). Of the eight putative intron-containing genes we identified in *Spraguea*, five have orthologues in *E. cuniculi* that also contain an intron (S17, L27a, S24, L5 and poly(A) binding protein), and the efficiency with which those introns are spliced parallels our results with the *S. lophii* transcriptome. The introns in *E. cuniculi* S17, L27a, S24 and L5, for which we did not detect splicing in *Spraguea*, are also short [49] and are among the least efficiently spliced genes in *E. cuniculi*, with less than 15% of transcripts experiencing splicing (a figure which drops to 5% for L5) [50]. In contrast, the *E. cuniculi* orthologue of the actively-spliced poly(A) binding protein contains the longest and most frequently spliced intron in *E. cuniculi*, with over 80% of transcripts spliced. Thus, it appears that the properties determining intron splicing efficiency are conserved between these two distantly related microsporidia; it will be interesting to see if they hold more generally for other intron-containing microsporidian genomes.

These observations raise the question of how genes containing intron-like sequences that are rarely, if ever, spliced can be adequately expressed in *Spraguea*. The genes containing these motifs encode some of the most widely conserved and functionally important proteins in cellular life forms, including components of the ribosome and a DNA replication factor. Several of these intron-containing proteins were identified in our whole cell protein analysis of germinated and non-germinated spores. Ribosomal proteins S27, S24 and L5 were present in our germinated sample and S27 and L5 were also present in the proteome of dormant spores (Table S5). For two of the six genes (S24 and L5), inspection of the intron-like sequence suggests a simple explanation: indels in these sequences have caused a frameshift such that the intron can be read through from the upstream ATG without encountering an in-frame stop codon. Thus, a full-length protein containing a short N-terminal insertion could be expressed from these transcripts in the absence of splicing. The other four introns contain in-frame stop codons such that translation from the upstream ATG is not possible unless the intron is spliced. We considered the possibility that translation could instead begin at an alternative start codon downstream of the intron. However, initiation at the next available, in-frame ATG would result in substantial N-terminal deletions (covering 25–37% of the coding sequence) for three of these genes, and in the case of ribosomal protein S17 no suitable alternative start codon is available. Thus, it remains unclear whether these genes can be expressed without splicing, or whether translation depends on a rate of splicing too low to be detected in our assay; it was recently suggested that low rates of transcript degradation might partially ameliorate this problem in *E. cuniculi* [50].

### Proteomics of germination and secretion

Dissecting the interactions between microsporidia and their host requires an understanding of the process of spore germination, in which the spore leaves dormancy and rapidly expels a long polar tube, through which the spore's cellular contents exit the spore and enter the host cell. The specific host cell stimuli inducing microsporidian spore germination are unidentified and likely complex, however several successful methods for artificial spore germination *in-vitro* have been described in a range of species [51]–[53]. At present, both the changes within the spore that trigger germination, and the identity of the secreted effector proteins used by the parasite gain entry into the host cell and control its biology are unknown. Genetic manipulation is a powerful tool for identifying these factors in many pathogens, but is not yet available for any microsporidian. However, *S. lophii* xenomas are densely packed with spores, providing an abundant source of parasite material for proteomic comparisons between the dormant and germinated spore stages.

To identify any proteins present in artificially germinated but not dormant spores, we analyzed whole protein extractions of both lifecycle stages with mass spectrometry of complex protein mixtures. After pooling and filtering 3 biological replicates our analysis showed no consistent variation at the proteomic level between the two lifecycle stages (Table S5). We did find components of many core pathways in germinated and non-germinated spores, such as histones, heat shock protein and ribosomal proteins. We also find glycolytic enzymes in both germinated and non-germinated spores, which is consistent with recent work that glycolytic pathways are specifically active in the spore stage [8], [54]. Two components of the secretory pathway (Sec23 and Sec24) were identified only in germinated spores, which may indicate its activation specifically upon germination, however these were not consistently found in all three replicates and overall we found a surprisingly conserved repertoire of proteins between the two samples. It may be that germination happens too rapidly to allow for translation, with microsporidia pre-packaging the proteins needed for immediate use upon recognition of the germination stimulus and therefore that obvious changes in protein complement may come later in development in meront and sporogonial stages. Alternatively it may be that the samples are dominated by highly expressed housekeeping proteins, and the proteins that vary between the two samples may be present at low levels not easily detectable by complex mix proteomics.

**Figure 6:**
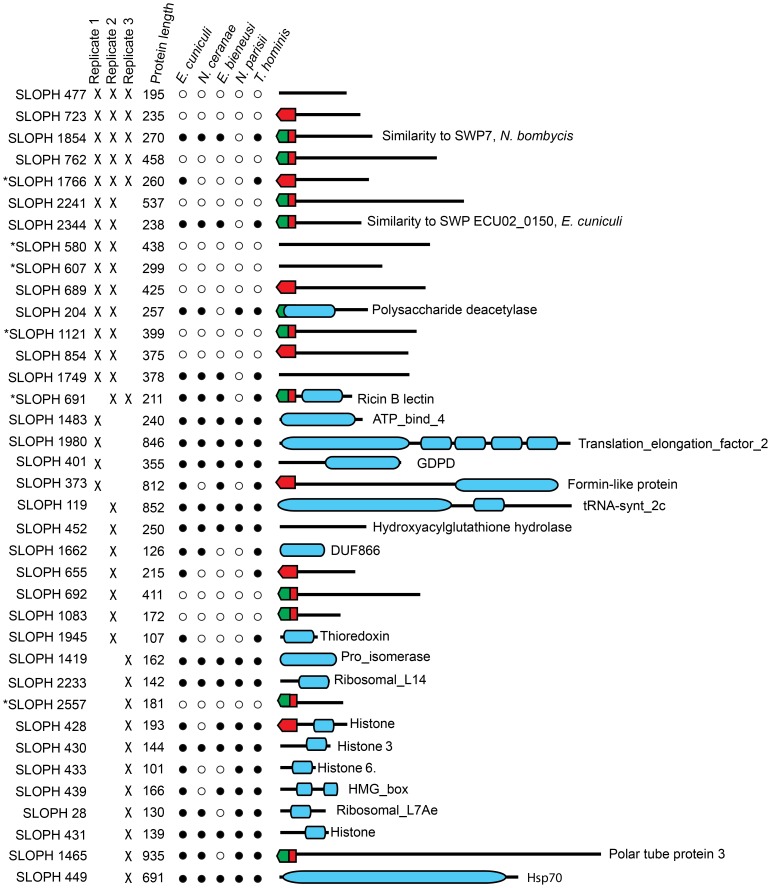
Proteins found in mass spectrometry analysis of medium from germinated *S. lophii* spores. Targeting signals predicted by SignalP (green) or TargetP (red) are indicated. Presence in each replicate of the experiment is indicated with a cross. Stars next to proteins indicate the presence of multiple orthologues of the protein in the *S. lophii* genome. Presence or absence of the protein in other microsporidian genomes is indicated with a full or empty circle respectively. Recognized PFAM domains are indicated by blue bars.

Next, we investigated the complement of proteins present in the extracellular medium after *in-vitro* germination. Here, we consistently retrieved a small subset of proteins that were visualized by SDS-PAGE (Figure S2). Importantly, no proteins were identified from the supernatant of non-germinated *S. lophii* spores, suggesting that the identified proteins are released specifically by the parasite upon germination. These could be proteins released by the sporoplasms on early infection or by the spore during germination. We retrieved 37 proteins from three quality-filtered replicates (Figure 6). Of these, 11/37 proteins are predicted by SignalP 4.1 to have a secretion signal and 17 are predicted by TargetP 1.1 to be directed to the secretory pathway; in some cases these predictions overlap (Figure 6). Proteins secreted to the extracellular medium range in size from 101 amino acids to 935 amino acids however there is no obvious correlation between size and the presence or absence of a predicted secretion signal (Figure 6). Five proteins are consistently present in all three replicates. Three of these have no similarity to any other proteins in the NCBI nr database (e<1×10^−5^) (SLOPH 477, 723, 762), while two of the proteins are found in other microsporidia (SLOPH 1766, 1854). One of these has features which make it particularly interesting in the context of potential effector proteins: it is part of a multigene family whose members have predicted Ricin-B-lectin domains and is found in several copies in *S. lophii*, as well as in several other microsporidian genomes (SLOPH 1766-see below). The other secreted protein shared with other microsporidia has significant sequence similarity to a spore wall protein (SWP7) in *Nosema bombycis* (SLOPH 1854).

Ten proteins are found in two of three replicates. Six of these proteins have no similarity to any proteins in the NCBI database (e<1×10^−5^) and are potentially unique to *S. lophii*, demonstrating that species-specific innovations may play a crucial role in *S. lophii* invasion of the host cell. Four proteins are found in *S. lophii* and other microsporidia, including SLOPH 1749, which contains an exonuclease/endonuclease/phosphatase domain, SLOPH 2344, a spore wall protein and another RICIN-B lectin domain protein (SLOPH 691 - see below). A chitin deacetylase, which is conserved across the Microsporidia, was also identified in 2/3 biological replicates. The orthologue of this protein has been studied in *E. cuniculi*. In this species it is expressed at high levels during sporogonial lifecycle stages and localizes to the endospore, where it accumulates in paramural bodies [55], [56]. However, later functional analysis demonstrated that the protein was unable deacetylate chitooligosaccharides or bind chitin or any glycans, suggesting a non-canonical function [57]. Urch *et al* speculate that this chitin deacetylase may have evolved from a carbohydrate active enzyme to a lectin and may bind carbohydrates in the *E. cuniculi* cell wall [57]. A total of five microsporidia-specific uncharacterized proteins were observed in at least 2/3 biological replicates (SLOPH 1854, 1766, 2344, 1749, 691) (Figure 6). Orthologues of these proteins display a varied taxonomic distribution and secretion prediction profile across microsporidia, although two of them - SLOPH 2344 and SLOPH 1749 - are present in all microsporidia examined (with the exception of *N. parisii*) and are predicted to be secreted in all species expect *E. intestinalis* and *E. bieneusi* (SLOPH 2344), or just *E. bieneusi* (SLOPH 1749) (Table S6). Some proteins lacking predicted secretion signals are potentially highly expressed proteins that are released during the germination protocol and are therefore potentially false positives, for example histone proteins and translation elongation factor which were found in single replicate. Others, that are present in 2 or 3 replicates, may be secreted via a non-canonical secretion signal not detected by bioinformatics prediction programs.

The *S. lophii*-specific proteins may be host-driven innovations that mediate interactions specifically with the fish host, representing cases of lineage-specific adaptation or microsporidian-specific proteins that are fast evolving and not easily recognized between species. Some of the identified proteins are part of multigene families with other homologs in the *S. lophii* genome (Starred in Figure 6, Table S7) though proteomic data correspond to one member of the family. In agreement with our transcriptome analysis, these results demonstrate the importance of uncharacterized, hypothetical proteins in microsporidian germination, host cell invasion and early infection. This approach also represents a powerful method for identifying the presumably small proportion of novel parasite effectors or virulence factors from among the hundreds, or thousands, of hypothetical proteins found in sequenced microsporidian genomes. The need for such streamlining approaches is obvious, as a genetic manipulation protocol for microsporidia is currently lacking and hence protein characterization relies on heterologous expression systems or indirect assays which are time consuming and not easily applied to a large number of proteins.

### A family of lectin-like proteins conserved in the Microsporidia

**Figure 7:**
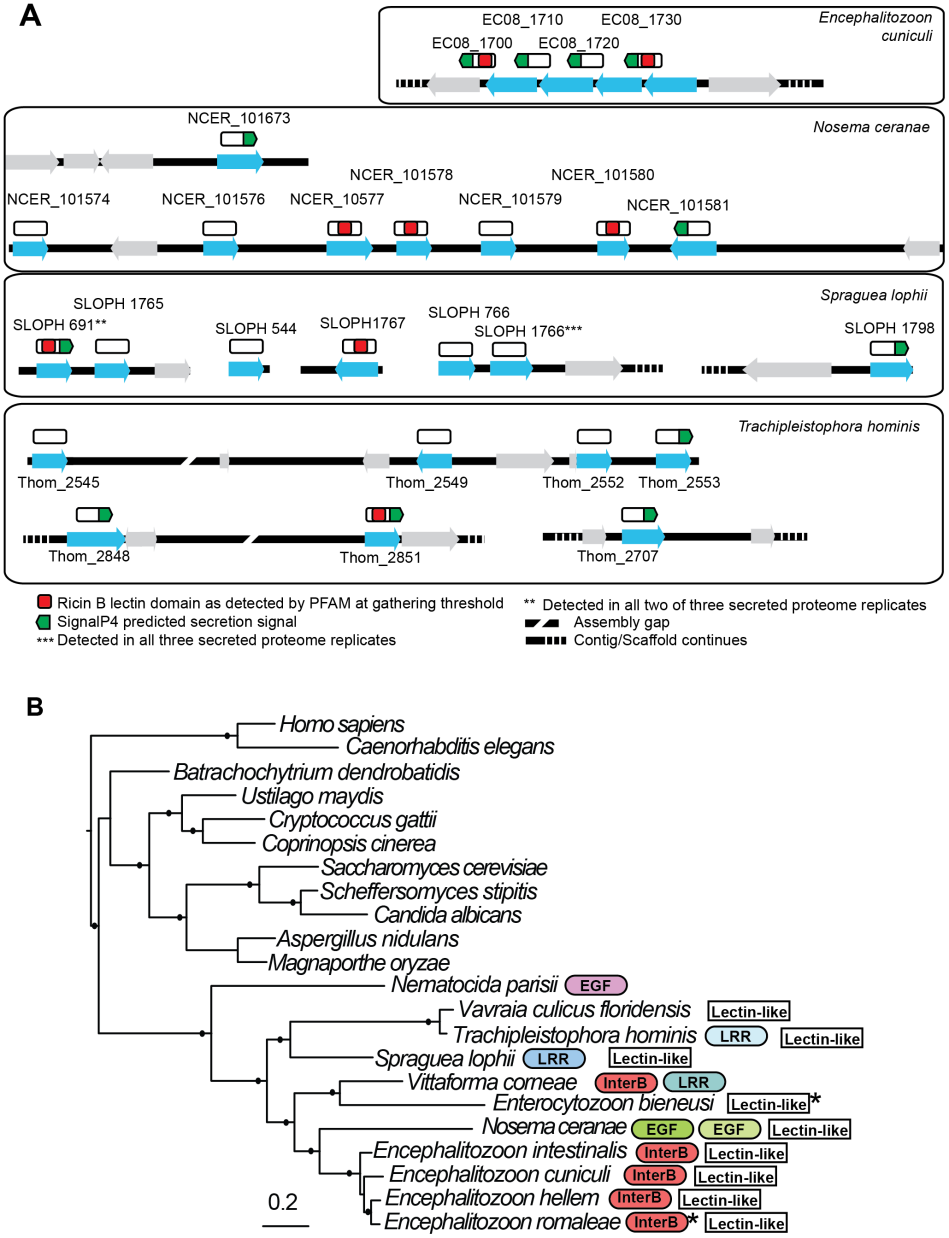
Genomic context of lectin-like proteins and phylogenetic distribution of expanded gene families in microsporidia. **A**. Putative lectin-like proteins are shown in their genomic context as blue arrows and unrelated flanking genes are shown as grey arrows. Above these (white rectangles) are shown hypothetical translated proteins (not to scale) to illustrate the positions of predicted motifs. The proteins SLOPH 691 and 1766 that were identified in our secretion proteomics were BLASTed against the *Spraguea lophii* genome at a cutoff of e<1×10^−5^ to identify other members of the lectin-like family. These proteins also show BLASTP similarity to lectin-like proteins in *E. cuniculi* and *N. ceranae*, as indicated. **B**. RAxML 8 protein phylogeny of microsporidia showing the phylogenetic context of *S. lophii*. Published expanded gene families were mapped onto this phylogeny, including InterB proteins [66], leucine rich-repeat proteins (LRR) [8], and other expanded gene families (EGF) as published [22], [23]. Stars indicate the detection of a single homolog of the protein in the genome. Shading is used to denote different families of apparently unrelated LRR proteins. Solid circles on branches indicate a posterior probability of 1 in our Bayesian phylogenetic analysis.

Two of the identified secreted proteins (SLOPH 1766 and SLOPH 691) are conserved in other microsporidia and show similarity to RICIN B-lectin proteins, with weakly conserved motifs involved in carbohydrate binding. Lectins have diverse roles in parasites, and can mediate adhesion of the parasite to the host cell during infection, but they can also play roles in immune evasion and their binding to host proteins can trigger different developmental pathways in the parasite [58], [59]. The members of this family form clusters in the genomes of *E. cuniculi* and *N. ceranae* (Figure 7A), reminiscent of clusters of effector proteins in fungal pathogens such as *Ustilago maydis* [60] and the RXLR and Crinkler effector families in oomycetes of the genus *Phytophthora* [61]. In *E. cuniculi*, four proteins are found in a single syntenic block, whereas in *N. ceranae*, a block of six proteins sit together in the genome with two others elsewhere. Interrogating the *E. cuniculi* genome, which has well-assembled chromosomes, these genes are not found at the end of chromosomes in the subtelomeric regions as is the case for effector proteins in other parasite species [62]. It is difficult to draw conclusions about the relative genomic location of the corresponding genes from our *S. lophii* data as several are found on short contigs, but we did identify two clusters of two genes (Figure 7A). A phylogeny of this protein family suggests that most of the paralogs within each species arose from species-specific duplications (Figure S3), again mirroring the pattern seen for effector families in other eukaryotic pathogens such as *Ustilago maydis* [60], *Phytophthora* [61] and *Trichomonas* [63]. The distribution of lectin-like proteins and expanded gene families were plotted onto a multigene phylogeny of microsporidia to give an overview of their distribution across the phylum (Figure 7B); the patchy distribution of these families suggests a process of differential loss and expansion during the radiation of microsporidia. In particular, we did not detect homologues of the lectin-like proteins in the *N. parisii* genome, suggesting that this family may have evolved after the divergence of *N. parisii* from the other sequenced microsporidia.

## Discussion

Although the *S. lophii* genome is larger than that of *E. cuniculi*, there is no clear trend towards a greater retention of broader ancestral metabolism. Like *T. hominis*, *S. lophii* has apparently lost the ability to biosynthesize isoprenoids, although it has retained more enzymes involved in other aspects of lipid metabolism. These results demonstrate that the loss of major biosynthetic pathways did not occur only in the common ancestor of all microsporidia, but has been an ongoing process throughout the evolution of the group, giving rise to important lineage-specific differences in metabolism.

Beyond reduction in genome size, a relative scarcity of introns is a conserved and striking feature of microsporidian genomes. The remaining introns tend to be short and, in many cases, located at the 5′ ends of genes encoding ribosomal proteins [5]. Our comparisons of splicing in *S. lophii* and *E. cuniculi* suggest that the molecular determinants of splicing efficiency are conserved in these distantly-related species, with longer, out-of-frame introns experiencing the highest levels of splicing. The observation that some inefficiently spliced, intron-like sequences can be read through without producing a truncated or frameshifted protein suggests a plausible, though speculative, mechanism by which introns could be lost from microsporidian genomes: once read-through is possible, subsequent deletions could reduce or remove the newly expressed insertion, resulting in complete loss of the former intronic sequence. We note, however, that the introns we compared in our assay are conserved between *S. lophii* and *E. cuniculi*, and so have not been lost in the period of time since the divergence of these species.

One of the most striking features of the *S. lophii* genome is the presence of a leucine rich repeat protein family, which may represent an expanded gene family of pathogenicity factors. Such expanded gene families are characteristic of other fungal pathogens such as *Batrachochytrium dendrobatidis* and *Blumeria graminis*, have also been reported in the microsporidia *T. hominis*, *E. cuniculi*, *Anncaliia algerae* and *Vittaforma corneae* [8], [64]–[66], and are also considered of importance in host-parasite interactions more generally [67]. Whilst the *S. lophii* members of this family have predicted N-terminal secretion signals, there is no evidence of an obvious conserved peptide motif involved in directing protein secretion into the host such as the oomycete crinkler motifs or the conserved tripeptide motif found in *B. graminis* which could help to define the microsporidian secretome [65], [68].

In addition to the conserved microsporidian proteome and an expanded complement of LRR proteins, *S. lophii* encodes a large number of predicted hypothetical proteins. Some of these were only expressed at low levels in our transcriptome analysis, potentially representing false positive ORF calls, but there were also a significant number of highly expressed microsporidia-specific transcripts and together with *S. lophii*-specific transcripts, these made up 39.1% of the total expression in *S. lophii* spores (Figure 4). Thus, lineage-specific or fast evolving proteins likely play an important role in *S. lophii* biology, and the same may well be true for all microsporidia. Although we now have genome sequences for a number of microsporidia, our understanding of their basic biology, pathogenicity and host interactions is limited by the current lack of a genetic manipulation system for these intracellular parasites. Recent work has shown that identification of proteins bearing secretion signals through bioinformatics can reveal interesting strategies by which microsporidia may alter the host environment in their favor. Cuomo *et al.* have identified functional secretion signals in a family of microsporidian hexokinases; when secreted, these effectors may stimulate host metabolism, providing more metabolites for the parasite [22]. These results demonstrate the utility of bioinformatics in predicting signal peptides and identifying microsporidian proteins that may be targeted to the cell surface or secreted. However, signal peptide prediction tools are trained on model eukaryotes, and their accuracy in predicting the full secretome from the highly divergent sequences of microsporidia is unclear. Even when these signals are accurately identified, heterologous characterization is laborious and expensive. One way forward may be through alternatives to genetic transformation, such as those we have explored in this article. Here we have used secretion proteomics to detect candidate virulence factors that are likely to be secreted from the parasite as it germinates, leaves the polar tube and enters the host cell. Two of these identified secreted proteins are a part of a family that is broadly conserved across the Microsporidia, with local duplications giving rise to syntenic blocks of family members in several microsporidian genomes. Based on their sequence similarity to lectin domains, they may be involved in binding to carbohydrates on host proteins, and their conservation suggests they play an important general role in microsporidian parasitism. We combined our secretion proteomics with an analysis of the *S. lophii* transcriptome, which identified a further set of highly expressed, microsporidia-specific hypotheticals. These proteins are ideal candidates for further characterization to better understand the molecular basis of parasitism in the Microsporidia, both within the parasite cell and in its host interactions.

## Materials and Methods

### Spore purification

DNA was extracted from two separate clusters of cysts both collected from local Monkfish (*Lophius piscatorius*) landed at either Plymouth or Brixham caught in the North Atlantic. Spores were separated from fish material in a three-step process. Xenomas filled with microsporidia were removed from the fish tissue manually. Samples were then soaked overnight at 4°C in EDTA (10 mM) containing Triton X 100 at 0.05% and trypsin 0.025% (w/v). The samples were then homogenized in a glass homogenizer until they formed a fine suspension, and washed three times in sterile PBS. The spore suspension was then cleaned by centrifugation through 100% Percoll (Sigma) at 4°C at 1600×g for 15 minutes. Samples were further washed three times in sterile 1× PBS before resuspension and storage at 4°C in 1 ml of sterile 1× PBS with addition of an antibiotic cocktail of 10 µg/ml Ampicillin, Penicillin/Streptomycin and Kanamycin.

### DNA extraction and library preparation

Aliquots of 200 µl of purified spores were resuspended in 400 µl TE 10/1 pH 7.5 and ground in a pestle and mortar in liquid nitrogen for 15 minutes. Powdered frozen material was transferred to 800 µl phenol (pH 7.9) and mixed by inversion then centrifuged at 10,000×g for 10 minutes. 400 µl chloroform was added to the aqueous supernatant and this was centrifuged at 10,000×g for 5 minutes. DNA was precipitated from the aqueous layer using a standard ethanol precipitation protocol [69].

### Preparation of DNA library for Illumina sequencing

For sample one, the DNA was sheared using a Covalis Nebuliser. Sheared fragments of approximately 400 bp in size were selected using a polyacrylamide gel and used to create a library using the Illumina paired-end sample preparation protocol (revision A June 2008). A single lane of paired-end 36 bp sequence reads was sequenced. For sample two, DNA was sheared by Biorupter sonication and fragments of approximately 600 bp were selected by polyacrylamide electrophoresis for library preparation as above. A single lane of paired-end reads of 76 bp was sequenced giving a total of 325 Mb of data.

### Data assembly and annotation

The data from the two runs were pooled and assembled together using Velvet 0.7.50 using a kmer length of 31 and coverage cutoff of 5. Estimated expected kmer coverage using automatic calibration was 6. Contigs of 500 bp or larger were retained and analysed further. This resulted in a set of 3080 contigs, which showed a bimodal distribution of GC content with peaks at approximately 23% GC and 58% GC, indicative of DNA from a second organism (based on sequence identity, a close relative or strain of *Pseudomonas fluorescens*). A histogram of GC content revealed two distinct distributions with little overlap (Figure S4); we therefore discarded all contigs with GC content greater than 40% as potential *Pseudomonas* contaminants. All retained contigs were used to BlastN search gi229587578 *Pseudomonas fluorescens* SBW25 chromosome and gi146343893 *Pseudomonas fluorescens* SBW25 plasmid pQBR103, to verify that there was no *P. fluorescens* DNA in the remaining contigs. Removed high GC contigs were also used to BLAST search the same genomes and any contigs with a BLASTN hit value higher than 1×10^−30^ were used to BLASTX search the same microsporidia used in our comparative analysis to verify that there was no obvious microsporidian content. Remaining contigs were parsed to find unknown stretches of DNA left by paired-end assembly of 5 base pairs or more and split at these points. This left a set of 1392 contigs that were further analyzed. These contigs were annotated using Artemis 13.2.0. All ORFs of 100 amino acids or more were analyzed by BLASTP and PFAM search and an annotation given on the basis of these searches [35]. Each contig was searched for the presence of tRNAs using tRNAscan-SE v.1.23 [70], which were annotated onto the contigs using Artemis.

### OrthoMCL and BLAST analysis

56274 proteins sequences from eight species (predicted proteomes of *H. sapiens*, *S. cerevisiae*, *N. ceranae*, *E. bieneusi*, *E. cuniculi*, *N. parisii*, *T. hominis* and those 2499 *S. lophii* proteins without predicted frameshifts) were clustered using OrthoMCL: BLAST e-value cutoff = 1e-5, Inflation value = 1.5. Output files were parsed and sorted into categories shown in Figure 2A.

### *In vitro* germination

300 µl of purified spores were washed 3 times in 1× PBS. A non-germinated control sample was then treated with 100 µl of 0.25 mM EGTA per 100 µg of spores to inhibit germination. The sample to be germinated was treated with 100 µl of 0.5 M Gly-Gly buffer (pH 7.0) per 100 µg of spores and incubated at room temperature for 30 minutes. 40 µl of Calcium Ionophore A23187 (1 µg/µl) (Sigma-Aldrich) dissolved in DMSO was added to the sample followed immediately by 40 µl of 0.5 M Gly-Gly buffer (pH 9.0) per 100 µg of spores. Germination was verified instantly by light microscopy and efficiency was estimated to reach a maximum of 80%.

### RNA preparation for sequencing

200 µl of germinated *S. lophii* spores were frozen with liquid nitrogen and disrupted manually using a pestle and mortar. RNA was then extracted following the standard TRIzol (Invitrogen) protocol. RNA was resuspended in 50 µl Milli-Q water (Millipore) and quantified as 2260 ng/ul (absorbance 260/280 = 2.12; 260/230 = 2.41). RNA integrity was verified on 1.5% TAE agarose gel prior to sequencing. PolyA RNA was isolated from 4 µg total RNA and the TruSeq library was prepared according to the Illumina TruSeq RNA sample preparation guide (Part # 15008136 Rev. A November 2010). 15 cycles of PCR were used to amplify the library and 5 µl of the 6 nM library was loaded onto a flow cell with two other TruSeq libraries and run on the HiSeq 2000 with a paired-end 100 bp run.

### Transcriptome assembly and analysis

The raw Illumina reads obtained from RNA sequencing were filtered with fastq-mcf [71] to remove adapter sequences and low quality reads (with a quality score <28). The filtered reads were assembled using the Trinity package [43]. Reads were mapped back onto the transcriptome assembly using Bowtie [72], and the abundance of each transcript was estimated using RSEM [44].

### Identification of proteins secreted into the extracellular medium during spore germination

500 µl of purified *S. lophii* spores were germinated using the protocol described previously. Germinated *S. lophii* spores along with non-germinated control were then spun at 12,000×g for 15 minutes at 4°C. The resultant supernatant was then collected and concentrated using Millipore Amicon 3 kDa centrifugal concentration column at 2,000×g for 30 minutes at 4°C. The concentrated extracellular protein was then quantified and checked on 12% SDS gels. Complex mixtures of extracellular secreted proteins from germinated and non-germinated samples were sent for analysis on an 6520 accurate mass quadrupole time of flight (Q-TOF) mass spectrometer (Agilent Technologies) and resultant masses were searched against our translated *S. lophii* genome database. The experiment was conducted in triplicate and any protein without two distinct peptide hits and a percent score peak intensity (% SPI) of ≥60% was removed from the analysis.

## Supporting Information

Figure S1 A glutamate-ammonia ligase *in S. lophii*. **A**. Phylogeny of glutamate-ammonia ligase. PhyloBayes phylogenetic tree of eukaryotic glutamate-ammonia ligase proteins using the C60 empirical mixture model. Black circles show nodes with posterior probability equal to 0.99 and squares show posterior probability support of 1. **B**. Genomic context of the glutamate ammonia ligase gene. The gene is located between genes found in other microsporidian genomes. Bars and agarose gel images above show the sizes of PCR products amplified spanning the glutamate ammonia ligase gene and adjacent genes in the genome. These observations suggest that this is a *bona fide S. lophii* gene potentially acquired by horizontal transfer.
(TIF)

Figure S2 SDS-PAGE of *S. lophii* proteins secreted into the extracellular medium during germination. Protein from extracellular medium following germination was loaded onto a 12% SDS gel along with an *S. lophii* whole cell protein control. *S. lophii* hypothetical proteins (SLOPH 2344, SLOPH 477 and SLOPH 1709) were identified in the extracellular medium following band excision and mass spectrometry. SLOPH 2344 is predicted to be secreted to the extracellular environment by both SignalP and TargetP. No peptide hits were identified in the N terminal of the protein suggesting signal peptide cleavage may be responsible for the lower than predicted molecular weight visualized on SDS-PAGE.
(TIF)

Figure S3 Phylogeny of microsporidian lectin-like proteins. PhyloBayes (C20 model) tree of lectin-like proteins from representative microsporidia. Support values are provided as Bayesian posterior probabilities.
(TIF)

Figure S4 Histogram of GC content of sequenced contigs. The chosen GC cutoff point for identifying contaminant contigs is indicated by an arrow.
(TIF)

Table S1 Presence and absence of enzymes in selected metabolic pathways in our *S. lophii* genome survey and across Microsporidia. Table expanded from Keeling *et al* [21].
(XLSX)

Table S2 Classification of *S. lophii* predicted open reading frames according to OrthoMCL analysis.
(DOCX)

Table S3 List of conserved leucine rich proteins in *S. lophii* and other microsporidian genomes.
(XLSX)

Table S4 List of genes identified from RNA transcripts alone that are not included in our genomic data.
(DOCX)

Table S5 A list of proteins identified from germinated and non-germinated *S. lophii* spores.
(DOCX)

Table S6 Properties of putative *S. lophii* secreted hypothetical proteins in other microsporidian species.
(XLSX)

Table S7 Expanded gene families that have proteins identified in our germination secretome.
(DOCX)
