# Supplementary figures and images for: The Genome of Spraguea lophii and the Basis of Host-Microsporidian Interactions
Source: PLoS Genet. 2013 Aug 22;9(8):e1003676. doi: 10.1371/journal.pgen.1003676 (PMC3749934; doi:10.1371/journal.pgen.1003676)

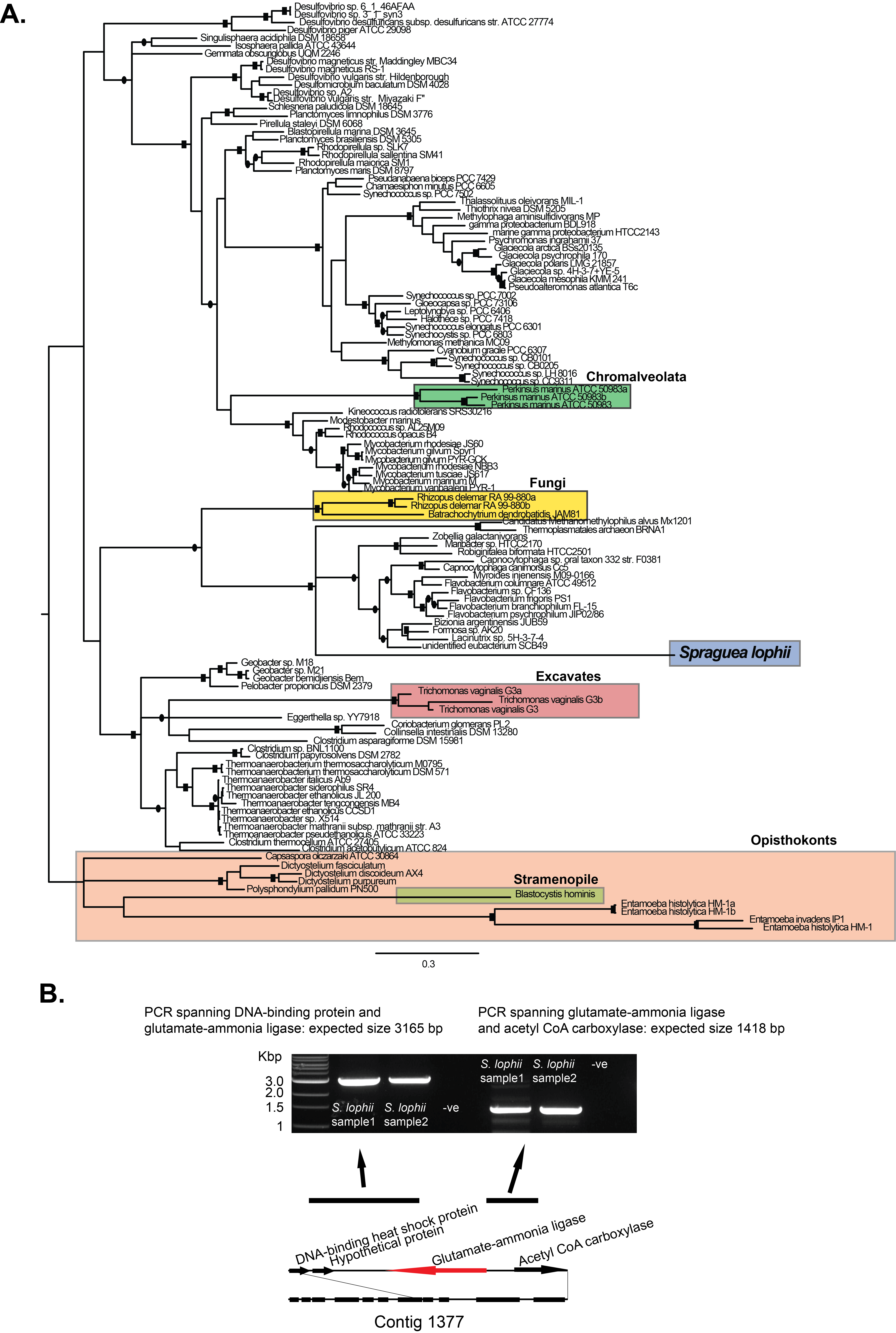

Supplement: Figure S1 — A glutamate-ammonia ligase in S. lophii. A. Phylogeny of glutamate-ammonia ligase. PhyloBayes phylogenetic tree of eukaryotic glutamate-ammonia ligase proteins using the C60 empirical mixture model. Black circles show nodes with posterior probability equal to 0.99 and squares show posterior probability support of 1. B. Genomic context of the glutamate ammonia ligase gene. The gene is located between genes found in other microsporidian genomes. Bars and agarose gel images above show the sizes of PCR products amplified spanning the glutamate ammonia ligase gene and adjacent genes in the genome. These observations suggest that this is a bona fide S. lophii gene potentially acquired by horizontal transfer. (TIF) [file pgen.1003676.s001.tif]

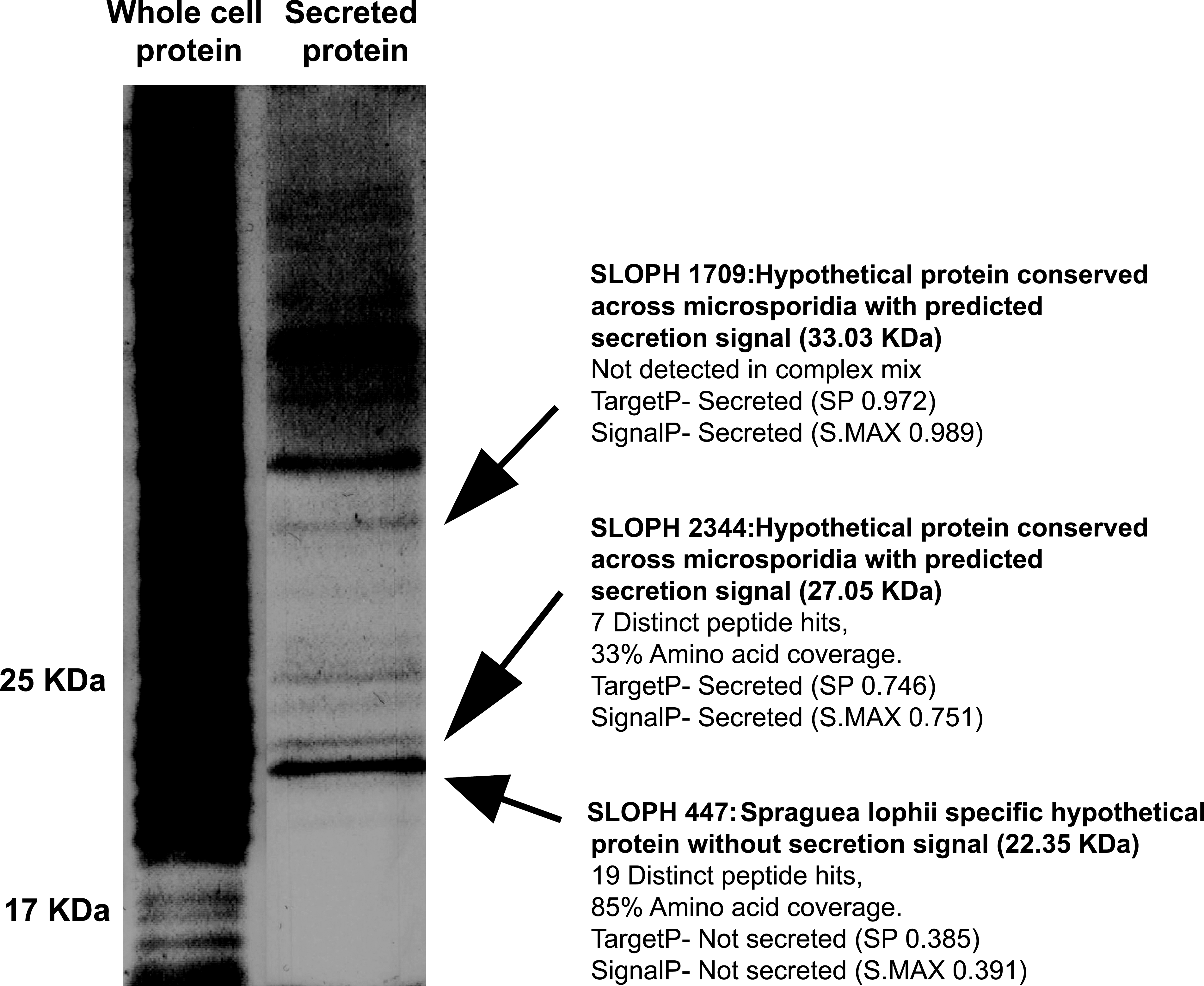

Supplement: Figure S2 — SDS-PAGE of S. lophii proteins secreted into the extracellular medium during germination. Protein from extracellular medium following germination was loaded onto a 12% SDS gel along with an S. lophii whole cell protein control. S. lophii hypothetical proteins (SLOPH 2344, SLOPH 477 and SLOPH 1709) were identified in the extracellular medium following band excision and mass spectrometry. SLOPH 2344 is predicted to be secreted to the extracellular environment by both SignalP and TargetP. No peptide hits were identified in the N terminal of the protein suggesting signal peptide cleavage may be responsible for the lower than predicted molecular weight visualized on SDS-PAGE. (TIF) [file pgen.1003676.s002.tif]

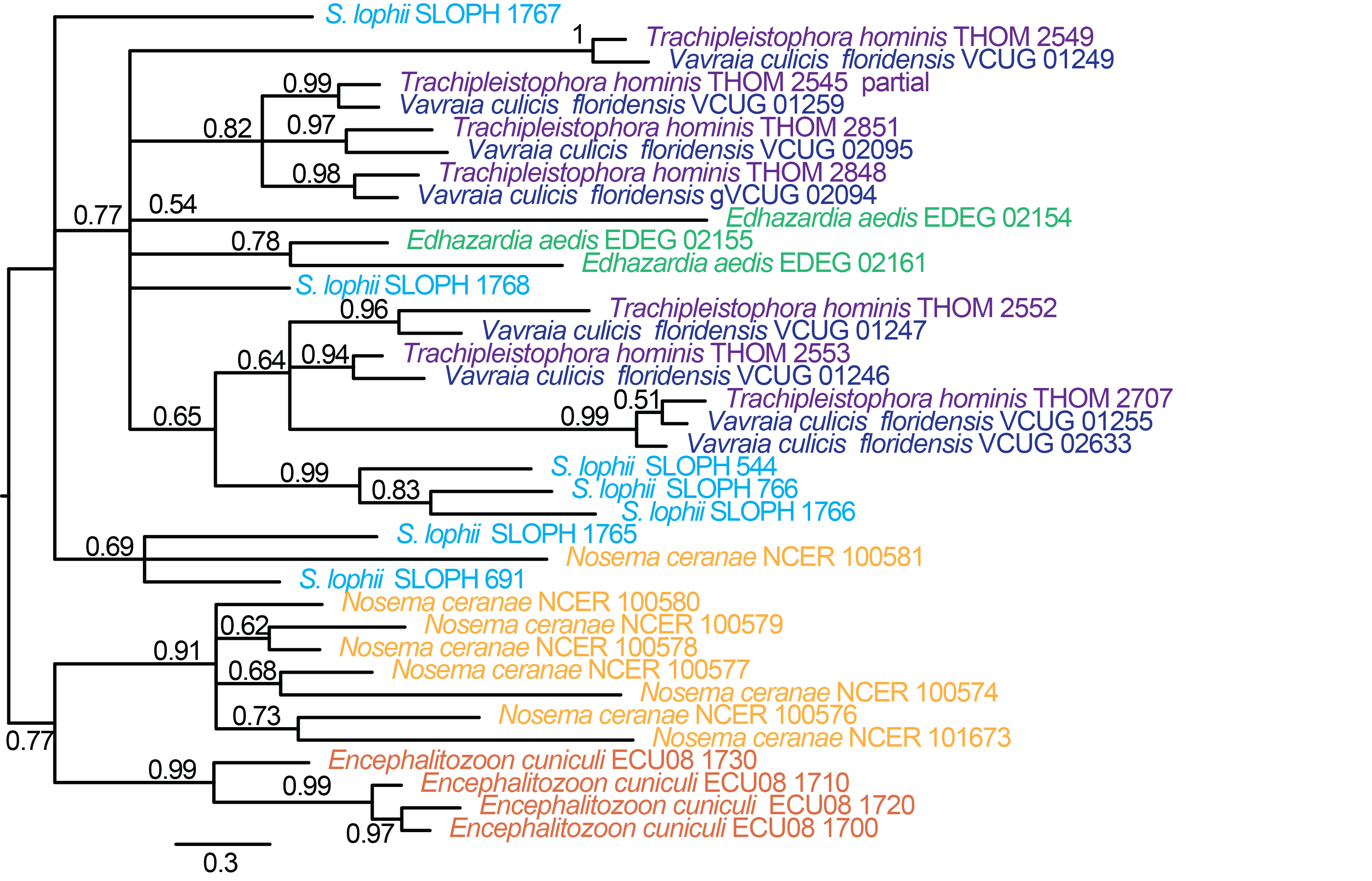

Supplement: Figure S3 — Phylogeny of microsporidian lectin-like proteins. PhyloBayes (C20 model) tree of lectin-like proteins from representative microsporidia. Support values are provided as Bayesian posterior probabilities. (TIF) [file pgen.1003676.s003.tif]

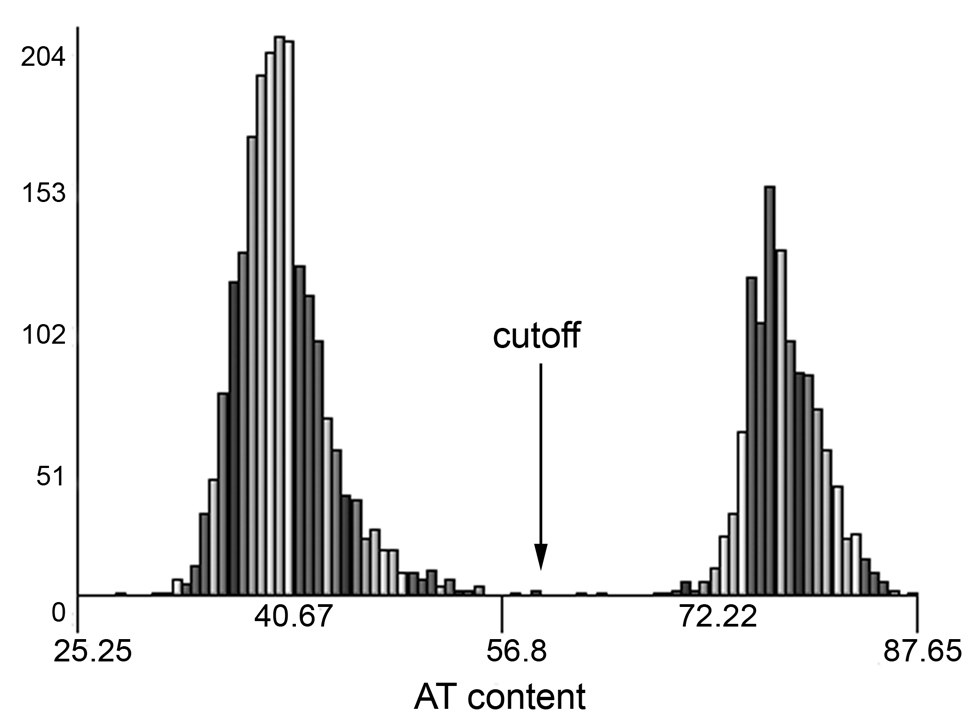

Supplement: Figure S4 — Histogram of GC content of sequenced contigs. The chosen GC cutoff point for identifying contaminant contigs is indicated by an arrow. (TIF) [file pgen.1003676.s004.tif]
