# Supplementary material for: The Genome of Spraguea lophii and the Basis of Host-Microsporidian Interactions
Source: PLoS Genet. 2013 Aug 22;9(8):e1003676. doi: 10.1371/journal.pgen.1003676 (PMC3749934; doi:10.1371/journal.pgen.1003676)
Supplement: Table S2 — Classification of S. lophii predicted open reading frames according to OrthoMCL analysis. (DOCX) [file pgen.1003676.s006.docx]

**Supplementary Table 2:**

56274 proteins sequences from eight species were clustered using OrthoMCL: BLAST Evalue cutoff = 1e-5, Inflation value = 1.5. 46419 of the proteins were clustered into groups of orthologues, leaving 9855 singletons. For *S. lophii* - total proteome = 2499 seqs., 1761 sequences in clusters, 738 singletons. Due to truncated genes and therefore some truncated hypothetical translations in this and other microsporidian sequencing projects, some proteins fall into unexpected categories at the set parameters.

*Spraguea lophii* proteins that are also found in *Homo sapiens*, *Saccharomyces cerevisiae*, *Nematocida parisii*, *Trachipleistophora hominis*, *Enterocytozoon bieneusi*, *Encephalitozoon cuniculi*, and *Nosema ceranae*:

| SLOPH_2 | 0-sialoglycoprotein endopeptidase |
| --- | --- |
| SLOPH_3 | 1-acyl-sn-glycerol-3-phosphate acyltransferase |
| SLOPH_2369 | 14-3-3 protein |
| SLOPH_5 | 19S proteasome regulatory subunit Rpn6 |
| SLOPH_6 | 20S proteasome component |
| SLOPH_7 | 20S proteasome component beta 3 |
| SLOPH_8 | 20S proteasome core particle subunit beta 5 |
| SLOPH_9 | 20S proteasome subunit |
| SLOPH_10 | 20s proteasome subunit beta type-1 |
| SLOPH_11 | 26S protease regulatory subunit 6 |
| SLOPH_12 | 26S protease regulatory subunit 6a |
| SLOPH_13 | 26S protease regulatory subunit 8 |
| SLOPH_14 | 26S protease regulatory subunit S10 |
| SLOPH_15 | 26S proteasome alpha-type subunit PRE9 |
| SLOPH_17 | 26S proteasome non-ATPase regulatory subunit |
| SLOPH_2184 | 26S proteasome regulatory complex component |
| SLOPH_18 | 26s proteasome regulatory subunit |
| SLOPH_2280 | 26S proteasome regulatory subunit |
| SLOPH_20 | 26S proteasome regulatory subunit 4, partial |
| SLOPH_21 | 26S proteasome regulatory subunit RPN11 |
| SLOPH_25 | 40S ribosomal protein S0 |
| SLOPH_27 | 40S ribosomal protein S11 |
| SLOPH_31 | 40S ribosomal protein S15A |
| SLOPH_2103 | 40S ribosomal protein S16 |
| SLOPH_33 | 40S ribosomal protein S18 |
| SLOPH_34 | 40S Ribosomal protein S19, partial |
| SLOPH_2189 | 40S ribosomal protein S2 |
| SLOPH_35 | 40S ribosomal protein S20 |
| SLOPH_40 | 40S ribosomal protein S3 |
| SLOPH_43 | 40S ribosomal protein S4 |
| SLOPH_44 | 40S ribosomal protein S5 |
| SLOPH_45 | 40S ribosomal protein S6 |
| SLOPH_47 | 40S ribosomal protein S8 |
| SLOPH_48 | 40S ribosomal protein S9 |
| SLOPH_49 | 5'- 3'exoribonuclease |
| SLOPH_53 | 60S acidic ribosomal protein P0 |
| SLOPH_55 | 60S ribosomal protein L10a |
| SLOPH_56 | 60S ribosomal protein L11 |
| SLOPH_57 | 60S ribosomal protein L12 |
| SLOPH_32 | 60S ribosomal protein L13 |
| SLOPH_58 | 60S ribosomal protein L17 |
| SLOPH_59 | 60S ribosomal protein L18 |
| SLOPH_61 | 60S ribosomal protein L2 |
| SLOPH_62 | 60s ribosomal protein L21 |
| SLOPH_2195 | 60S ribosomal protein L24 |
| SLOPH_65 | 60S ribosomal protein L26 |
| SLOPH_66 | 60S ribosomal protein L27 |
| SLOPH_69 | 60S ribosomal protein L3 |
| SLOPH_68 | 60S ribosomal protein L3 |
| SLOPH_73 | 60S ribosomal protein L4 |
| SLOPH_75 | 60S ribosomal protein L5 |
| SLOPH_2210 | 60S ribosomal protein L6 |
| SLOPH_76 | 60S ribosomal protein L7 |
| SLOPH_77 | 60S ribosomal protein L7A |
| SLOPH_81 | AAA ATPase |
| SLOPH_82 | AAA ATPase |
| SLOPH_88 | ABC transporter |
| SLOPH_90 | ABC transporter |
| SLOPH_91 | ABC transporter |
| SLOPH_85 | ABC transporter |
| SLOPH_2319 | ABC transporter |
| SLOPH_2598 | ABC transporter protein |
| SLOPH_2591 | ABC transporter, partial |
| SLOPH_2559 | ABC transporter, partial |
| SLOPH_94 | ABC-transporter like protein, partial |
| SLOPH_95 | ABC-transporter like protein, partial |
| SLOPH_99 | Acetyl-coenzyme A synthetase |
| SLOPH_101 | Actin |
| SLOPH_104 | Acyltransferase |
| SLOPH_110 | ADP-ribosylation factor 4 |
| SLOPH_119 | Alanyl-tRNA synthetase |
| SLOPH_2480 | Alpha subunit of translation initiation factor eIF2, partial |
| SLOPH_122 | Alpha tubulin |
| SLOPH_2165 | Anaphase promoting complex subunit (Cdc27/Nuc2/BimAfamily) |
| SLOPH_2597 | Anion-transporting ATPase-/ParA/MinD ATPase likeprotein |
| SLOPH_2593 | Arginine/alanine aminopeptidase, partial |
| SLOPH_2665 | Arginine/alanine aminopeptidase, partial |
| SLOPH_135 | Arginyl-tRNA synthetase |
| SLOPH_141 | ASF1 anti-silencing function 1 like protein |
| SLOPH_143 | Asparaginyl-tRNA synthetase |
| SLOPH_2119 | ATP binding protein |
| SLOPH_150 | ATP-dependent DNA helicase recQ |
| SLOPH_151 | ATP-dependent helicase HrpA |
| SLOPH_2277 | ATP-dependent RNA helicase |
| SLOPH_158 | Beta tubulin |
| SLOPH_162 | BRIX domain protein |
| SLOPH_163 | Brix domain-containing protein |
| SLOPH_164 | Brix domain-containing protein 1 |
| SLOPH_167 | Bromodomain protein |
| SLOPH_177 | Calponin domain protein |
| SLOPH_179 | Calponin/transgelin |
| SLOPH_182 | CAP-Gly Cytoskeleton-associated protein |
| SLOPH_184 | Casein kinase 1 |
| SLOPH_2154 | Casein kinase II alpha subunit |
| SLOPH_2125 | Casein kinase II beta 2 subunit |
| SLOPH_187 | CCAAT-binding transcription factor |
| SLOPH_188 | CCAAT-binding transcription factor subunit A |
| SLOPH_189 | CCAAT-box binding factor |
| SLOPH_190 | ccr4-associated factor |
| SLOPH_195 | Cell differentiation protein Rcd1 |
| SLOPH_2359 | Chromosome condensation complex Condensin subunit H |
| SLOPH_208 | Chromosome condensation complex condensin, partial |
| SLOPH_209 | Chromosome segregation protein |
| SLOPH_210 | Chromosome segregation protein |
| SLOPH_212 | Cleavage and polyadenylation specificity factor |
| SLOPH_213 | Cleavage and polyadenylation specificity factor, partial |
| SLOPH_215 | Cleavage stimulation factor |
| SLOPH_216 | Coatomer alpha subunit, partial |
| SLOPH_2234 | Coatomer complex beta subunit |
| SLOPH_220 | Condensin complex component, partial |
| SLOPH_248 | Cyclin dependent protein kinase |
| SLOPH_247 | Cyclin, partial |
| SLOPH_249 | Cysteine desulfurase |
| SLOPH_250 | Cysteinyl-tRNA synthetase |
| SLOPH_256 | Cytosolic Fe-S cluster assembly factor NBP35 |
| SLOPH_259 | Dead box ATP-dependent RNA helicase |
| SLOPH_262 | DEAD/DEAH box helicase |
| SLOPH_264 | DEAD/DEAH box helicase |
| SLOPH_267 | Deoxyhypusine synthase |
| SLOPH_268 | Deoxyhypusine synthase |
| SLOPH_271 | DHHC zinc finger protein |
| SLOPH_276 | Dimethyladenosine transferase |
| SLOPH_277 | Diphthamide biosynthesis protein 1 |
| SLOPH_279 | Diphthine synthase |
| SLOPH_282 | DNA helicase |
| SLOPH_283 | DNA ligase |
| SLOPH_284 | DNA mismatch repair protein msh-2 |
| SLOPH_2272 | DNA polymerase alpha-primase complex polymerase-associated subunit B |
| SLOPH_289 | DNA polymerase delta catalytic subunit |
| SLOPH_291 | DNA polymerase epsilon, partial |
| SLOPH_292 | DNA primase small subunit |
| SLOPH_295 | DNA repair helicase RAD25 protein |
| SLOPH_2475 | DNA repair helicase rad3 |
| SLOPH_294 | DNA repair helicase, partial |
| SLOPH_299 | DNA repair protein rad18 |
| SLOPH_302 | DNA repair protein RAD50 |
| SLOPH_303 | DNA repair protein RAD51 |
| SLOPH_304 | DNA replication complex GINS protein PSF2 |
| SLOPH_305 | DNA replication factor C |
| SLOPH_306 | DNA replication factor C subunit |
| SLOPH_307 | DNA replication licensing factor MCM |
| SLOPH_308 | DNA replication licensing factor MCM2 |
| SLOPH_309 | DNA replication licensing factor MCM3 |
| SLOPH_2358 | DNA replication licensing factor MCM4 component |
| SLOPH_310 | DNA replication licensing factor MCM6 |
| SLOPH_312 | DNA topoisomerase II |
| SLOPH_313 | DNA topoisomerase III, partial |
| SLOPH_314 | DNA-binding heat shock factor |
| SLOPH_315 | DNA-directed DNA polymerase alpha |
| SLOPH_2329 | DNA-directed RNA polymerase I |
| SLOPH_316 | DNA-directed RNA polymerase I largest subunit |
| SLOPH_2255 | DNA-directed RNA polymerase I, subunit M |
| SLOPH_2481 | DNA-directed RNA polymerase II complex subunit Rpb2 |
| SLOPH_320 | DNA-directed RNA polymerase II largest subunit |
| SLOPH_2715 | DNA-directed RNA polymerase II subunit Rpb10 |
| SLOPH_2262 | DNA-directed RNA polymerase III 34kDa subunit |
| SLOPH_2615 | DNA-directed RNA polymerase III Rpc2 |
| SLOPH_2428 | DNA-directed RNA polymerase III subunit RPC1 |
| SLOPH_2235 | DNA-directed RNA polymerases III subunit K |
| SLOPH_326 | Dolichol-phosphate mannosyltransferase |
| SLOPH_2273 | Dolichyl-phosphate-mannose-proteinmannosyltransferase |
| SLOPH_327 | Dopey family protein |
| SLOPH_330 | DWNN domain protein |
| SLOPH_333 | Elongation factor Tu GTP binding domain protein |
| SLOPH_340 | ER lumen protein retaining receptor |
| SLOPH_342 | ERCC4 domain nuclease |
| SLOPH_344 | Eukaryotic peptide chain release factor subunit 1 |
| SLOPH_2456 | Eukaryotic translation initiation factor 3 subunitI EIF-3I |
| SLOPH_346 | Eukaryotic translation initiation factor 4A |
| SLOPH_347 | Eukaryotic translation initiation factor 5 |
| SLOPH_348 | Eukaryotic translation initiation factor 6 |
| SLOPH_349 | Eukaryotic translation initiation factor eIF2A |
| SLOPH_353 | Exonuclease |
| SLOPH_355 | Exoribonuclease II |
| SLOPH_356 | Exosome complex exonuclease rrp4 |
| SLOPH_358 | Exportin |
| SLOPH_359 | EXS domain containing protein |
| SLOPH_360 | EXS family protein |
| SLOPH_2215 | FEN1-like protein |
| SLOPH_367 | Fibrillarin |
| SLOPH_371 | Forkhead domain-containing protein |
| SLOPH_2684 | FtsJ, 23S rRNA methylase |
| SLOPH_2620 | gamma glutamyltranspeptidase |
| SLOPH_382 | Gamma tubulin, partial |
| SLOPH_381 | Gamma-glutamyltranspeptidase |
| SLOPH_383 | Gar1 small nucleolar RNP |
| SLOPH_385 | GATA Zn-finger-containing transcription factor |
| SLOPH_387 | GATA-binding transcription factor |
| SLOPH_389 | Geranylgeranyltransferase |
| SLOPH_2164 | Glucosamine 6-phosphate N-acetyltransferase |
| SLOPH_390 | Glucosamine-fructose-6-phosphate aminotransferase |
| SLOPH_391 | Glucose-6-phosphate dehydrogenase |
| SLOPH_2621 | glucose-repressible alcohol dehydrogenase transcriptional effector |
| SLOPH_393 | Glutaminyl-tRNA synthetase |
| SLOPH_395 | Glutaredoxin |
| SLOPH_397 | Glutathione peroxidase |
| SLOPH_399 | Glyceraldehyde-3-phosphate dehydrogenase |
| SLOPH_401 | Glycerophosphoryl diester phosphodiesterase |
| SLOPH_404 | glycyl-tRNA synthetase |
| SLOPH_1656 | GPI-anchored wall transfer protein 1 like protein |
| SLOPH_409 | GTP binding protein |
| SLOPH_2200 | GTP-binding protein |
| SLOPH_410 | GTP-binding protein |
| SLOPH_412 | GTP-binding protein sar1 |
| SLOPH_413 | GTP-binding protein YPT1 |
| SLOPH_2232 | Guanine nucleotide binding protein beta subunit |
| SLOPH_414 | Guanylate kinase |
| SLOPH_417 | Heat shock protein 90, partial |
| SLOPH_419 | Heat shock transcription factor |
| SLOPH_420 | HECT-domain ubiquitin-protein ligase E3 |
| SLOPH_421 | Helicase MOT1-like protein, partial |
| SLOPH_2623 | Histone acetyltransferase SAS2 |
| SLOPH_425 | Histone deacetylase 1 |
| SLOPH_427 | Histone deacetylase domain containing protein |
| SLOPH_431 | Histone H3 |
| SLOPH_430 | Histone H3 |
| SLOPH_432 | Histone H3 protein |
| SLOPH_434 | Histone transcription regulator, partial |
| SLOPH_436 | HIT domain protein |
| SLOPH_449 | Hsp70 protein |
| SLOPH_451 | Hydroxyacylglutathione hydrolase |
| SLOPH_1651 | hypothetical protein SLOPH 1651 |
| SLOPH_1661 | hypothetical protein SLOPH 1661 |
| SLOPH_1665 | hypothetical protein SLOPH 1665 |
| SLOPH_1701 | hypothetical protein SLOPH 1701, partial |
| SLOPH_1718 | hypothetical protein SLOPH 1718, partial |
| SLOPH_1728 | hypothetical protein SLOPH 1728 |
| SLOPH_1738 | hypothetical protein SLOPH 1738, partial |
| SLOPH_1761 | hypothetical protein SLOPH 1761 |
| SLOPH_1762 | hypothetical protein SLOPH 1762 |
| SLOPH_1771 | hypothetical protein SLOPH 1771 |
| SLOPH_1783 | hypothetical protein SLOPH 1783 |
| SLOPH_1790 | hypothetical protein SLOPH 1790 |
| SLOPH_1810 | hypothetical protein SLOPH 1810 |
| SLOPH_1846 | hypothetical protein SLOPH 1846 |
| SLOPH_2123 | hypothetical protein SLOPH 2123 |
| SLOPH_2212 | hypothetical protein SLOPH 2212 |
| SLOPH_2257 | hypothetical protein SLOPH 2257 |
| SLOPH_2336 | hypothetical protein SLOPH 2336 |
| SLOPH_2361 | hypothetical protein SLOPH 2361 |
| SLOPH_2638 | hypothetical protein SLOPH 2638, partial |
| SLOPH_2710 | hypothetical protein SLOPH 2710, partial |
| SLOPH_822 | hypothetical protein SLOPH 822 |
| SLOPH_1228 | Importin alpha subunit |
| SLOPH_1230 | Inorganic pyrophosphatase |
| SLOPH_1233 | Isoleucine tRNA synthetase |
| SLOPH_1236 | IWS1 transcription elongation factor |
| SLOPH_1237 | Karyopherin |
| SLOPH_1238 | KH domain containing protein |
| SLOPH_2286 | kinesin |
| SLOPH_1245 | La domain protein |
| SLOPH_1206 | Leucine rich repeat protein |
| SLOPH_1328 | Lipopolysaccharide kinase domain protein, partial |
| SLOPH_1331 | Long-chain-fatty-acid-CoA ligase |
| SLOPH_1334 | Lysyl-tRNA synthetase |
| SLOPH_2478 | MAK16 Nuclear protein |
| SLOPH_1339 | Manganese superoxide dismutase |
| SLOPH_2271 | Mannose-1-phosphate guanyltransferase |
| SLOPH_2315 | Mediator of RNA polymerase II transcription subunit 6 |
| SLOPH_1345 | Membrane protein with Yip1 domain protein, partial |
| SLOPH_2653 | metallopeptidase M24 |
| SLOPH_1347 | Methionine aminopeptidase |
| SLOPH_1348 | Methionyl-tRNA synthetase |
| SLOPH_1349 | Methyltransferase |
| SLOPH_2127 | methytransferase |
| SLOPH_1354 | Midasin, partial |
| SLOPH_1355 | Mismatch repair protein, partial |
| SLOPH_1358 | Mitochondrial-type Hsp40 |
| SLOPH_1362 | Mnd1 family protein |
| SLOPH_2430 | MOT2 transcriptional repressor |
| SLOPH_2294 | MRE11 meiotic recombination 11 protein A |
| SLOPH_1365 | mRNA capping enzyme |
| SLOPH_1366 | mRNA capping enzyme subunit alpha |
| SLOPH_1367 | mRNA-decapping enzyme 2 |
| SLOPH_2613 | MutSDNA mismatch repair protein, partial |
| SLOPH_1380 | N-acetyltransferase |
| SLOPH_1382 | N-myristoyltransferase |
| SLOPH_2405 | N2-N2-dimethylguanosine tRNA methyltransferase |
| SLOPH_1383 | N6 adenine specific DNA methylase |
| SLOPH_1386 | NAD-dependent glycerol-3-phosphate dehydrogenase |
| SLOPH_2657 | NADPH cytochrome P450 Reductase, partial |
| SLOPH_1390 | NLI interacting factor-like phosphatase |
| SLOPH_1393 | NOB1-domain containing protein |
| SLOPH_1394 | NOL1/NOP2/sun doamin containing protein, partial |
| SLOPH_1395 | Nonsense-mediated mRNA decay protein |
| SLOPH_1397 | Nonsense-mediated mRNA decay protein, partial |
| SLOPH_2672 | Nop2 protein, NOL1/NOP2/sun family protein, partial |
| SLOPH_2203 | NOP5 nucleolar protein |
| SLOPH_1401 | Nucleolar GTP-binding protein |
| SLOPH_1402 | Nucleolar GTP-binding protein 1 |
| SLOPH_1876 | nucleolar protein 10 like protein |
| SLOPH_1408 | Nucleolar protein nop5 |
| SLOPH_1404 | Nucleoside deaminase |
| SLOPH_1405 | Nucleoside diphosphate kinase |
| SLOPH_2191 | Nucleoside phosphatase |
| SLOPH_1409 | P-ATPase, partial |
| SLOPH_1410 | P-type ATPase |
| SLOPH_1413 | Peptidase family M16, partial |
| SLOPH_1414 | Peptidase M1 |
| SLOPH_2437 | Peptidyl-tRNA hydrolase type 2 |
| SLOPH_1420 | Per1-like domain protein |
| SLOPH_1426 | PHD-finger protein |
| SLOPH_1427 | Phenylalanyl-tRNA synthetase |
| SLOPH_1428 | Phenylalanyl-tRNA synthetase subunit alpha |
| SLOPH_1430 | Phosphatidate cytidylyltransferase |
| SLOPH_2332 | Phosphoacetylglucosamine mutase |
| SLOPH_1439 | Phosphoinositide 4-kinase |
| SLOPH_1441 | Phosphoinositide polyphosphatase (Sac family) |
| SLOPH_1446 | Phospholipid-transporting ATPase |
| SLOPH_1448 | Phosphomannomutase |
| SLOPH_1450 | Phosphotyrosyl phosphate activator |
| SLOPH_1451 | PI3/PI4 kinase |
| SLOPH_1452 | PIK-related kinase |
| SLOPH_1469 | Poly(A)-binding protein |
| SLOPH_1470 | Poly(A)+ RNA export protein |
| SLOPH_1471 | Polyadenylate binding proteim |
| SLOPH_1478 | pre-mRNA cleavage and polyadenylation factor Clp1 |
| SLOPH_2599 | Pre-mRNA splicing factor ATP-dependent RNA helicase |
| SLOPH_1482 | Pre-mRNA splicing factor RNA helicase |
| SLOPH_1487 | Proliferating cell nuclear antigen protein |
| SLOPH_1488 | Prolyl-tRNA synthetase |
| SLOPH_2674 | Proteasome B-type subunit |
| SLOPH_1489 | Proteasome subunit alpha type-3 |
| SLOPH_1490 | Proteasome subunit beta |
| SLOPH_1491 | Proteasome subunit beta type 2 |
| SLOPH_1492 | Protein disulfide isomerase |
| SLOPH_1493 | Protein involved in ER translocation |
| SLOPH_1494 | Protein involved in plasmid maintenance |
| SLOPH_1504 | Protein kinase |
| SLOPH_1513 | Protein phosphatase 2A |
| SLOPH_1514 | Protein phosphatase pp2A regulatory subunit B, partial |
| SLOPH_2249 | Pseudouridine synthase A |
| SLOPH_1522 | Pseudouridylate synthase |
| SLOPH_1524 | PUA domain containing protein |
| SLOPH_1526 | Pumilio protein |
| SLOPH_1483 | putative ATPases of PP-loop superfamily |
| SLOPH_1484 | putative cation-transporting ATPase |
| SLOPH_1528 | Putative GTP binding protein |
| SLOPH_454 | putative membrane protein |
| SLOPH_2258 | putative pelota protein |
| SLOPH_2287 | RAB GDP-dissociation inhibitor |
| SLOPH_1539 | RAN-specific GTPase activating protein |
| SLOPH_1540 | RAS GTPase |
| SLOPH_1542 | RAS-related GTP-binding protein Rab11 |
| SLOPH_1544 | Regulator of chromosome condensation |
| SLOPH_1547 | Replication factor C |
| SLOPH_1550 | Replication factor C subunit |
| SLOPH_1551 | Replication factor-A protein 1 |
| SLOPH_1553 | Retention in endoplasmic reticulum 1-like protein |
| SLOPH_1555 | Rhodanese-like domain protein |
| SLOPH_1559 | Ribonuclease HII |
| SLOPH_2433 | Ribonuclease Z |
| SLOPH_1562 | Ribonucleoside-diphosphate reductase small chain |
| SLOPH_1564 | Ribosomal protein L13A |
| SLOPH_1565 | Ribosomal protein L15 |
| SLOPH_2233 | Ribosomal protein L23 |
| SLOPH_1568 | Ribosomal protein L7A |
| SLOPH_1570 | Ribosomal protein S19 |
| SLOPH_1575 | Ribosomal RNA assembly protein |
| SLOPH_1581 | RIO kinase 1 |
| SLOPH_1585 | RNA polymerase II subunit 3 |
| SLOPH_1588 | RNA recognition motif protein |
| SLOPH_2157 | RNase L inhibitor of the ABC superfamily |
| SLOPH_2095 | RPN2 26S proteasome regulatory complex component |
| SLOPH_1598 | RuvB/Pontin protein, partial |
| SLOPH_1600 | S4-like ribosomal protein |
| SLOPH_1601 | S60 ribosomal protein L10 |
| SLOPH_1605 | SEC1 family transport protein SLY1, partial |
| SLOPH_1606 | SEC13 protein |
| SLOPH_1607 | SEC18-like vesicular fusion protein |
| SLOPH_2096 | SEC21 protein |
| SLOPH_1517 | SEC23 protein |
| SLOPH_1608 | SEC24-like protein |
| SLOPH_1609 | SEC31-like protein |
| SLOPH_2412 | Sec63-like protein |
| SLOPH_2427 | Septin |
| SLOPH_1614 | Septin |
| SLOPH_2331 | serine/threonine protein kinase |
| SLOPH_1627 | Serine/threonine protein kinase |
| SLOPH_2306 | Serine/Threonine protein kinase |
| SLOPH_1639 | Seryl-tRNA synthetase |
| SLOPH_1641 | SGT1-like protein |
| SLOPH_2432 | Shwachman-Bodian-Diamond syndrome like protein |
| SLOPH_1644 | Signal peptidase 18 kDa subunit |
| SLOPH_2152 | Signal peptidase protein |
| SLOPH_1647 | Signal recognition particle protein SRP54 |
| SLOPH_1648 | Signal recognition particle receptor alpha subunit, partial |
| SLOPH_1892 | SMC family protein, partial |
| SLOPH_1893 | SMC protein |
| SLOPH_1895 | SNF2 DNA helicase |
| SLOPH_1900 | SNF2 family N-terminal domain protein |
| SLOPH_1909 | structural maintenance of chromosome protein |
| SLOPH_1910 | Structure-specific recognition protein |
| SLOPH_2452 | SUA5 Putative translation factor |
| SLOPH_1911 | Subtilisin-like serine protease |
| SLOPH_2288 | Subunit of the transcription factor IIH complex |
| SLOPH_1919 | SWIB-domain-containing protein |
| SLOPH_1920 | SWIRM domain protein |
| SLOPH_1921 | Synaptobrevin protein |
| SLOPH_1923 | T complex protein 1 subunit beta |
| SLOPH_1924 | T-complex protein 1 |
| SLOPH_2334 | T-complex protein 1 epsilon subunit |
| SLOPH_1925 | T-complex protein 1 subunit alpha |
| SLOPH_1926 | T-complex protein 1 subunit delta |
| SLOPH_2739 | T-complex protein 1 subunit eta subunit |
| SLOPH_2418 | T-complex protein 1 zeta subunit |
| SLOPH_1932 | TAFII55 transcription factor family protein |
| SLOPH_1935 | TATA-binding protein-associated phosphoprotein |
| SLOPH_1936 | TatD related DNase |
| SLOPH_1942 | TFIIH transcription factor, partial |
| SLOPH_1943 | TGF-beta-inducible nuclear protein |
| SLOPH_1944 | Thioredoxin |
| SLOPH_2696 | Thymidylate kinase |
| SLOPH_1955 | TIP49 domain protein, partial |
| SLOPH_1957 | Transcription elongation complex subunit Cdc68 |
| SLOPH_2155 | Transcription elongation factor A SII |
| SLOPH_1958 | Transcription factor |
| SLOPH_2183 | Transcription factor Sfp1 |
| SLOPH_1961 | Transcription factor SPT5 |
| SLOPH_2327 | Transcription factor Tfb2 |
| SLOPH_2181 | Transcription factor TFIIA complex subunit Toa1 |
| SLOPH_1963 | Transcription initiation factor 4F subunit |
| SLOPH_2441 | Transcription initiation factor IIA gamma subunit |
| SLOPH_1964 | Transcription initiation factor IIB |
| SLOPH_2107 | Transcription initiation factor IIE alpha subunit |
| SLOPH_1966 | Transcription initiation factor TFIIB |
| SLOPH_1968 | Transcription initiation factor TFIID |
| SLOPH_2388 | Transcription initiation factor TFIID 150 kDa subunit |
| SLOPH_2444 | Transcription initiation factor TFIID 70 kDa subunit |
| SLOPH_1969 | Transcription initiation factor TFIID 90KDa |
| SLOPH_1971 | Transcription initiation factor TFIID subunit |
| SLOPH_2461 | Transcription initiation factor TFIID subunit 111 KDa |
| SLOPH_2118 | Transcription initiation factor TFIIIB Bdp1 subunit |
| SLOPH_1973 | Transcription initiation TFIID 10 |
| SLOPH_1979 | Translation elongation factor 1 alpha |
| SLOPH_1980 | Translation elongation factor 2 |
| SLOPH_1983 | Translation initiation factor eIF2 gamma subunit |
| SLOPH_1984 | Translation initiation factor IF-2b |
| SLOPH_1985 | Translation initiation factor IF-2P |
| SLOPH_1986 | Translocation protein SEC62 |
| SLOPH_1990 | Transmembrane amino acid transporter protein |
| SLOPH_1999 | tRNA (guanine-N7-)-methyltransferase |
| SLOPH_2000 | tRNA binding domain protein |
| SLOPH_2407 | tRNA nucleotidyltransferase-polyA polymerase, partial |
| SLOPH_2229 | tRNA pseudouridine synthase |
| SLOPH_2003 | tRNA pseudouridine synthase D, partial |
| SLOPH_2006 | Tryptophanyl-tRNA synthetase |
| SLOPH_2012 | Tyrosyl-tRNA synthetase |
| SLOPH_2020 | Ubiquitin C-terminal hydrolase ubp14, partial |
| SLOPH_2316 | Ubiquitin carboxyl-terminal hydrolase |
| SLOPH_2695 | ubiquitin family protein |
| SLOPH_2028 | Ubiquitin fusion degradation protein 1 |
| SLOPH_2185 | Ubiquitin-activating enzyme E1 |
| SLOPH_2034 | Ubiquitin-conjugating enzyme E2 |
| SLOPH_2035 | Ubiquitin-conjugating enzyme E2 2 |
| SLOPH_2702 | Ubiquitin-conjugating enzyme E2-16 kDa |
| SLOPH_2037 | Ubiquitin-conjugating enzyme E2G 1 |
| SLOPH_2038 | Ubiquitin-like activating enzyme, partial |
| SLOPH_2039 | ubiquitin/40s ribosomal protein S27a fusion |
| SLOPH_2041 | Undecaprenyl diphosphate synthase, partial |
| SLOPH_2046 | V-type ATPase subunit A |
| SLOPH_2047 | V-type ATPase subunit D |
| SLOPH_2160 | Vacuolar ATP synthase 95kDa Subunit |
| SLOPH_2050 | Vacuolar ATP synthase subunit B |
| SLOPH_2055 | Vacuolar ATP synthase subunit D |
| SLOPH_2059 | Valyl-tRNA synthetase |
| SLOPH_2063 | WD repeat and SOF domain-containing protein |
| SLOPH_2065 | WD repeat domain protein |
| SLOPH_2067 | WD repeat protein |
| SLOPH_2069 | WD repeat protein |
| SLOPH_2070 | WD repeat protein, partial |
| SLOPH_2217 | WD-40 repeat-containing protein |
| SLOPH_2077 | YEATS family protein |
| SLOPH_2082 | Zinc finger protein |
| SLOPH_2085 | Zinc finger protein |

*Spraguea lophii* proteins that are also found in *Saccharomyces cerevisiae*, *Nematocida parisii*, *Trachipleistophora hominis*, *Enterocytozoon bieneusi*, *Encephalitozoon cuniculi*, and *Nosema ceranae* (but not *Homo sapiens*):

| SLOPH_132 | apurinic/apyrimidinic endonuclease Apn1 |
| --- | --- |
| SLOPH_166 | Bromodomain protein |
| SLOPH_168 | Brr6-like protein |
| SLOPH_2343 | Ca2+-dependent lipid-binding protein |
| SLOPH_202 | Chitin synthase |
| SLOPH_214 | Cleavage and polyadenylation specificity factor |
| SLOPH_2266 | DNA primase large subunit |
| SLOPH_2296 | Exocyst complex subunit SEC6 |
| SLOPH_394 | Glutaminyl-tRNA synthetase |
| SLOPH_402 | Glycolipid 2-alpha-mannosyltransferase |
| SLOPH_446 | Homeodomain protein |
| SLOPH_1684 | hypothetical protein SLOPH 1684 |
| SLOPH_1817 | hypothetical protein SLOPH 1817 |
| SLOPH_1916 | hypothetical protein SLOPH 1916 |
| SLOPH_2600 | hypothetical protein SLOPH 2600 |
| SLOPH_1229 | Inorganic phosphate transporter PHO88 |
| SLOPH_1384 | Na+ H+ antiporter |
| SLOPH_1385 | Na+/H+ antiporter |
| SLOPH_2269 | Protein with TPR repeat, SEL1 subfamily |
| SLOPH_227 | Putative vacuolar transporter chaperone, partial |
| SLOPH_1915 | Sulfate transporter family protein |
| SLOPH_1883 | sulfate transporter protein |
| SLOPH_1962 | Transcription factor STE12 |
| SLOPH_2397 | VID27 cytoplasmic protein |
| SLOPH_1880 | Zinc finger C2H2 protein |

*Spraguea lophii* proteins that are also found in *Nematocida parisii*, *Trachipleistophora hominis*, *Enterocytozoon bieneusi*, *Encephalitozoon cuniculi*, and *Nosema ceranae* (but not *Homo sapiens* or *Saccharomyces cerevisiae*):

| SLOPH_16 | 26S Proteasome core subunit-alpha-6 |
| --- | --- |
| SLOPH_23 | 26S proteasome regulatory subunit S5A |
| SLOPH_24 | 26S proteosome subunit alpha-4 |
| SLOPH_83 | AAA ATPase |
| SLOPH_2213 | actin-depolymerizing factor |
| SLOPH_113 | ADP/ATP carrier protein |
| SLOPH_114 | ADP/ATP carrier protein |
| SLOPH_115 | ADP/ATP carrier protein |
| SLOPH_116 | ADP/ATP carrier protein |
| SLOPH_117 | ADP/ATP carrier protein |
| SLOPH_130 | Ankyrin repeat containing protein |
| SLOPH_2218 | Aquaporin-like protein |
| SLOPH_157 | B-box zinc finger protein |
| SLOPH_2634 | Baculovirus inhibitor of apoptosis like protein |
| SLOPH_176 | Calmodulin-dependent protein kinase |
| SLOPH_199 | Cell division protein kinase |
| SLOPH_218 | Coatomer subunit delta subunit |
| SLOPH_253 | Cytidylate kinase |
| SLOPH_258 | DBF zinc finger domain protein |
| SLOPH_278 | Diphthamide synthesis protein |
| SLOPH_335 | Emp24/gp25L/p24 family protein |
| SLOPH_339 | Endoplasmic Reticulum Oxidoreductin 1 |
| SLOPH_378 | G2/mitotic specific cyclin 1 |
| SLOPH_411 | GTP-binding protein |
| SLOPH_416 | Heat shock protein 70 |
| SLOPH_426 | Histone deacetylase 3 |
| SLOPH_429 | Histone H2b |
| SLOPH_1139 | hypothetical protein SLOPH 1139, partial |
| SLOPH_1205 | hypothetical protein SLOPH 1205 |
| SLOPH_1212 | hypothetical protein SLOPH 1212 |
| SLOPH_1213 | hypothetical protein SLOPH 1213 |
| SLOPH_1650 | hypothetical protein SLOPH 1650 |
| SLOPH_1653 | hypothetical protein SLOPH 1653 |
| SLOPH_1658 | hypothetical protein SLOPH 1658, partial |
| SLOPH_1678 | hypothetical protein SLOPH 1678 |
| SLOPH_1679 | hypothetical protein SLOPH 1679 |
| SLOPH_1691 | hypothetical protein SLOPH 1691 |
| SLOPH_1696 | hypothetical protein SLOPH 1696 |
| SLOPH_1706 | hypothetical protein SLOPH 1706 |
| SLOPH_1708 | hypothetical protein SLOPH 1708 |
| SLOPH_1714 | hypothetical protein SLOPH 1714 |
| SLOPH_1715 | hypothetical protein SLOPH 1715 |
| SLOPH_1736 | hypothetical protein SLOPH 1736 |
| SLOPH_1757 | hypothetical protein SLOPH 1757 |
| SLOPH_1776 | hypothetical protein SLOPH 1776 |
| SLOPH_1779 | hypothetical protein SLOPH 1779 |
| SLOPH_1784 | hypothetical protein SLOPH 1784 |
| SLOPH_1803 | hypothetical protein SLOPH 1803 |
| SLOPH_1819 | hypothetical protein SLOPH 1819 |
| SLOPH_1829 | hypothetical protein SLOPH 1829 |
| SLOPH_1837 | hypothetical protein SLOPH 1837, partial |
| SLOPH_1839 | hypothetical protein SLOPH 1839 |
| SLOPH_1857 | hypothetical protein SLOPH 1857 |
| SLOPH_1881 | hypothetical protein SLOPH 1881 |
| SLOPH_2106 | hypothetical protein SLOPH 2106 |
| SLOPH_2216 | hypothetical protein SLOPH 2216 |
| SLOPH_2238 | hypothetical protein SLOPH 2238 |
| SLOPH_2239 | hypothetical protein SLOPH 2239 |
| SLOPH_2264 | hypothetical protein SLOPH 2264 |
| SLOPH_2317 | hypothetical protein SLOPH 2317 |
| SLOPH_2325 | hypothetical protein SLOPH 2325 |
| SLOPH_2337 | hypothetical protein SLOPH 2337 |
| SLOPH_2351 | hypothetical protein SLOPH 2351 |
| SLOPH_2354 | hypothetical protein SLOPH 2354 |
| SLOPH_2382 | hypothetical protein SLOPH 2382 |
| SLOPH_2718 | hypothetical protein SLOPH 2718 |
| SLOPH_540 | hypothetical protein SLOPH 540 |
| SLOPH_849 | hypothetical protein SLOPH 849, partial |
| SLOPH_864 | hypothetical protein SLOPH 864 |
| SLOPH_873 | hypothetical protein SLOPH 873 |
| SLOPH_919 | hypothetical protein SLOPH 919 |
| SLOPH_1244 | Kinetochore protein nuf2 |
| SLOPH_1327 | Lipase class 3 protein |
| SLOPH_2372 | mRNA capping enzyme beta chain protein |
| SLOPH_1398 | Not1 negative regulator of transcription, partial |
| SLOPH_1399 | NOT2/NOT3/NOT5 family protein |
| SLOPH_2747 | Nuclear pore complex protein Nup155, partial |
| SLOPH_1403 | Nucleoporin Nic96 |
| SLOPH_2406 | origin recognition complex subunit 2 |
| SLOPH_2201 | Permease of the major facilitator superfamily |
| SLOPH_1537 | RAD4 protein, partial |
| SLOPH_1545 | Replication factor A protein 2, partial |
| SLOPH_1887 | Sld5 domain protein |
| SLOPH_1894 | SNARE domain containing protein |
| SLOPH_1927 | T-complex protein 1 subunit theta |
| SLOPH_1929 | T-SNARE complex subunit/syntaxin |
| SLOPH_1930 | t-SNARE complex subunit/syntaxin |
| SLOPH_1931 | t-SNARE complex subunit/syntaxin |
| SLOPH_1941 | Tetratricopeptide repeat containing protein |
| SLOPH_2350 | Thioredoxin |
| SLOPH_1948 | Threonyl-tRNA synthetase |
| SLOPH_2451 | Transcription initiation factor IIE beta subunit |
| SLOPH_2390 | Transcription initiation factor TFIID component TAF4 family protein |
| SLOPH_2697 | Translation elongation factor EF2 |
| SLOPH_2429 | Vesicle coat complex COPII Sec23/Sec24 |
| SLOPH_2062 | WD domain containing protein |
| SLOPH_2071 | WD repeat-containing protein |
| SLOPH_2073 | WD40 repeat protein |
| SLOPH_2079 | YL1 nuclear domain protein |
| SLOPH_2682 | Zinc finger C3HC4 type protein |
| SLOPH_2711 | Zinc finger protein, partial |
| SLOPH_2090 | ZIP Zinc transporter |
| SLOPH_2092 | Zn-dependent protease |

*Spraguea lophii* proteins that are also found in *Trachipleistophora hominis,* *Enterocytozoon bieneusi*, *Encephalitozoon cuniculi*, *Nosema ceranae,* (but not *Nematocida parisii*, *Homo sapiens* or *Saccharomyces cerevisiae*):

| SLOPH_63 | 60S ribosomal protein L22 |
| --- | --- |
| SLOPH_152 | ATP-dependent RNA helicase |
| SLOPH_156 | ATPase of the AAA+ family |
| SLOPH_351 | Exocyst complex component Sec10 |
| SLOPH_423 | Histidine acid phosphatase |
| SLOPH_1906 | Hsp 40 |
| SLOPH_1202 | hypothetical protein SLOPH 1202 |
| SLOPH_1204 | hypothetical protein SLOPH 1204 |
| SLOPH_1208 | hypothetical protein SLOPH 1208 |
| SLOPH_1216 | hypothetical protein SLOPH 1216, partial |
| SLOPH_1218 | hypothetical protein SLOPH 1218 |
| SLOPH_1649 | hypothetical protein SLOPH 1649 |
| SLOPH_1664 | hypothetical protein SLOPH 1664 |
| SLOPH_1667 | hypothetical protein SLOPH 1667 |
| SLOPH_1685 | hypothetical protein SLOPH 1685 |
| SLOPH_1700 | hypothetical protein SLOPH 1700 |
| SLOPH_1704 | hypothetical protein SLOPH 1704 |
| SLOPH_1749 | hypothetical protein SLOPH 1749 |
| SLOPH_1756 | hypothetical protein SLOPH 1756 |
| SLOPH_1773 | hypothetical protein SLOPH 1773 |
| SLOPH_1775 | hypothetical protein SLOPH 1775 |
| SLOPH_1796 | hypothetical protein SLOPH 1796 |
| SLOPH_1804 | hypothetical protein SLOPH 1804 |
| SLOPH_1805 | hypothetical protein SLOPH 1805 |
| SLOPH_1807 | hypothetical protein SLOPH 1807 |
| SLOPH_1809 | hypothetical protein SLOPH 1809 |
| SLOPH_1820 | hypothetical protein SLOPH 1820 |
| SLOPH_1834 | hypothetical protein SLOPH 1834 |
| SLOPH_1841 | hypothetical protein SLOPH 1841 |
| SLOPH_1854 | hypothetical protein SLOPH 1854 |
| SLOPH_1870 | hypothetical protein SLOPH 1870 |
| SLOPH_1885 | hypothetical protein SLOPH 1885 |
| SLOPH_2109 | hypothetical protein SLOPH 2109 |
| SLOPH_2173 | hypothetical protein SLOPH 2173 |
| SLOPH_2206 | hypothetical protein SLOPH 2206 |
| SLOPH_2279 | hypothetical protein SLOPH 2279 |
| SLOPH_2344 | hypothetical protein SLOPH 2344 |
| SLOPH_2349 | hypothetical protein SLOPH 2349 |
| SLOPH_2368 | hypothetical protein SLOPH 2368 |
| SLOPH_2403 | hypothetical protein SLOPH 2403 |
| SLOPH_2510 | hypothetical protein SLOPH 2510, partial |
| SLOPH_1368 | MscS-Like mechanosensitive ion channel |
| SLOPH_2335 | Myb-like DNA-binding domain containing protein |
| SLOPH_1407 | Nucleosome assembly protein |
| SLOPH_2725 | putative Brix domain protein, partial |
| SLOPH_1536 | Rab-GDP dissociation inhibitor |
| SLOPH_1554 | Rho-type GTPase |
| SLOPH_1556 | RhoGAP domain containing protein |
| SLOPH_1631 | Serine/threonine protein phosphatase |
| SLOPH_1884 | Spore wall protein 12 like protein |
| SLOPH_1959 | transcription factor E2F |
| SLOPH_2004 | tRNA-dihydrouridine synthase A, partial |
| SLOPH_2019 | Ubiquitin |
| SLOPH_2380 | Vacuolar sorting protein 9 domain protein |
| SLOPH_2061 | Vesicular transport protein, partial |
| SLOPH_1877 | WD40 domain-containing protein |

*Spraguea lophii* proteins that are also found in *Trachipleistophora hominis* (but not *Enterocytozoon bieneusi*, *Encephalitozoon cuniculi*, *Nosema ceranae,* *Nematocida parisii*, *Homo sapiens* or *Saccharomyces cerevisiae*):

| SLOPH_106 | Adenosine kinase |
| --- | --- |
| SLOPH_105 | Adenosine kinase |
| SLOPH_2328 | ADP-ribosylation factor |
| SLOPH_138 | Arrestin |
| SLOPH_219 | Cold-shock DNA-binding protein |
| SLOPH_287 | DNA mismatch repair protein MutL, partial |
| SLOPH_2716 | dsDNA binding protein |
| SLOPH_2166 | Forkhead protein |
| SLOPH_2438 | GTP-binding protein rho1 |
| SLOPH_418 | Heat shock protein 90 |
| SLOPH_1019 | hypothetical protein SLOPH 1019 |
| SLOPH_1032 | hypothetical protein SLOPH 1032 |
| SLOPH_1038 | hypothetical protein SLOPH 1038 |
| SLOPH_1052 | hypothetical protein SLOPH 1052 |
| SLOPH_1112 | hypothetical protein SLOPH 1112 |
| SLOPH_1114 | hypothetical protein SLOPH 1114 |
| SLOPH_1120 | hypothetical protein SLOPH 1120, partial |
| SLOPH_1126 | hypothetical protein SLOPH 1126 |
| SLOPH_1167 | hypothetical protein SLOPH 1167 |
| SLOPH_1189 | hypothetical protein SLOPH 1189 |
| SLOPH_1660 | hypothetical protein SLOPH 1660, partial |
| SLOPH_1681 | hypothetical protein SLOPH 1681 |
| SLOPH_1695 | hypothetical protein SLOPH 1695 |
| SLOPH_1717 | hypothetical protein SLOPH 1717 |
| SLOPH_1735 | hypothetical protein SLOPH 1735, partial |
| SLOPH_1766 | hypothetical protein SLOPH 1766 |
| SLOPH_1815 | hypothetical protein SLOPH 1815 |
| SLOPH_1836 | hypothetical protein SLOPH 1836 |
| SLOPH_1855 | hypothetical protein SLOPH 1855 |
| SLOPH_2197 | hypothetical protein SLOPH 2197 |
| SLOPH_236 | hypothetical protein SLOPH 236 |
| SLOPH_2468 | hypothetical protein SLOPH 2468 |
| SLOPH_2717 | hypothetical protein SLOPH 2717 |
| SLOPH_2732 | hypothetical protein SLOPH 2732 |
| SLOPH_2733 | hypothetical protein SLOPH 2733 |
| SLOPH_2734 | hypothetical protein SLOPH 2734 |
| SLOPH_484 | hypothetical protein SLOPH 484 |
| SLOPH_488 | hypothetical protein SLOPH 488 |
| SLOPH_495 | hypothetical protein SLOPH 495 |
| SLOPH_504 | hypothetical protein SLOPH 504 |
| SLOPH_509 | hypothetical protein SLOPH 509, partial |
| SLOPH_515 | hypothetical protein SLOPH 515, partial |
| SLOPH_536 | hypothetical protein SLOPH 536 |
| SLOPH_544 | hypothetical protein SLOPH 544, partial |
| SLOPH_608 | hypothetical protein SLOPH 608, partial |
| SLOPH_629 | hypothetical protein SLOPH 629 |
| SLOPH_649 | hypothetical protein SLOPH 649, partial |
| SLOPH_655 | hypothetical protein SLOPH 655 |
| SLOPH_685 | hypothetical protein SLOPH 685 |
| SLOPH_694 | hypothetical protein SLOPH 694 |
| SLOPH_709 | hypothetical protein SLOPH 709 |
| SLOPH_710 | hypothetical protein SLOPH 710 |
| SLOPH_722 | hypothetical protein SLOPH 722 |
| SLOPH_766 | hypothetical protein SLOPH 766, partial |
| SLOPH_800 | hypothetical protein SLOPH 800 |
| SLOPH_806 | hypothetical protein SLOPH 806, partial |
| SLOPH_809 | hypothetical protein SLOPH 809 |
| SLOPH_813 | hypothetical protein SLOPH 813 |
| SLOPH_831 | hypothetical protein SLOPH 831 |
| SLOPH_835 | hypothetical protein SLOPH 835 |
| SLOPH_866 | hypothetical protein SLOPH 866 |
| SLOPH_885 | hypothetical protein SLOPH 885 |
| SLOPH_898 | hypothetical protein SLOPH 898 |
| SLOPH_959 | hypothetical protein SLOPH 959 |
| SLOPH_968 | hypothetical protein SLOPH 968 |
| SLOPH_1445 | Phospholipid-translocating P-type ATPase, partial |
| SLOPH_1466 | polar tube protein PTP2 |
| SLOPH_1515 | Protein phosphatase regulatory subunit A, partial |
| SLOPH_1558 | RhoGEF domain protein, partial |
| SLOPH_2253 | Ser/Thr protein phosphatase family protein |
| SLOPH_2740 | Transcription factor MBF1 |
| SLOPH_2084 | Zinc finger protein |

*Spraguea lophii* proteins that are also found in *Trachipleistophora hominis, Homo sapiens* and *Saccharomyces cerevisiae* (but not *Enterocytozoon bieneusi, Encephalitozoon cuniculi*, *Nosema ceranae* or *Nematocida parisii*):

| SLOPH_1444 | Phospholipase/carboxylesterase family protein |
| --- | --- |
| SLOPH_1416 | Peptidase M18 |
| SLOPH_131 | Apolipoprotein A-I binding protein |
| SLOPH_139 | Arsenite-transporting ATPase |
| SLOPH_2625 | HORMA domain-containing protein, partial |
| SLOPH_207 | Choline/ethanolamine kinase |
| SLOPH_239 | hypothetical protein SLOPH 239, partial |
| SLOPH_1454 | Plasma membrane calcium-transporting ATPase, partial |
| SLOPH_2602 | Cation-transporting atpase, partial |
| SLOPH_2601 | Cation-transporting atpase plant, partial |
| SLOPH_2595 | clathrin-associated AP-2 complex component |
| SLOPH_343 | Eukaryotic membrane protein family |
| SLOPH_601 | hypothetical protein SLOPH 601 |

*Spraguea lophii* proteins that are also found in *Homo sapiens* and *Saccharomyces cerevisiae* (but not *Enterocytozoon bieneusi*, *Trachipleistophora hominis, Encephalitozoon cuniculi*, *Nosema ceranae* or *Nematocida parisii*):

| SLOPH_441 | Homeobox domain protein |
| --- | --- |
| SLOPH_1351 | Methyltransferase |
| SLOPH_2022 | Ubiquitin carboxyl-terminal hydrolase |
| SLOPH_2162 | RNA binding protein |
| SLOPH_178 | Calponin domain protein |
| SLOPH_2675 | Radical S-adenosyl methionine and flavodoxin domain protein |
| SLOPH_165 | Bromodomain containing protein, partial |

*Spraguea lophii* proteins that form OrthoMCL clusters but that are not found in *Homo sapiens* and *Saccharomyces cerevisiae*, *Enterocytozoon bieneusi*, *Encephalitozoon cuniculi*, *Nosema ceranae* or *Nematocida parisii*):

| SLOPH_2312 | Amino acid permease, partial |
| --- | --- |
| SLOPH_125 | Amino acid transporter |
| SLOPH_1003 | hypothetical protein SLOPH 1003 |
| SLOPH_1012 | hypothetical protein SLOPH 1012, partial |
| SLOPH_1013 | hypothetical protein SLOPH 1013 |
| SLOPH_1014 | hypothetical protein SLOPH 1014 |
| SLOPH_1023 | hypothetical protein SLOPH 1023 |
| SLOPH_1024 | hypothetical protein SLOPH 1024 |
| SLOPH_1044 | hypothetical protein SLOPH 1044 |
| SLOPH_1047 | hypothetical protein SLOPH 1047 |
| SLOPH_1048 | hypothetical protein SLOPH 1048 |
| SLOPH_1056 | hypothetical protein SLOPH 1056, partial |
| SLOPH_1061 | hypothetical protein SLOPH 1061 |
| SLOPH_1062 | hypothetical protein SLOPH 1062 |
| SLOPH_1064 | hypothetical protein SLOPH 1064, partial |
| SLOPH_1073 | hypothetical protein SLOPH 1073 |
| SLOPH_1079 | hypothetical protein SLOPH 1079, partial |
| SLOPH_1080 | hypothetical protein SLOPH 1080 |
| SLOPH_1085 | hypothetical protein SLOPH 1085 |
| SLOPH_1087 | hypothetical protein SLOPH 1087 |
| SLOPH_1091 | hypothetical protein SLOPH 1091 |
| SLOPH_1094 | hypothetical protein SLOPH 1094 |
| SLOPH_1106 | hypothetical protein SLOPH 1106 |
| SLOPH_1108 | hypothetical protein SLOPH 1108 |
| SLOPH_1116 | hypothetical protein SLOPH 1116, partial |
| SLOPH_1121 | hypothetical protein SLOPH 1121 |
| SLOPH_1122 | hypothetical protein SLOPH 1122 |
| SLOPH_1151 | hypothetical protein SLOPH 1151, partial |
| SLOPH_1152 | hypothetical protein SLOPH 1152 |
| SLOPH_1160 | hypothetical protein SLOPH 1160 |
| SLOPH_1161 | hypothetical protein SLOPH 1161 |
| SLOPH_1165 | hypothetical protein SLOPH 1165 |
| SLOPH_1178 | hypothetical protein SLOPH 1178 |
| SLOPH_1191 | hypothetical protein SLOPH 1191 |
| SLOPH_1209 | hypothetical protein SLOPH 1209 |
| SLOPH_1210 | hypothetical protein SLOPH 1210, partial |
| SLOPH_1220 | hypothetical protein SLOPH 1220, partial |
| SLOPH_1226 | hypothetical protein SLOPH 1226 |
| SLOPH_1720 | hypothetical protein SLOPH 1720 |
| SLOPH_1844 | hypothetical protein SLOPH 1844, partial |
| SLOPH_2149 | hypothetical protein SLOPH 2149 |
| SLOPH_225 | hypothetical protein SLOPH 225 |
| SLOPH_229 | hypothetical protein SLOPH 229 |
| SLOPH_231 | hypothetical protein SLOPH 231 |
| SLOPH_2323 | hypothetical protein SLOPH 2323 |
| SLOPH_2421 | hypothetical protein SLOPH 2421 |
| SLOPH_2422 | hypothetical protein SLOPH 2422 |
| SLOPH_2446 | hypothetical protein SLOPH 2446 |
| SLOPH_2447 | hypothetical protein SLOPH 2447 |
| SLOPH_2458 | hypothetical protein SLOPH 2458 |
| SLOPH_2482 | hypothetical protein SLOPH 2482 |
| SLOPH_2485 | hypothetical protein SLOPH 2485, partial |
| SLOPH_2486 | hypothetical protein SLOPH 2486, partial |
| SLOPH_2487 | hypothetical protein SLOPH 2487, partial |
| SLOPH_2488 | hypothetical protein SLOPH 2488, partial |
| SLOPH_2493 | hypothetical protein SLOPH 2493, partial |
| SLOPH_2497 | hypothetical protein SLOPH 2497, partial |
| SLOPH_2499 | hypothetical protein SLOPH 2499, partial |
| SLOPH_2503 | hypothetical protein SLOPH 2503, partial |
| SLOPH_2504 | hypothetical protein SLOPH 2504, partial |
| SLOPH_2505 | hypothetical protein SLOPH 2505, partial |
| SLOPH_2506 | hypothetical protein SLOPH 2506, partial |
| SLOPH_2507 | hypothetical protein SLOPH 2507 |
| SLOPH_2508 | hypothetical protein SLOPH 2508, partial |
| SLOPH_2509 | hypothetical protein SLOPH 2509, partial |
| SLOPH_2512 | hypothetical protein SLOPH 2512, partial |
| SLOPH_2513 | hypothetical protein SLOPH 2513, partial |
| SLOPH_2515 | hypothetical protein SLOPH 2515, partial |
| SLOPH_2517 | hypothetical protein SLOPH 2517 |
| SLOPH_2518 | hypothetical protein SLOPH 2518 |
| SLOPH_2524 | hypothetical protein SLOPH 2524, partial |
| SLOPH_2526 | hypothetical protein SLOPH 2526, partial |
| SLOPH_2527 | hypothetical protein SLOPH 2527 |
| SLOPH_2532 | hypothetical protein SLOPH 2532, partial |
| SLOPH_2533 | hypothetical protein SLOPH 2533, partial |
| SLOPH_2534 | hypothetical protein SLOPH 2534 |
| SLOPH_2536 | hypothetical protein SLOPH 2536, partial |
| SLOPH_2538 | hypothetical protein SLOPH 2538, partial |
| SLOPH_2544 | hypothetical protein SLOPH 2544, partial |
| SLOPH_2545 | hypothetical protein SLOPH 2545, partial |
| SLOPH_2546 | hypothetical protein SLOPH 2546 |
| SLOPH_2547 | hypothetical protein SLOPH 2547, partial |
| SLOPH_2548 | hypothetical protein SLOPH 2548 |
| SLOPH_2549 | hypothetical protein SLOPH 2549, partial |
| SLOPH_2552 | hypothetical protein SLOPH 2552, partial |
| SLOPH_2553 | hypothetical protein SLOPH 2553, partial |
| SLOPH_2554 | hypothetical protein SLOPH 2554, partial |
| SLOPH_2557 | hypothetical protein SLOPH 2557 |
| SLOPH_2565 | hypothetical protein SLOPH 2565, partial |
| SLOPH_2571 | hypothetical protein SLOPH 2571, partial |
| SLOPH_2577 | hypothetical protein SLOPH 2577, partial |
| SLOPH_2579 | hypothetical protein SLOPH 2579, partial |
| SLOPH_2581 | hypothetical protein SLOPH 2581, partial |
| SLOPH_2587 | hypothetical protein SLOPH 2587, partial |
| SLOPH_2588 | hypothetical protein SLOPH 2588 |
| SLOPH_2589 | hypothetical protein SLOPH 2589 |
| SLOPH_2604 | hypothetical protein SLOPH 2604 |
| SLOPH_2636 | hypothetical protein SLOPH 2636 |
| SLOPH_2661 | hypothetical protein SLOPH 2661 |
| SLOPH_2667 | hypothetical protein SLOPH 2667, partial |
| SLOPH_2698 | hypothetical protein SLOPH 2698, partial |
| SLOPH_2706 | hypothetical protein SLOPH 2706, partial |
| SLOPH_2746 | hypothetical protein SLOPH 2746 |
| SLOPH_459 | hypothetical protein SLOPH 459 |
| SLOPH_466 | hypothetical protein SLOPH 466 |
| SLOPH_467 | hypothetical protein SLOPH 467 |
| SLOPH_471 | hypothetical protein SLOPH 471 |
| SLOPH_482 | hypothetical protein SLOPH 482 |
| SLOPH_496 | hypothetical protein SLOPH 496 |
| SLOPH_505 | hypothetical protein SLOPH 505, partial |
| SLOPH_507 | hypothetical protein SLOPH 507 |
| SLOPH_512 | hypothetical protein SLOPH 512 |
| SLOPH_527 | hypothetical protein SLOPH 527 |
| SLOPH_529 | hypothetical protein SLOPH 529 |
| SLOPH_556 | hypothetical protein SLOPH 556 |
| SLOPH_558 | hypothetical protein SLOPH 558, partial |
| SLOPH_559 | hypothetical protein SLOPH 559 |
| SLOPH_561 | hypothetical protein SLOPH 561, partial |
| SLOPH_562 | hypothetical protein SLOPH 562 |
| SLOPH_563 | hypothetical protein SLOPH 563, partial |
| SLOPH_571 | hypothetical protein SLOPH 571 |
| SLOPH_575 | hypothetical protein SLOPH 575 |
| SLOPH_576 | hypothetical protein SLOPH 576 |
| SLOPH_580 | hypothetical protein SLOPH 580 |
| SLOPH_586 | hypothetical protein SLOPH 586 |
| SLOPH_588 | hypothetical protein SLOPH 588 |
| SLOPH_600 | hypothetical protein SLOPH 600 |
| SLOPH_603 | hypothetical protein SLOPH 603 |
| SLOPH_607 | hypothetical protein SLOPH 607 |
| SLOPH_612 | hypothetical protein SLOPH 612 |
| SLOPH_614 | hypothetical protein SLOPH 614, partial |
| SLOPH_625 | hypothetical protein SLOPH 625 |
| SLOPH_632 | hypothetical protein SLOPH 632 |
| SLOPH_634 | hypothetical protein SLOPH 634 |
| SLOPH_635 | hypothetical protein SLOPH 635 |
| SLOPH_638 | hypothetical protein SLOPH 638 |
| SLOPH_643 | hypothetical protein SLOPH 643, partial |
| SLOPH_646 | hypothetical protein SLOPH 646, partial |
| SLOPH_652 | hypothetical protein SLOPH 652 |
| SLOPH_656 | hypothetical protein SLOPH 656 |
| SLOPH_658 | hypothetical protein SLOPH 658 |
| SLOPH_670 | hypothetical protein SLOPH 670 |
| SLOPH_672 | hypothetical protein SLOPH 672 |
| SLOPH_679 | hypothetical protein SLOPH 679, partial |
| SLOPH_680 | hypothetical protein SLOPH 680 |
| SLOPH_681 | hypothetical protein SLOPH 681 |
| SLOPH_682 | hypothetical protein SLOPH 682 |
| SLOPH_696 | hypothetical protein SLOPH 696 |
| SLOPH_697 | hypothetical protein SLOPH 697 |
| SLOPH_698 | hypothetical protein SLOPH 698 |
| SLOPH_701 | hypothetical protein SLOPH 701 |
| SLOPH_705 | hypothetical protein SLOPH 705, partial |
| SLOPH_718 | hypothetical protein SLOPH 718, partial |
| SLOPH_725 | hypothetical protein SLOPH 725 |
| SLOPH_726 | hypothetical protein SLOPH 726 |
| SLOPH_731 | hypothetical protein SLOPH 731 |
| SLOPH_733 | hypothetical protein SLOPH 733, partial |
| SLOPH_737 | hypothetical protein SLOPH 737 |
| SLOPH_745 | hypothetical protein SLOPH 745 |
| SLOPH_746 | hypothetical protein SLOPH 746 |
| SLOPH_748 | hypothetical protein SLOPH 748, partial |
| SLOPH_754 | hypothetical protein SLOPH 754 |
| SLOPH_757 | hypothetical protein SLOPH 757 |
| SLOPH_780 | hypothetical protein SLOPH 780 |
| SLOPH_781 | hypothetical protein SLOPH 781 |
| SLOPH_782 | hypothetical protein SLOPH 782 |
| SLOPH_785 | hypothetical protein SLOPH 785 |
| SLOPH_786 | hypothetical protein SLOPH 786, partial |
| SLOPH_787 | hypothetical protein SLOPH 787 |
| SLOPH_792 | hypothetical protein SLOPH 792 |
| SLOPH_797 | hypothetical protein SLOPH 797 |
| SLOPH_799 | hypothetical protein SLOPH 799 |
| SLOPH_808 | hypothetical protein SLOPH 808 |
| SLOPH_810 | hypothetical protein SLOPH 810 |
| SLOPH_818 | hypothetical protein SLOPH 818 |
| SLOPH_827 | hypothetical protein SLOPH 827 |
| SLOPH_830 | hypothetical protein SLOPH 830, partial |
| SLOPH_858 | hypothetical protein SLOPH 858, partial |
| SLOPH_859 | hypothetical protein SLOPH 859 |
| SLOPH_860 | hypothetical protein SLOPH 860 |
| SLOPH_863 | hypothetical protein SLOPH 863 |
| SLOPH_875 | hypothetical protein SLOPH 875 |
| SLOPH_880 | hypothetical protein SLOPH 880 |
| SLOPH_894 | hypothetical protein SLOPH 894 |
| SLOPH_895 | hypothetical protein SLOPH 895 |
| SLOPH_899 | hypothetical protein SLOPH 899, partial |
| SLOPH_907 | hypothetical protein SLOPH 907 |
| SLOPH_926 | hypothetical protein SLOPH 926 |
| SLOPH_931 | hypothetical protein SLOPH 931, partial |
| SLOPH_943 | hypothetical protein SLOPH 943 |
| SLOPH_948 | hypothetical protein SLOPH 948 |
| SLOPH_949 | hypothetical protein SLOPH 949 |
| SLOPH_951 | hypothetical protein SLOPH 951 |
| SLOPH_952 | hypothetical protein SLOPH 952 |
| SLOPH_953 | hypothetical protein SLOPH 953 |
| SLOPH_954 | hypothetical protein SLOPH 954 |
| SLOPH_956 | hypothetical protein SLOPH 956 |
| SLOPH_961 | hypothetical protein SLOPH 961 |
| SLOPH_962 | hypothetical protein SLOPH 962 |
| SLOPH_963 | hypothetical protein SLOPH 963 |
| SLOPH_964 | hypothetical protein SLOPH 964 |
| SLOPH_965 | hypothetical protein SLOPH 965 |
| SLOPH_966 | hypothetical protein SLOPH 966 |
| SLOPH_974 | hypothetical protein SLOPH 974 |
| SLOPH_981 | hypothetical protein SLOPH 981, partial |
| SLOPH_982 | hypothetical protein SLOPH 982 |
| SLOPH_983 | hypothetical protein SLOPH 983, partial |
| SLOPH_986 | hypothetical protein SLOPH 986 |
| SLOPH_987 | hypothetical protein SLOPH 987, partial |
| SLOPH_988 | hypothetical protein SLOPH 988, partial |
| SLOPH_991 | hypothetical protein SLOPH 991 |
| SLOPH_935 | hypothetical protein SLOPH_935, partial |
| SLOPH_1278 | Leucine rich repeat protein |
| SLOPH_1279 | Leucine rich repeat protein |
| SLOPH_2648 | Leucine rich repeat protein |
| SLOPH_1258 | Leucine rich repeat protein |
| SLOPH_2243 | Leucine rich repeat protein |
| SLOPH_1265 | Leucine rich repeat protein |
| SLOPH_1266 | Leucine rich repeat protein |
| SLOPH_1273 | Leucine rich repeat protein |
| SLOPH_1325 | Leucine rich repeat protein |
| SLOPH_1290 | Leucine rich repeat protein |
| SLOPH_1293 | Leucine rich repeat protein |
| SLOPH_1295 | Leucine rich repeat protein |
| SLOPH_1297 | Leucine rich repeat protein |
| SLOPH_1298 | Leucine rich repeat protein |
| SLOPH_1303 | Leucine rich repeat protein |
| SLOPH_1306 | Leucine rich repeat protein |
| SLOPH_2169 | Leucine rich repeat protein |
| SLOPH_1312 | Leucine rich repeat protein |
| SLOPH_1320 | Leucine rich repeat protein |
| SLOPH_1317 | Leucine rich repeat protein |
| SLOPH_1254 | Leucine rich repeat protein |
| SLOPH_1255 | Leucine rich repeat protein |
| SLOPH_2450 | Leucine rich repeat protein |
| SLOPH_2423 | Leucine rich repeat protein |
| SLOPH_2464 | Leucine rich repeat protein |
| SLOPH_1323 | Leucine rich repeat protein |
| SLOPH_2290 | Leucine rich repeat protein |
| SLOPH_2360 | Leucine rich repeat protein |
| SLOPH_1260 | Leucine rich repeat protein |
| SLOPH_2619 | Leucine rich repeat protein |
| SLOPH_1261 | Leucine rich repeat protein |
| SLOPH_1248 | Leucine rich repeat protein |
| SLOPH_1263 | Leucine rich repeat protein |
| SLOPH_1321 | Leucine rich repeat protein |
| SLOPH_226 | Leucine rich repeat protein |
| SLOPH_1271 | Leucine rich repeat protein |
| SLOPH_2467 | Leucine rich repeat protein |
| SLOPH_1287 | Leucine rich repeat protein |
| SLOPH_1289 | Leucine rich repeat protein |
| SLOPH_1291 | Leucine rich repeat protein |
| SLOPH_1270 | Leucine rich repeat protein, partial |
| SLOPH_2649 | Leucine rich repeat protein, partial |
| SLOPH_1296 | Leucine rich repeat protein, partial |
| SLOPH_2609 | Leucine rich repeat protein, partial |
| SLOPH_2646 | Leucine rich repeat protein, partial |
| SLOPH_2689 | Leucine rich repeat protein, partial |
| SLOPH_2610 | Leucine rich repeat protein, partial |
| SLOPH_2148 | Leucine rich repeat protein, partial |
| SLOPH_1309 | Leucine rich repeat protein, partial |
| SLOPH_1310 | Leucine rich repeat protein, partial |
| SLOPH_1311 | Leucine rich repeat protein, partial |
| SLOPH_1247 | Leucine rich repeat protein, partial |
| SLOPH_2676 | Leucine rich repeat protein, partial |
| SLOPH_2639 | Leucine rich repeat protein, partial |
| SLOPH_2583 | Leucine rich repeat protein, partial |
| SLOPH_2690 | Leucine rich repeat protein, partial |
| SLOPH_2691 | Leucine rich repeat protein, partial |
| SLOPH_2584 | Leucine rich repeat protein, partial |
| SLOPH_1256 | Leucine rich repeat protein, partial |
| SLOPH_1257 | Leucine rich repeat protein, partial |
| SLOPH_2465 | Leucine rich repeat protein, partial |
| SLOPH_2652 | Leucine rich repeat protein, partial |
| SLOPH_2640 | Leucine rich repeat protein, partial |
| SLOPH_1252 | Leucine rich repeat protein, partial |
| SLOPH_2688 | Leucine rich repeat protein, partial |
| SLOPH_1324 | Leucine rich repeat protein, partial |
| SLOPH_2654 | Leucine rich repeat protein, partial |
| SLOPH_2607 | Leucine rich repeat protein, partial |
| SLOPH_2608 | Leucine rich repeat protein, partial |
| SLOPH_2641 | Leucine rich repeat protein, partial |
| SLOPH_1267 | Leucine rich repeat protein, partial |
| SLOPH_2525 | Leucine rich repeat protein, partial |
| SLOPH_2642 | Leucine rich repeat protein, partial |
| SLOPH_2643 | Leucine rich repeat protein, partial |
| SLOPH_1276 | Leucine rich repeat protein, partial |
| SLOPH_1277 | Leucine rich repeat protein, partial |
| SLOPH_2650 | Leucine rich repeat protein, partial |
| SLOPH_2651 | leucine rich repeat protein, partial |
| SLOPH_2644 | Leucine rich repeat protein, partial |
| SLOPH_2645 | Leucine rich repeat protein, partial |
| SLOPH_2631 | Leucine rich repeat protein, partial |
| SLOPH_2413 | Peptidase C19 |
| SLOPH_2321 | Peptidase C19 contains ubiquitinyl hydrolase |
| SLOPH_2414 | Peptidase C19, partial |
| SLOPH_1423 | pfkB family carbohydrate kinase |
| SLOPH_1498 | Protein kinase |
| SLOPH_1499 | Protein kinase |
| SLOPH_1500 | Protein kinase |
| SLOPH_1501 | Protein kinase |
| SLOPH_1503 | Protein kinase |
| SLOPH_1505 | Protein kinase |
| SLOPH_2108 | Protein kinase |
| SLOPH_1495 | Protein kinase |
| SLOPH_1511 | Protein kinase domain containing protein |
| SLOPH_2603 | Protein kinase, partial |
| SLOPH_1199 | Putative transposase |
| SLOPH_2111 | Putative transposon |
| SLOPH_1574 | Ribosomal protein S6 kinase |
| SLOPH_1637 | Serine/threonine kinase |
| SLOPH_1629 | Serine/threonine protein kinase |
| SLOPH_1622 | Serine/threonine protein kinase |
| SLOPH_2399 | serine/threonine-protein kinase |
| SLOPH_1634 | Serine/threonine-protein kinase |
| SLOPH_1914 | Sulfate transporter |
| SLOPH_1917 | Sulfate transporter protein |
| SLOPH_2024 | Ubiquitin carboxyl-terminal hydrolase |
| SLOPH_2023 | Ubiquitin carboxyl-terminal hydrolase |
| SLOPH_2627 | UBP5, Ubiquitin C-terminal hydrolase |

Singleton *Spraguea lophii* proteins that are not found in *Homo sapiens* and *Saccharomyces cerevisiae*, *Enterocytozoon bieneusi*, *Encephalitozoon cuniculi*, *Nosema ceranae* or *Nematocida parisii*):

| SLOPH_2472 | 26S proteasome regulatory subunit 4, partial |
| --- | --- |
| SLOPH_52 | 60s acidic ribosomal protein |
| SLOPH_84 | AAA domain dynein-related subfamily protein, partial |
| SLOPH_2370 | ABC transporter |
| SLOPH_2708 | ABC transporter protein, partial |
| SLOPH_96 | ABC1 family protein, partial |
| SLOPH_2362 | Acetyltransferase GNAT family protein |
| SLOPH_118 | ADP/ATP carrier protein |
| SLOPH_120 | Aldose reductase |
| SLOPH_2214 | APS2 Clathrin adaptor complex small subunit |
| SLOPH_137 | ARID/BRIGHT DNA binding domain protein |
| SLOPH_140 | Arv1-like protein |
| SLOPH_159 | Biotin acetyl CoA carboxylase ligase, partial |
| SLOPH_2502 | Biotin-requiring enzyme, partial |
| SLOPH_175 | Calmodulin |
| SLOPH_2265 | Cell division control protein Cdc48, AAA+ ATPasefamily |
| SLOPH_2519 | Cell division kinase, partial |
| SLOPH_217 | Coatomer protein complex subunit alpha |
| SLOPH_221 | Condensin-like protein, partial |
| SLOPH_2236 | Crooked neck-like protein 1 |
| SLOPH_254 | Cytidylate kinase family protein |
| SLOPH_2714 | cytochrome b5 type B-like protein |
| SLOPH_261 | DEAD box RNA helicase |
| SLOPH_265 | DEAD/DEAH box helicase |
| SLOPH_285 | DNA mismatch repair protein Msh5 |
| SLOPH_288 | DNA mismatch repair protein MutS |
| SLOPH_2256 | DnaJ-like protein |
| SLOPH_2699 | Gamma tubulin, partial |
| SLOPH_392 | Glucose-6-phosphate isomerase |
| SLOPH_2622 | Glutamate-ammonia ligase |
| SLOPH_400 | Glycerol-3-phosphate dehydrogenase NAD(P) |
| SLOPH_405 | GNAT acetyltransferase, partial |
| SLOPH_2379 | Haloacid dehalogenase |
| SLOPH_443 | Homeobox protein |
| SLOPH_452 | Hydroxyacylglutathione hydrolase |
| SLOPH_1000 | hypothetical protein SLOPH 1000, partial |
| SLOPH_1001 | hypothetical protein SLOPH 1001 |
| SLOPH_1004 | hypothetical protein SLOPH 1004 |
| SLOPH_1005 | hypothetical protein SLOPH 1005, partial |
| SLOPH_1007 | hypothetical protein SLOPH 1007 |
| SLOPH_1008 | hypothetical protein SLOPH 1008 |
| SLOPH_1009 | hypothetical protein SLOPH 1009 |
| SLOPH_1010 | hypothetical protein SLOPH 1010, partial |
| SLOPH_1011 | hypothetical protein SLOPH 1011 |
| SLOPH_1015 | hypothetical protein SLOPH 1015 |
| SLOPH_1016 | hypothetical protein SLOPH 1016 |
| SLOPH_1017 | hypothetical protein SLOPH 1017 |
| SLOPH_1018 | hypothetical protein SLOPH 1018 |
| SLOPH_1021 | hypothetical protein SLOPH 1021, partial |
| SLOPH_1022 | hypothetical protein SLOPH 1022, partial |
| SLOPH_1025 | hypothetical protein SLOPH 1025 |
| SLOPH_1026 | hypothetical protein SLOPH 1026 |
| SLOPH_1027 | hypothetical protein SLOPH 1027 |
| SLOPH_1028 | hypothetical protein SLOPH 1028 |
| SLOPH_1029 | hypothetical protein SLOPH 1029 |
| SLOPH_1030 | hypothetical protein SLOPH 1030 |
| SLOPH_1031 | hypothetical protein SLOPH 1031 |
| SLOPH_1033 | hypothetical protein SLOPH 1033, partial |
| SLOPH_1034 | hypothetical protein SLOPH 1034 |
| SLOPH_1035 | hypothetical protein SLOPH 1035 |
| SLOPH_1036 | hypothetical protein SLOPH 1036 |
| SLOPH_1037 | hypothetical protein SLOPH 1037 |
| SLOPH_1039 | hypothetical protein SLOPH 1039, partial |
| SLOPH_1040 | hypothetical protein SLOPH 1040, partial |
| SLOPH_1042 | hypothetical protein SLOPH 1042 |
| SLOPH_1043 | hypothetical protein SLOPH 1043 |
| SLOPH_1045 | hypothetical protein SLOPH 1045 |
| SLOPH_1046 | hypothetical protein SLOPH 1046 |
| SLOPH_1049 | hypothetical protein SLOPH 1049, partial |
| SLOPH_1050 | hypothetical protein SLOPH 1050 |
| SLOPH_1053 | hypothetical protein SLOPH 1053 |
| SLOPH_1055 | hypothetical protein SLOPH 1055 |
| SLOPH_1057 | hypothetical protein SLOPH 1057 |
| SLOPH_1058 | hypothetical protein SLOPH 1058 |
| SLOPH_1059 | hypothetical protein SLOPH 1059 |
| SLOPH_1060 | hypothetical protein SLOPH 1060 |
| SLOPH_1063 | hypothetical protein SLOPH 1063, partial |
| SLOPH_1065 | hypothetical protein SLOPH 1065 |
| SLOPH_1066 | hypothetical protein SLOPH 1066 |
| SLOPH_1067 | hypothetical protein SLOPH 1067 |
| SLOPH_1070 | hypothetical protein SLOPH 1070 |
| SLOPH_1071 | hypothetical protein SLOPH 1071 |
| SLOPH_1072 | hypothetical protein SLOPH 1072 |
| SLOPH_1074 | hypothetical protein SLOPH 1074, partial |
| SLOPH_1075 | hypothetical protein SLOPH 1075, partial |
| SLOPH_1076 | hypothetical protein SLOPH 1076 |
| SLOPH_1077 | hypothetical protein SLOPH 1077 |
| SLOPH_1078 | hypothetical protein SLOPH 1078 |
| SLOPH_1081 | hypothetical protein SLOPH 1081 |
| SLOPH_1083 | hypothetical protein SLOPH 1083 |
| SLOPH_1084 | hypothetical protein SLOPH 1084, partial |
| SLOPH_1086 | hypothetical protein SLOPH 1086, partial |
| SLOPH_1088 | hypothetical protein SLOPH 1088 |
| SLOPH_1089 | hypothetical protein SLOPH 1089, partial |
| SLOPH_1090 | hypothetical protein SLOPH 1090, partial |
| SLOPH_1092 | hypothetical protein SLOPH 1092 |
| SLOPH_1093 | hypothetical protein SLOPH 1093, partial |
| SLOPH_1095 | hypothetical protein SLOPH 1095 |
| SLOPH_1097 | hypothetical protein SLOPH 1097, partial |
| SLOPH_1099 | hypothetical protein SLOPH 1099 |
| SLOPH_1100 | hypothetical protein SLOPH 1100 |
| SLOPH_1101 | hypothetical protein SLOPH 1101 |
| SLOPH_1102 | hypothetical protein SLOPH 1102, partial |
| SLOPH_1103 | hypothetical protein SLOPH 1103 |
| SLOPH_1105 | hypothetical protein SLOPH 1105, partial |
| SLOPH_1107 | hypothetical protein SLOPH 1107, partial |
| SLOPH_1109 | hypothetical protein SLOPH 1109 |
| SLOPH_1111 | hypothetical protein SLOPH 1111, partial |
| SLOPH_1113 | hypothetical protein SLOPH 1113, partial |
| SLOPH_1115 | hypothetical protein SLOPH 1115 |
| SLOPH_1117 | hypothetical protein SLOPH 1117, partial |
| SLOPH_1118 | hypothetical protein SLOPH 1118 |
| SLOPH_1119 | hypothetical protein SLOPH 1119, partial |
| SLOPH_1123 | hypothetical protein SLOPH 1123 |
| SLOPH_1124 | hypothetical protein SLOPH 1124, partial |
| SLOPH_1125 | hypothetical protein SLOPH 1125 |
| SLOPH_1127 | hypothetical protein SLOPH 1127 |
| SLOPH_1128 | hypothetical protein SLOPH 1128 |
| SLOPH_1129 | hypothetical protein SLOPH 1129, partial |
| SLOPH_1130 | hypothetical protein SLOPH 1130 |
| SLOPH_1131 | hypothetical protein SLOPH 1131 |
| SLOPH_1132 | hypothetical protein SLOPH 1132 |
| SLOPH_1133 | hypothetical protein SLOPH 1133 |
| SLOPH_1134 | hypothetical protein SLOPH 1134 |
| SLOPH_1136 | hypothetical protein SLOPH 1136 |
| SLOPH_1137 | hypothetical protein SLOPH 1137, partial |
| SLOPH_1138 | hypothetical protein SLOPH 1138, partial |
| SLOPH_1140 | hypothetical protein SLOPH 1140, partial |
| SLOPH_1143 | hypothetical protein SLOPH 1143 |
| SLOPH_1144 | hypothetical protein SLOPH 1144 |
| SLOPH_1145 | hypothetical protein SLOPH 1145 |
| SLOPH_1146 | hypothetical protein SLOPH 1146 |
| SLOPH_1147 | hypothetical protein SLOPH 1147 |
| SLOPH_1148 | hypothetical protein SLOPH 1148 |
| SLOPH_1149 | hypothetical protein SLOPH 1149, partial |
| SLOPH_1150 | hypothetical protein SLOPH 1150 |
| SLOPH_1154 | hypothetical protein SLOPH 1154 |
| SLOPH_1155 | hypothetical protein SLOPH 1155, partial |
| SLOPH_1156 | hypothetical protein SLOPH 1156 |
| SLOPH_1157 | hypothetical protein SLOPH 1157 |
| SLOPH_1158 | hypothetical protein SLOPH 1158 |
| SLOPH_1159 | hypothetical protein SLOPH 1159, partial |
| SLOPH_1162 | hypothetical protein SLOPH 1162 |
| SLOPH_1163 | hypothetical protein SLOPH 1163 |
| SLOPH_1164 | hypothetical protein SLOPH 1164 |
| SLOPH_1166 | hypothetical protein SLOPH 1166, partial |
| SLOPH_1168 | hypothetical protein SLOPH 1168 |
| SLOPH_1169 | hypothetical protein SLOPH 1169 |
| SLOPH_1170 | hypothetical protein SLOPH 1170 |
| SLOPH_1171 | hypothetical protein SLOPH 1171 |
| SLOPH_1172 | hypothetical protein SLOPH 1172 |
| SLOPH_1173 | hypothetical protein SLOPH 1173 |
| SLOPH_1174 | hypothetical protein SLOPH 1174, partial |
| SLOPH_1175 | hypothetical protein SLOPH 1175 |
| SLOPH_1176 | hypothetical protein SLOPH 1176, partial |
| SLOPH_1177 | hypothetical protein SLOPH 1177 |
| SLOPH_1179 | hypothetical protein SLOPH 1179, partial |
| SLOPH_1180 | hypothetical protein SLOPH 1180 |
| SLOPH_1181 | hypothetical protein SLOPH 1181 |
| SLOPH_1182 | hypothetical protein SLOPH 1182 |
| SLOPH_1183 | hypothetical protein SLOPH 1183, partial |
| SLOPH_1184 | hypothetical protein SLOPH 1184, partial |
| SLOPH_1185 | hypothetical protein SLOPH 1185, partial |
| SLOPH_1187 | hypothetical protein SLOPH 1187, partial |
| SLOPH_1188 | hypothetical protein SLOPH 1188, partial |
| SLOPH_1190 | hypothetical protein SLOPH 1190 |
| SLOPH_1193 | hypothetical protein SLOPH 1193 |
| SLOPH_1194 | hypothetical protein SLOPH 1194 |
| SLOPH_1195 | hypothetical protein SLOPH 1195, partial |
| SLOPH_1196 | hypothetical protein SLOPH 1196, partial |
| SLOPH_1197 | hypothetical protein SLOPH 1197 |
| SLOPH_1198 | hypothetical protein SLOPH 1198 |
| SLOPH_1215 | hypothetical protein SLOPH 1215, partial |
| SLOPH_1219 | hypothetical protein SLOPH 1219 |
| SLOPH_1221 | hypothetical protein SLOPH 1221, partial |
| SLOPH_1222 | hypothetical protein SLOPH 1222, partial |
| SLOPH_1223 | hypothetical protein SLOPH 1223 |
| SLOPH_1224 | hypothetical protein SLOPH 1224, partial |
| SLOPH_1225 | hypothetical protein SLOPH 1225 |
| SLOPH_1227 | hypothetical protein SLOPH 1227 |
| SLOPH_1654 | hypothetical protein SLOPH 1654 |
| SLOPH_1670 | hypothetical protein SLOPH 1670 |
| SLOPH_1687 | hypothetical protein SLOPH 1687 |
| SLOPH_1692 | hypothetical protein SLOPH 1692, partial |
| SLOPH_1698 | hypothetical protein SLOPH 1698 |
| SLOPH_1702 | hypothetical protein SLOPH 1702 |
| SLOPH_1723 | hypothetical protein SLOPH 1723 |
| SLOPH_1725 | hypothetical protein SLOPH 1725, partial |
| SLOPH_1729 | hypothetical protein SLOPH 1729 |
| SLOPH_1730 | hypothetical protein SLOPH 1730, partial |
| SLOPH_1741 | hypothetical protein SLOPH 1741 |
| SLOPH_1742 | hypothetical protein SLOPH 1742 |
| SLOPH_1745 | hypothetical protein SLOPH 1745 |
| SLOPH_1746 | hypothetical protein SLOPH 1746 |
| SLOPH_1755 | hypothetical protein SLOPH 1755, partial |
| SLOPH_1781 | hypothetical protein SLOPH 1781 |
| SLOPH_1793 | hypothetical protein SLOPH 1793, partial |
| SLOPH_1798 | hypothetical protein SLOPH 1798 |
| SLOPH_1801 | hypothetical protein SLOPH 1801 |
| SLOPH_1822 | hypothetical protein SLOPH 1822, partial |
| SLOPH_1823 | hypothetical protein SLOPH 1823 |
| SLOPH_1830 | hypothetical protein SLOPH 1830, partial |
| SLOPH_1832 | hypothetical protein SLOPH 1832 |
| SLOPH_1842 | hypothetical protein SLOPH 1842, partial |
| SLOPH_1843 | hypothetical protein SLOPH 1843 |
| SLOPH_1858 | hypothetical protein SLOPH 1858, partial |
| SLOPH_1867 | hypothetical protein SLOPH 1867 |
| SLOPH_1869 | hypothetical protein SLOPH 1869, partial |
| SLOPH_1871 | hypothetical protein SLOPH 1871 |
| SLOPH_1879 | hypothetical protein SLOPH 1879 |
| SLOPH_2094 | hypothetical protein SLOPH 2094, partial |
| SLOPH_2110 | hypothetical protein SLOPH 2110 |
| SLOPH_2114 | hypothetical protein SLOPH 2114 |
| SLOPH_2120 | hypothetical protein SLOPH 2120 |
| SLOPH_2124 | hypothetical protein SLOPH 2124 |
| SLOPH_2156 | hypothetical protein SLOPH 2156 |
| SLOPH_2158 | hypothetical protein SLOPH 2158 |
| SLOPH_2159 | hypothetical protein SLOPH 2159 |
| SLOPH_2167 | hypothetical protein SLOPH 2167 |
| SLOPH_2175 | hypothetical protein SLOPH 2175, partial |
| SLOPH_2178 | hypothetical protein SLOPH 2178 |
| SLOPH_2180 | hypothetical protein SLOPH 2180 |
| SLOPH_2186 | hypothetical protein SLOPH 2186 |
| SLOPH_2187 | hypothetical protein SLOPH 2187 |
| SLOPH_2188 | hypothetical protein SLOPH 2188 |
| SLOPH_2198 | hypothetical protein SLOPH 2198 |
| SLOPH_2207 | hypothetical protein SLOPH 2207 |
| SLOPH_2208 | hypothetical protein SLOPH 2208 |
| SLOPH_2219 | hypothetical protein SLOPH 2219, partial |
| SLOPH_2222 | hypothetical protein SLOPH 2222 |
| SLOPH_2225 | hypothetical protein SLOPH 2225 |
| SLOPH_223 | hypothetical protein SLOPH 223 |
| SLOPH_2230 | hypothetical protein SLOPH 2230 |
| SLOPH_224 | hypothetical protein SLOPH 224 |
| SLOPH_2241 | hypothetical protein SLOPH 2241 |
| SLOPH_2261 | hypothetical protein SLOPH 2261 |
| SLOPH_2263 | hypothetical protein SLOPH 2263 |
| SLOPH_2274 | hypothetical protein SLOPH 2274, partial |
| SLOPH_2276 | hypothetical protein SLOPH 2276 |
| SLOPH_2281 | hypothetical protein SLOPH 2281 |
| SLOPH_2283 | hypothetical protein SLOPH 2283 |
| SLOPH_2291 | hypothetical protein SLOPH 2291 |
| SLOPH_2297 | hypothetical protein SLOPH 2297 |
| SLOPH_230 | hypothetical protein SLOPH 230 |
| SLOPH_2302 | hypothetical protein SLOPH 2302 |
| SLOPH_2304 | hypothetical protein SLOPH 2304, partial |
| SLOPH_2311 | hypothetical protein SLOPH 2311 |
| SLOPH_2314 | hypothetical protein SLOPH 2314, partial |
| SLOPH_2318 | hypothetical protein SLOPH 2318 |
| SLOPH_2320 | hypothetical protein SLOPH 2320 |
| SLOPH_2322 | hypothetical protein SLOPH 2322 |
| SLOPH_2326 | hypothetical protein SLOPH 2326 |
| SLOPH_233 | hypothetical protein SLOPH 233 |
| SLOPH_2333 | hypothetical protein SLOPH 2333 |
| SLOPH_2339 | hypothetical protein SLOPH 2339 |
| SLOPH_2345 | hypothetical protein SLOPH 2345 |
| SLOPH_2355 | hypothetical protein SLOPH 2355 |
| SLOPH_2366 | hypothetical protein SLOPH 2366 |
| SLOPH_2374 | hypothetical protein SLOPH 2374 |
| SLOPH_2375 | hypothetical protein SLOPH 2375 |
| SLOPH_2376 | hypothetical protein SLOPH 2376 |
| SLOPH_2381 | hypothetical protein SLOPH 2381 |
| SLOPH_2389 | hypothetical protein SLOPH 2389 |
| SLOPH_2402 | hypothetical protein SLOPH 2402 |
| SLOPH_2404 | hypothetical protein SLOPH 2404, partial |
| SLOPH_2409 | hypothetical protein SLOPH 2409 |
| SLOPH_2410 | hypothetical protein SLOPH 2410 |
| SLOPH_2411 | hypothetical protein SLOPH 2411 |
| SLOPH_2426 | hypothetical protein SLOPH 2426 |
| SLOPH_2448 | hypothetical protein SLOPH 2448 |
| SLOPH_2459 | hypothetical protein SLOPH 2459 |
| SLOPH_2460 | hypothetical protein SLOPH 2460 |
| SLOPH_2471 | hypothetical protein SLOPH 2471 |
| SLOPH_2483 | hypothetical protein SLOPH 2483, partial |
| SLOPH_2484 | hypothetical protein SLOPH 2484 |
| SLOPH_2489 | hypothetical protein SLOPH 2489 |
| SLOPH_2490 | hypothetical protein SLOPH 2490 |
| SLOPH_2491 | hypothetical protein SLOPH 2491 |
| SLOPH_2492 | hypothetical protein SLOPH 2492 |
| SLOPH_2494 | hypothetical protein SLOPH 2494, partial |
| SLOPH_2495 | hypothetical protein SLOPH 2495 |
| SLOPH_2496 | hypothetical protein SLOPH 2496, partial |
| SLOPH_2498 | hypothetical protein SLOPH 2498, partial |
| SLOPH_2500 | hypothetical protein SLOPH 2500 |
| SLOPH_2501 | hypothetical protein SLOPH 2501 |
| SLOPH_2511 | hypothetical protein SLOPH 2511, partial |
| SLOPH_2514 | hypothetical protein SLOPH 2514, partial |
| SLOPH_2521 | hypothetical protein SLOPH 2521 |
| SLOPH_2522 | hypothetical protein SLOPH 2522 |
| SLOPH_2523 | hypothetical protein SLOPH 2523, partial |
| SLOPH_2528 | hypothetical protein SLOPH 2528, partial |
| SLOPH_2529 | hypothetical protein SLOPH 2529 |
| SLOPH_2531 | hypothetical protein SLOPH 2531 |
| SLOPH_2535 | hypothetical protein SLOPH 2535 |
| SLOPH_2539 | hypothetical protein SLOPH 2539, partial |
| SLOPH_2540 | hypothetical protein SLOPH 2540, partial |
| SLOPH_2541 | hypothetical protein SLOPH 2541 |
| SLOPH_2543 | hypothetical protein SLOPH 2543 |
| SLOPH_2550 | hypothetical protein SLOPH 2550, partial |
| SLOPH_2551 | hypothetical protein SLOPH 2551, partial |
| SLOPH_2560 | hypothetical protein SLOPH 2560, partial |
| SLOPH_2561 | hypothetical protein SLOPH 2561 |
| SLOPH_2562 | hypothetical protein SLOPH 2562, partial |
| SLOPH_2564 | hypothetical protein SLOPH 2564 |
| SLOPH_2566 | hypothetical protein SLOPH 2566 |
| SLOPH_2567 | hypothetical protein SLOPH 2567, partial |
| SLOPH_2568 | hypothetical protein SLOPH 2568, partial |
| SLOPH_2569 | hypothetical protein SLOPH 2569, partial |
| SLOPH_2570 | hypothetical protein SLOPH 2570, partial |
| SLOPH_2572 | hypothetical protein SLOPH 2572, partial |
| SLOPH_2573 | hypothetical protein SLOPH 2573 |
| SLOPH_2574 | hypothetical protein SLOPH 2574, partial |
| SLOPH_2578 | hypothetical protein SLOPH 2578, partial |
| SLOPH_2580 | hypothetical protein SLOPH 2580, partial |
| SLOPH_2582 | hypothetical protein SLOPH 2582 |
| SLOPH_2586 | hypothetical protein SLOPH 2586, partial |
| SLOPH_2626 | hypothetical protein SLOPH 2626 |
| SLOPH_2687 | hypothetical protein SLOPH 2687, partial |
| SLOPH_2707 | hypothetical protein SLOPH 2707 |
| SLOPH_2712 | hypothetical protein SLOPH 2712, partial |
| SLOPH_2727 | hypothetical protein SLOPH 2727, partial |
| SLOPH_2728 | hypothetical protein SLOPH 2728 |
| SLOPH_2729 | hypothetical protein SLOPH 2729, partial |
| SLOPH_2737 | hypothetical protein SLOPH 2737 |
| SLOPH_2743 | hypothetical protein SLOPH 2743 |
| SLOPH_2745 | hypothetical protein SLOPH 2745 |
| SLOPH_453 | hypothetical protein SLOPH 453, partial |
| SLOPH_456 | hypothetical protein SLOPH 456 |
| SLOPH_458 | hypothetical protein SLOPH 458 |
| SLOPH_460 | hypothetical protein SLOPH 460 |
| SLOPH_461 | hypothetical protein SLOPH 461 |
| SLOPH_462 | hypothetical protein SLOPH 462 |
| SLOPH_463 | hypothetical protein SLOPH 463, partial |
| SLOPH_465 | hypothetical protein SLOPH 465 |
| SLOPH_468 | hypothetical protein SLOPH 468 |
| SLOPH_469 | hypothetical protein SLOPH 469, partial |
| SLOPH_470 | hypothetical protein SLOPH 470 |
| SLOPH_472 | hypothetical protein SLOPH 472 |
| SLOPH_473 | hypothetical protein SLOPH 473 |
| SLOPH_474 | hypothetical protein SLOPH 474, partial |
| SLOPH_476 | hypothetical protein SLOPH 476 |
| SLOPH_477 | hypothetical protein SLOPH 477 |
| SLOPH_479 | hypothetical protein SLOPH 479 |
| SLOPH_480 | hypothetical protein SLOPH 480 |
| SLOPH_481 | hypothetical protein SLOPH 481 |
| SLOPH_483 | hypothetical protein SLOPH 483, partial |
| SLOPH_485 | hypothetical protein SLOPH 485 |
| SLOPH_487 | hypothetical protein SLOPH 487, partial |
| SLOPH_489 | hypothetical protein SLOPH 489 |
| SLOPH_490 | hypothetical protein SLOPH 490, partial |
| SLOPH_491 | hypothetical protein SLOPH 491, partial |
| SLOPH_492 | hypothetical protein SLOPH 492 |
| SLOPH_493 | hypothetical protein SLOPH 493 |
| SLOPH_494 | hypothetical protein SLOPH 494 |
| SLOPH_497 | hypothetical protein SLOPH 497, partial |
| SLOPH_498 | hypothetical protein SLOPH 498 |
| SLOPH_499 | hypothetical protein SLOPH 499, partial |
| SLOPH_500 | hypothetical protein SLOPH 500, partial |
| SLOPH_501 | hypothetical protein SLOPH 501, partial |
| SLOPH_502 | hypothetical protein SLOPH 502 |
| SLOPH_503 | hypothetical protein SLOPH 503 |
| SLOPH_506 | hypothetical protein SLOPH 506, partial |
| SLOPH_508 | hypothetical protein SLOPH 508 |
| SLOPH_510 | hypothetical protein SLOPH 510, partial |
| SLOPH_511 | hypothetical protein SLOPH 511 |
| SLOPH_513 | hypothetical protein SLOPH 513, partial |
| SLOPH_514 | hypothetical protein SLOPH 514, partial |
| SLOPH_516 | hypothetical protein SLOPH 516, partial |
| SLOPH_518 | hypothetical protein SLOPH 518, partial |
| SLOPH_520 | hypothetical protein SLOPH 520, partial |
| SLOPH_521 | hypothetical protein SLOPH 521, partial |
| SLOPH_522 | hypothetical protein SLOPH 522 |
| SLOPH_523 | hypothetical protein SLOPH 523, partial |
| SLOPH_524 | hypothetical protein SLOPH 524, partial |
| SLOPH_526 | hypothetical protein SLOPH 526, partial |
| SLOPH_530 | hypothetical protein SLOPH 530 |
| SLOPH_531 | hypothetical protein SLOPH 531 |
| SLOPH_532 | hypothetical protein SLOPH 532, partial |
| SLOPH_533 | hypothetical protein SLOPH 533, partial |
| SLOPH_534 | hypothetical protein SLOPH 534 |
| SLOPH_535 | hypothetical protein SLOPH 535 |
| SLOPH_537 | hypothetical protein SLOPH 537 |
| SLOPH_538 | hypothetical protein SLOPH 538 |
| SLOPH_539 | hypothetical protein SLOPH 539 |
| SLOPH_541 | hypothetical protein SLOPH 541, partial |
| SLOPH_542 | hypothetical protein SLOPH 542, partial |
| SLOPH_543 | hypothetical protein SLOPH 543 |
| SLOPH_545 | hypothetical protein SLOPH 545 |
| SLOPH_546 | hypothetical protein SLOPH 546, partial |
| SLOPH_547 | hypothetical protein SLOPH 547 |
| SLOPH_548 | hypothetical protein SLOPH 548, partial |
| SLOPH_549 | hypothetical protein SLOPH 549, partial |
| SLOPH_550 | hypothetical protein SLOPH 550, partial |
| SLOPH_551 | hypothetical protein SLOPH 551 |
| SLOPH_553 | hypothetical protein SLOPH 553 |
| SLOPH_554 | hypothetical protein SLOPH 554 |
| SLOPH_555 | hypothetical protein SLOPH 555 |
| SLOPH_557 | hypothetical protein SLOPH 557 |
| SLOPH_560 | hypothetical protein SLOPH 560 |
| SLOPH_564 | hypothetical protein SLOPH 564, partial |
| SLOPH_565 | hypothetical protein SLOPH 565 |
| SLOPH_566 | hypothetical protein SLOPH 566, partial |
| SLOPH_567 | hypothetical protein SLOPH 567, partial |
| SLOPH_568 | hypothetical protein SLOPH 568 |
| SLOPH_569 | hypothetical protein SLOPH 569, partial |
| SLOPH_572 | hypothetical protein SLOPH 572, partial |
| SLOPH_577 | hypothetical protein SLOPH 577 |
| SLOPH_578 | hypothetical protein SLOPH 578 |
| SLOPH_579 | hypothetical protein SLOPH 579 |
| SLOPH_581 | hypothetical protein SLOPH 581 |
| SLOPH_582 | hypothetical protein SLOPH 582 |
| SLOPH_583 | hypothetical protein SLOPH 583 |
| SLOPH_585 | hypothetical protein SLOPH 585 |
| SLOPH_587 | hypothetical protein SLOPH 587, partial |
| SLOPH_590 | hypothetical protein SLOPH 590, partial |
| SLOPH_592 | hypothetical protein SLOPH 592 |
| SLOPH_593 | hypothetical protein SLOPH 593, partial |
| SLOPH_594 | hypothetical protein SLOPH 594 |
| SLOPH_595 | hypothetical protein SLOPH 595 |
| SLOPH_597 | hypothetical protein SLOPH 597 |
| SLOPH_598 | hypothetical protein SLOPH 598, partial |
| SLOPH_599 | hypothetical protein SLOPH 599, partial |
| SLOPH_602 | hypothetical protein SLOPH 602, partial |
| SLOPH_604 | hypothetical protein SLOPH 604, partial |
| SLOPH_605 | hypothetical protein SLOPH 605, partial |
| SLOPH_606 | hypothetical protein SLOPH 606, partial |
| SLOPH_609 | hypothetical protein SLOPH 609, partial |
| SLOPH_610 | hypothetical protein SLOPH 610, partial |
| SLOPH_613 | hypothetical protein SLOPH 613, partial |
| SLOPH_615 | hypothetical protein SLOPH 615, partial |
| SLOPH_616 | hypothetical protein SLOPH 616 |
| SLOPH_617 | hypothetical protein SLOPH 617 |
| SLOPH_618 | hypothetical protein SLOPH 618, partial |
| SLOPH_619 | hypothetical protein SLOPH 619, partial |
| SLOPH_620 | hypothetical protein SLOPH 620 |
| SLOPH_621 | hypothetical protein SLOPH 621 |
| SLOPH_622 | hypothetical protein SLOPH 622 |
| SLOPH_623 | hypothetical protein SLOPH 623 |
| SLOPH_624 | hypothetical protein SLOPH 624 |
| SLOPH_627 | hypothetical protein SLOPH 627, partial |
| SLOPH_628 | hypothetical protein SLOPH 628 |
| SLOPH_630 | hypothetical protein SLOPH 630, partial |
| SLOPH_633 | hypothetical protein SLOPH 633 |
| SLOPH_637 | hypothetical protein SLOPH 637, partial |
| SLOPH_639 | hypothetical protein SLOPH 639 |
| SLOPH_640 | hypothetical protein SLOPH 640, partial |
| SLOPH_641 | hypothetical protein SLOPH 641 |
| SLOPH_642 | hypothetical protein SLOPH 642 |
| SLOPH_644 | hypothetical protein SLOPH 644, partial |
| SLOPH_645 | hypothetical protein SLOPH 645, partial |
| SLOPH_647 | hypothetical protein SLOPH 647 |
| SLOPH_648 | hypothetical protein SLOPH 648 |
| SLOPH_650 | hypothetical protein SLOPH 650, partial |
| SLOPH_651 | hypothetical protein SLOPH 651, partial |
| SLOPH_653 | hypothetical protein SLOPH 653, partial |
| SLOPH_654 | hypothetical protein SLOPH 654 |
| SLOPH_659 | hypothetical protein SLOPH 659 |
| SLOPH_660 | hypothetical protein SLOPH 660 |
| SLOPH_661 | hypothetical protein SLOPH 661, partial |
| SLOPH_662 | hypothetical protein SLOPH 662, partial |
| SLOPH_663 | hypothetical protein SLOPH 663 |
| SLOPH_665 | hypothetical protein SLOPH 665 |
| SLOPH_666 | hypothetical protein SLOPH 666, partial |
| SLOPH_667 | hypothetical protein SLOPH 667 |
| SLOPH_668 | hypothetical protein SLOPH 668 |
| SLOPH_669 | hypothetical protein SLOPH 669 |
| SLOPH_673 | hypothetical protein SLOPH 673 |
| SLOPH_675 | hypothetical protein SLOPH 675 |
| SLOPH_676 | hypothetical protein SLOPH 676 |
| SLOPH_677 | hypothetical protein SLOPH 677 |
| SLOPH_678 | hypothetical protein SLOPH 678, partial |
| SLOPH_683 | hypothetical protein SLOPH 683 |
| SLOPH_684 | hypothetical protein SLOPH 684 |
| SLOPH_686 | hypothetical protein SLOPH 686 |
| SLOPH_687 | hypothetical protein SLOPH 687 |
| SLOPH_689 | hypothetical protein SLOPH 689 |
| SLOPH_690 | hypothetical protein SLOPH 690 |
| SLOPH_692 | hypothetical protein SLOPH 692 |
| SLOPH_693 | hypothetical protein SLOPH 693 |
| SLOPH_695 | hypothetical protein SLOPH 695 |
| SLOPH_699 | hypothetical protein SLOPH 699, partial |
| SLOPH_702 | hypothetical protein SLOPH 702 |
| SLOPH_703 | hypothetical protein SLOPH 703 |
| SLOPH_704 | hypothetical protein SLOPH 704 |
| SLOPH_707 | hypothetical protein SLOPH 707 |
| SLOPH_708 | hypothetical protein SLOPH 708 |
| SLOPH_711 | hypothetical protein SLOPH 711 |
| SLOPH_712 | hypothetical protein SLOPH 712, partial |
| SLOPH_713 | hypothetical protein SLOPH 713 |
| SLOPH_714 | hypothetical protein SLOPH 714 |
| SLOPH_715 | hypothetical protein SLOPH 715, partial |
| SLOPH_717 | hypothetical protein SLOPH 717 |
| SLOPH_719 | hypothetical protein SLOPH 719, partial |
| SLOPH_720 | hypothetical protein SLOPH 720 |
| SLOPH_721 | hypothetical protein SLOPH 721 |
| SLOPH_723 | hypothetical protein SLOPH 723 |
| SLOPH_724 | hypothetical protein SLOPH 724 |
| SLOPH_728 | hypothetical protein SLOPH 728 |
| SLOPH_729 | hypothetical protein SLOPH 729, partial |
| SLOPH_730 | hypothetical protein SLOPH 730 |
| SLOPH_732 | hypothetical protein SLOPH 732 |
| SLOPH_734 | hypothetical protein SLOPH 734 |
| SLOPH_735 | hypothetical protein SLOPH 735, partial |
| SLOPH_738 | hypothetical protein SLOPH 738 |
| SLOPH_739 | hypothetical protein SLOPH 739 |
| SLOPH_740 | hypothetical protein SLOPH 740, partial |
| SLOPH_741 | hypothetical protein SLOPH 741 |
| SLOPH_742 | hypothetical protein SLOPH 742 |
| SLOPH_743 | hypothetical protein SLOPH 743 |
| SLOPH_744 | hypothetical protein SLOPH 744, partial |
| SLOPH_747 | hypothetical protein SLOPH 747 |
| SLOPH_749 | hypothetical protein SLOPH 749 |
| SLOPH_750 | hypothetical protein SLOPH 750 |
| SLOPH_751 | hypothetical protein SLOPH 751 |
| SLOPH_752 | hypothetical protein SLOPH 752 |
| SLOPH_753 | hypothetical protein SLOPH 753 |
| SLOPH_755 | hypothetical protein SLOPH 755, partial |
| SLOPH_758 | hypothetical protein SLOPH 758, partial |
| SLOPH_760 | hypothetical protein SLOPH 760, partial |
| SLOPH_761 | hypothetical protein SLOPH 761 |
| SLOPH_762 | hypothetical protein SLOPH 762 |
| SLOPH_763 | hypothetical protein SLOPH 763, partial |
| SLOPH_764 | hypothetical protein SLOPH 764 |
| SLOPH_765 | hypothetical protein SLOPH 765 |
| SLOPH_767 | hypothetical protein SLOPH 767 |
| SLOPH_769 | hypothetical protein SLOPH 769, partial |
| SLOPH_770 | hypothetical protein SLOPH 770, partial |
| SLOPH_771 | hypothetical protein SLOPH 771 |
| SLOPH_772 | hypothetical protein SLOPH 772 |
| SLOPH_773 | hypothetical protein SLOPH 773 |
| SLOPH_774 | hypothetical protein SLOPH 774 |
| SLOPH_775 | hypothetical protein SLOPH 775 |
| SLOPH_776 | hypothetical protein SLOPH 776 |
| SLOPH_777 | hypothetical protein SLOPH 777 |
| SLOPH_779 | hypothetical protein SLOPH 779 |
| SLOPH_783 | hypothetical protein SLOPH 783 |
| SLOPH_784 | hypothetical protein SLOPH 784 |
| SLOPH_788 | hypothetical protein SLOPH 788 |
| SLOPH_789 | hypothetical protein SLOPH 789 |
| SLOPH_790 | hypothetical protein SLOPH 790, partial |
| SLOPH_793 | hypothetical protein SLOPH 793 |
| SLOPH_794 | hypothetical protein SLOPH 794 |
| SLOPH_795 | hypothetical protein SLOPH 795 |
| SLOPH_796 | hypothetical protein SLOPH 796 |
| SLOPH_801 | hypothetical protein SLOPH 801 |
| SLOPH_802 | hypothetical protein SLOPH 802, partial |
| SLOPH_803 | hypothetical protein SLOPH 803 |
| SLOPH_804 | hypothetical protein SLOPH 804, partial |
| SLOPH_805 | hypothetical protein SLOPH 805, partial |
| SLOPH_807 | hypothetical protein SLOPH 807 |
| SLOPH_811 | hypothetical protein SLOPH 811, partial |
| SLOPH_812 | hypothetical protein SLOPH 812 |
| SLOPH_814 | hypothetical protein SLOPH 814, partial |
| SLOPH_815 | hypothetical protein SLOPH 815, partial |
| SLOPH_816 | hypothetical protein SLOPH 816 |
| SLOPH_817 | hypothetical protein SLOPH 817, partial |
| SLOPH_819 | hypothetical protein SLOPH 819, partial |
| SLOPH_820 | hypothetical protein SLOPH 820 |
| SLOPH_821 | hypothetical protein SLOPH 821 |
| SLOPH_823 | hypothetical protein SLOPH 823 |
| SLOPH_824 | hypothetical protein SLOPH 824 |
| SLOPH_825 | hypothetical protein SLOPH 825 |
| SLOPH_826 | hypothetical protein SLOPH 826, partial |
| SLOPH_828 | hypothetical protein SLOPH 828, partial |
| SLOPH_829 | hypothetical protein SLOPH 829 |
| SLOPH_832 | hypothetical protein SLOPH 832 |
| SLOPH_833 | hypothetical protein SLOPH 833 |
| SLOPH_834 | hypothetical protein SLOPH 834 |
| SLOPH_836 | hypothetical protein SLOPH 836 |
| SLOPH_837 | hypothetical protein SLOPH 837 |
| SLOPH_838 | hypothetical protein SLOPH 838, partial |
| SLOPH_839 | hypothetical protein SLOPH 839 |
| SLOPH_840 | hypothetical protein SLOPH 840 |
| SLOPH_841 | hypothetical protein SLOPH 841 |
| SLOPH_843 | hypothetical protein SLOPH 843 |
| SLOPH_844 | hypothetical protein SLOPH 844 |
| SLOPH_845 | hypothetical protein SLOPH 845 |
| SLOPH_846 | hypothetical protein SLOPH 846 |
| SLOPH_847 | hypothetical protein SLOPH 847, partial |
| SLOPH_848 | hypothetical protein SLOPH 848 |
| SLOPH_851 | hypothetical protein SLOPH 851 |
| SLOPH_852 | hypothetical protein SLOPH 852 |
| SLOPH_853 | hypothetical protein SLOPH 853 |
| SLOPH_854 | hypothetical protein SLOPH 854, partial |
| SLOPH_855 | hypothetical protein SLOPH 855 |
| SLOPH_856 | hypothetical protein SLOPH 856, partial |
| SLOPH_857 | hypothetical protein SLOPH 857 |
| SLOPH_861 | hypothetical protein SLOPH 861 |
| SLOPH_862 | hypothetical protein SLOPH 862 |
| SLOPH_865 | hypothetical protein SLOPH 865, partial |
| SLOPH_867 | hypothetical protein SLOPH 867 |
| SLOPH_869 | hypothetical protein SLOPH 869 |
| SLOPH_870 | hypothetical protein SLOPH 870 |
| SLOPH_871 | hypothetical protein SLOPH 871 |
| SLOPH_872 | hypothetical protein SLOPH 872, partial |
| SLOPH_874 | hypothetical protein SLOPH 874, partial |
| SLOPH_876 | hypothetical protein SLOPH 876, partial |
| SLOPH_877 | hypothetical protein SLOPH 877 |
| SLOPH_878 | hypothetical protein SLOPH 878 |
| SLOPH_879 | hypothetical protein SLOPH 879 |
| SLOPH_881 | hypothetical protein SLOPH 881, partial |
| SLOPH_882 | hypothetical protein SLOPH 882, partial |
| SLOPH_883 | hypothetical protein SLOPH 883, partial |
| SLOPH_884 | hypothetical protein SLOPH 884 |
| SLOPH_886 | hypothetical protein SLOPH 886 |
| SLOPH_887 | hypothetical protein SLOPH 887 |
| SLOPH_888 | hypothetical protein SLOPH 888 |
| SLOPH_891 | hypothetical protein SLOPH 891 |
| SLOPH_893 | hypothetical protein SLOPH 893 |
| SLOPH_896 | hypothetical protein SLOPH 896, partial |
| SLOPH_901 | hypothetical protein SLOPH 901, partial |
| SLOPH_903 | hypothetical protein SLOPH 903 |
| SLOPH_904 | hypothetical protein SLOPH 904 |
| SLOPH_905 | hypothetical protein SLOPH 905 |
| SLOPH_906 | hypothetical protein SLOPH 906 |
| SLOPH_908 | hypothetical protein SLOPH 908, partial |
| SLOPH_909 | hypothetical protein SLOPH 909, partial |
| SLOPH_910 | hypothetical protein SLOPH 910 |
| SLOPH_911 | hypothetical protein SLOPH 911, partial |
| SLOPH_912 | hypothetical protein SLOPH 912 |
| SLOPH_913 | hypothetical protein SLOPH 913 |
| SLOPH_914 | hypothetical protein SLOPH 914 |
| SLOPH_916 | hypothetical protein SLOPH 916, partial |
| SLOPH_917 | hypothetical protein SLOPH 917, partial |
| SLOPH_918 | hypothetical protein SLOPH 918, partial |
| SLOPH_920 | hypothetical protein SLOPH 920, partial |
| SLOPH_921 | hypothetical protein SLOPH 921, partial |
| SLOPH_922 | hypothetical protein SLOPH 922 |
| SLOPH_923 | hypothetical protein SLOPH 923 |
| SLOPH_925 | hypothetical protein SLOPH 925 |
| SLOPH_927 | hypothetical protein SLOPH 927, partial |
| SLOPH_928 | hypothetical protein SLOPH 928 |
| SLOPH_929 | hypothetical protein SLOPH 929 |
| SLOPH_932 | hypothetical protein SLOPH 932 |
| SLOPH_933 | hypothetical protein SLOPH 933 |
| SLOPH_934 | hypothetical protein SLOPH 934 |
| SLOPH_936 | hypothetical protein SLOPH 936, partial |
| SLOPH_937 | hypothetical protein SLOPH 937, partial |
| SLOPH_938 | hypothetical protein SLOPH 938 |
| SLOPH_939 | hypothetical protein SLOPH 939 |
| SLOPH_940 | hypothetical protein SLOPH 940 |
| SLOPH_941 | hypothetical protein SLOPH 941, partial |
| SLOPH_944 | hypothetical protein SLOPH 944 |
| SLOPH_945 | hypothetical protein SLOPH 945 |
| SLOPH_947 | hypothetical protein SLOPH 947 |
| SLOPH_950 | hypothetical protein SLOPH 950, partial |
| SLOPH_955 | hypothetical protein SLOPH 955 |
| SLOPH_957 | hypothetical protein SLOPH 957 |
| SLOPH_958 | hypothetical protein SLOPH 958 |
| SLOPH_960 | hypothetical protein SLOPH 960 |
| SLOPH_967 | hypothetical protein SLOPH 967 |
| SLOPH_969 | hypothetical protein SLOPH 969, partial |
| SLOPH_970 | hypothetical protein SLOPH 970 |
| SLOPH_971 | hypothetical protein SLOPH 971 |
| SLOPH_972 | hypothetical protein SLOPH 972 |
| SLOPH_973 | hypothetical protein SLOPH 973 |
| SLOPH_975 | hypothetical protein SLOPH 975, partial |
| SLOPH_976 | hypothetical protein SLOPH 976 |
| SLOPH_977 | hypothetical protein SLOPH 977 |
| SLOPH_978 | hypothetical protein SLOPH 978, partial |
| SLOPH_980 | hypothetical protein SLOPH 980 |
| SLOPH_984 | hypothetical protein SLOPH 984 |
| SLOPH_985 | hypothetical protein SLOPH 985, partial |
| SLOPH_989 | hypothetical protein SLOPH 989, partial |
| SLOPH_990 | hypothetical protein SLOPH 990 |
| SLOPH_992 | hypothetical protein SLOPH 992 |
| SLOPH_993 | hypothetical protein SLOPH 993 |
| SLOPH_994 | hypothetical protein SLOPH 994 |
| SLOPH_996 | hypothetical protein SLOPH 996 |
| SLOPH_997 | hypothetical protein SLOPH 997 |
| SLOPH_998 | hypothetical protein SLOPH 998 |
| SLOPH_999 | hypothetical protein SLOPH 999, partial |
| SLOPH_1234 | Isoprenylcysteine carboxyl methyltransferase |
| SLOPH_1240 | Kinesin family protein |
| SLOPH_1322 | Leucine rich protein |
| SLOPH_1301 | Leucine rich repeat protein |
| SLOPH_1253 | Leucine rich repeat protein |
| SLOPH_1299 | Leucine rich repeat protein |
| SLOPH_1305 | Leucine rich repeat protein |
| SLOPH_2563 | Leucine rich repeat protein |
| SLOPH_1288 | Leucine rich repeat protein |
| SLOPH_1294 | Leucine rich repeat protein, partial |
| SLOPH_1308 | Leucine rich repeat protein, partial |
| SLOPH_1330 | Long-chain-fatty-acid-CoA ligase, partial |
| SLOPH_1332 | LSM domain protein |
| SLOPH_2542 | Lysine rich hypothetical protein, partial |
| SLOPH_2720 | MdlB type mitochondrial-type ABC transporter |
| SLOPH_1344 | Mechanosensitive ion channel |
| SLOPH_1361 | Mitochondrial-type translocse TOM70, partial |
| SLOPH_1371 | MUTS DNA mismatch repair protein-like protein, partial |
| SLOPH_1377 | Myosin heavy chain, partial |
| SLOPH_2659 | Nonsense-mediated mRNA decay protein, partial |
| SLOPH_1412 | Peptidase C50 family protein, partial |
| SLOPH_1415 | Peptidase M16 domain protein |
| SLOPH_1442 | Phospholipase A2 activating protein, partial |
| SLOPH_1449 | Phosphoprotein phosphatase, partial |
| SLOPH_1453 | PLAA family ubiquitin binding protein, partial |
| SLOPH_1455 | Plasma membrane calcium-transporting ATPase |
| SLOPH_2530 | Pol polyprotein |
| SLOPH_1481 | pre-mRNA splicing factor PRP8, partial |
| SLOPH_1480 | Pre-mRNA splicing factor, partial |
| SLOPH_1509 | Protein kinase |
| SLOPH_1506 | Protein kinase |
| SLOPH_1508 | Protein kinase |
| SLOPH_2655 | Protein kinase, partial |
| SLOPH_2724 | protein transport Sec61-like protein |
| SLOPH_1519 | Proton/peptide symporter family protein |
| SLOPH_1527 | Putative ATP binding protein, partial |
| SLOPH_1899 | putative DNA repair and recombination protein RAD26, partial |
| SLOPH_2705 | Putative transposase |
| SLOPH_1993 | Putative transposase |
| SLOPH_1546 | Replication factor A protein 3 |
| SLOPH_1557 | RhoGAP domain protein |
| SLOPH_1576 | Ribosome biogenesis regulatory protein, partial |
| SLOPH_1584 | RNA exonuclease 1 |
| SLOPH_1586 | RNA polymerase III subunit C17 |
| SLOPH_1603 | SANTA domain protein |
| SLOPH_1621 | Serine/threonine kinase |
| SLOPH_1624 | Serine/Threonine protein kinase |
| SLOPH_1636 | Serine/threonine protein kinase |
| SLOPH_1625 | Serine/threonine protein kinase, partial |
| SLOPH_1888 | Sm-like protein, partial |
| SLOPH_1889 | Small nuclear ribonucleoprotein |
| SLOPH_1896 | SNF2 domain containing protein |
| SLOPH_1897 | SNF2 family protein, partial |
| SLOPH_2558 | Tat binding protein 1-interacting protein |
| SLOPH_2709 | Thrombospondin type 3 repeat containing protein, partial |
| SLOPH_1992 | Transport protein particle complex subunit |
| SLOPH_1994 | Trehalase |
| SLOPH_2017 | UBA domain protein |
| SLOPH_2308 | Ubiquitin-like protein |
| SLOPH_2040 | UBP-type zinc finger protein |
| SLOPH_2537 | V-type ATPase subunit D |
| SLOPH_2048 | V-type H+-transporting ATPase subunit E |
| SLOPH_2066 | WD repeat domain protein |
| SLOPH_2605 | WD40 repeat protein, partial |
| SLOPH_2074 | WD40 repeat-containing protein |
| SLOPH_2637 | Zinc finger C3HC4 type protein |
