# Supplementary material for: The Genome of Spraguea lophii and the Basis of Host-Microsporidian Interactions
Source: PLoS Genet. 2013 Aug 22;9(8):e1003676. doi: 10.1371/journal.pgen.1003676 (PMC3749934; doi:10.1371/journal.pgen.1003676)
Supplement: Table S4 — List of genes identified from RNA transcripts alone that are not included in our genomic data. (DOCX) [file pgen.1003676.s008.docx]

**Table S4**: **A list of genes identified from RNA transcripts alone that are not included in our genomic data.**

| >MADS_box-containing_transcription_factor_(partial)_from_Contig_38077_c0_seq1 | ATGAATGACAACAGTAAAAATTACCACAATAAAGAGAATAGAAGAATAAATAACATGGATGATCATTATGAAGATAATTATCAATATTATACAGATCCATACACTGAAAAATACCAGCATTACGAAGAAATGGATGGGGATATAATTTACGAAGAACAAAATACAAAAGAGGAATACATCGACCATACCCATTCTTTATACGATTATCCATATGAACAAGAAAATGAAAAAAATACAAATACAGGACGTTTAAGAACAGGAAGAAAAAAAATAGAAATGACAAAAATACCACAAAGAAACAGAAGAAGTGTAACATTTTCAAAGAGAAAAAAAAGGATTAATGAAAAAAGCATATGA |
| --- | --- |
| >Non_LTR_retrotransposon_Contig_38326_c0_seq19 | TCAACAACCTTTTTCTTCTCTTCTGCGTTTATTATTTTTTTTTTGTTTTTTTTTGTTTTGAAAATTTTTTTCTGGATGAGGGATTCGAACCCGCCAGGCTTGGGGTTTGGTTACCACCCCACGACCACTGGGCCAGGCGGGACTAATCCTAACTACAGCTTTAGGATTTTTGTACAGTGCACTTATATACTGGAGCACTCTACCATGGAAACCCATGGCTTCTAGTTTGTACAACAGAGCACCATGTGGGACTCGGTCGTATGCCTTTGAGAAGTCTAGGAACAAGACGTATGTCTTCTCATTCCTTAGCTGTCTGCGCCGTAGGATT |
| >ENDOPLASMIC RETICULUM MEMBRANE PROTEIN DEGRADING MISFOLDED ER LUMINAL PROTEINS_from_Contig_18671_c0_seq1 | ATGATGGGCTTTATAGTTTTTAGAGCTTTTTATTTACCGTATATACCAGCAATAATAGGTTATATGACAAGCAATAAAATTCCTTTGGATGATATTTTAGGAATATTTGTAGGAAATAGTGTGTATTATATAAAATATATTCTTCCGAAAGCAGGTATAGAACTTTTTACTACTCCACAATGGATAAAGAAGATATTTGGCGAA |
| >Non_LTR_retrotransposon-encoded_reverse_transcriptase_from_transcript_Contig_38326_c0_seq13 | ATGTCTATCAATATCAATAAGTGTGGAGTGATGGCTTTTAATTCACAGTTGCAAGACGTGAACTGTATGTTTGGAACAGAGCGGGTACCATGTGTTGATACATATGTGTACCTAGGTGTGAATATTGGGAGTGACCTTAGCTACAAAGGTATGATAGAGAGGAATGTCCTCCAGGGCACCCGTGCCCTCTACACGCTAATGGCGCACTTTAAAAATCCAACTCCACCTCTCTTTACAAAGCTTATGTTAGTGAAGTCGGTTATACGACCTATCCTGACATATGGATCTGAAATCTGGGGCATGTCTACAGACCGAGTAAGGCCGGTGCAGCGGGTGTTAAATATGGCATTGCGTGTTGTACATGGGTAA |
| >thioredoxin peroxidase_from_transcript_Contig_37963_c0_seq1 | TTGAATGAAATAAGCACCCGAAGAGAGAAATTTTTAGAGTTAGACGCAGAAGTACTTTTCGTGTCATGTGATTCTATCTATTCGCATATTACTTGGACTATGATTGAAAAGCAGGAAAGAGGTGTTAGAGGTGTTCTTTGGCCTATGGTTTCTGATTATAAAAGAGAATTGTGTAAAGCATTAGGATTATTAGAGGAAGAAGGAAATGCAAGCAGGTCTACAATCATAACTAATAAAGATGGTATAATAAAATATATTTCAATGAATAGTCAAGAGATTGGAAGAAGTGTATCAGAAATACTGCGGATTTTAAATTTTGTAAAATATGATGATATGAATAGAATTAAAAAATTACAAAGATGTAATACAAAAGGACAA |
| >LTR_Retrotransposon_from_transcript_Contig_15185_c0_seq1 | TCGGTGAACTCCCAACTACTTTAAATGGTAATCGTTGGCTCCTTGTTGCTGTAGACTATGCCACCAACTGGCCCATTGCTCGTGCTCTCCCTGTCGCATCAACGGAAGCTGTTGCTGATTTCATTTATGAAGAAATAATGATGAAATTTGGTTGCCCCGTCGAAATTCTTACTGATCGTGGTGCTCTGCGTACCACCCTCGAACAAATGCTAAAGTCGAACGCTTTAATGGTGTG |
| >Amino_Acid/Auxin_Permease_(AAAP)_Family_from_transcript_Contig_38201_c0_seq2 | ATGCTGACGACAATGTTTGGAGTGGGTGTTCTATCACTCCCAGTAGCATTTATGAATGCAGGATGGCTTGTTTCAACTATATTGTTCGGTATTGTCGGAATTGTAACATTTATTTCATTATACTCAATTGCTGCAACAGCATATTATTCAAAATTAGAAGATCCTACATTTTTTTCAGTCTGCGCAAACGCCAATCCGGTTCTTGGATATTTGGTTGATATATTCGTAGCACTCAATGGATTTTTAGTTACCTCCATTTTCATATGCAATATTTCAAATTGGATCAAAGACAGTATTTTACCAACGATAAAAATCAATAATGCACCATTATATCTTGTGACAATCGGAGTACTAACAGTTCTTTTCTTACCTTCTATGATTGATAGTATTACTGTTCTTCAGATTGCTAGTTATCTATCTATTGGGTCTTTT |
| >Similar_to_hypothetical protein VCUG_01821_from_transcript_Contig_38725_c0_seq1 | ATGCTTTCTCAATCTGTCGTAGATTTTGTTGGAAATTTTATGTTCATTACTTTTCCTATCGTTGGATACATACCACAACTAATACGTAAGCGTATTCTTTTTGCTCCTATTCTATCTATTATGACTGTATGTTGTAATATGTTAAAATTGTTTTATTGGAAAGGAAATGATATGCCAGTGGCTATAGTATACCAAAGTTTATTGTTGATGATCTTTCATTTGATACTTCTTTATAATAATAAGAATGATTTATCTGCTGTAGAAGAAAAGATAATTGAAAAAAGTAAGATGAGTAATAATTATAAGAAATATGGATTATTTAAGCTATCTCTTTCTTTGATTGTTTCTTTGGTTATTTCATTGACAATTATTAATACAATGTTTTCTGGTGTATGTTTTATTTGGGGTCATTTAGCTATGGTTTTAGAATCTAGTGTAGGATTAGTGCAGTTGTTGATTTTAAATATGGATAATAGATATGAAATACCGGCTAATGATTTGTTTAAGATAGAAAAGAATTTTCCTAAGGAATTATATCTTTGTTGGGCATTGGGTGATATGCTAAAATTGATATGGATGTATCACATAGCATCTCCGTTGATATTCTATGTTTCTGTAGTATTTCAAATTGTTGTAGATTTATTTTTAGTTTTAGCCCATTAA |
| >Amino Acid/Auxin Permease (AAAP) Family_from_transcript_Contig_36990_c0_seq1 | TTGAAAGCAATGCTGGGAAGTGGTTTTTTACCAATGCCAACATTATATTATTGTTATGGAATAATTCCTACAACAATTTTATTGGTGCTTGGTGCTATTTGTGGAATGATCGGATTGAATTTATTTCTAACGCTTAATGCTTATTATGGTAAAAATTCGACGCTTTCTACACTCGCATATGAAATATGCCCAGTATTGAGATATGTCGCAGATATTGCAGTTTGTCTTAAATGTCTAATGGTGTCTCTAGCATATATAGTTCTTATTAAGAATCAGATTGATATTGTAGTAAAGGTTCTTAATTTACCTTTTAGTAGCAAAATACCGTTACTGATCTTTGTTCTTTTTGTTATTGTTATATCTTCTCTTGGAAAATTTGAT |
| >Similar_to_DNA_helicase_(partial)_from_transcript_Contig_23142_c0_seq1 | TCTAATATTTTTGAAGAAATAAAAATATCAACTGTAGATTCCTTTCAAGGGCAAGAAAAAGATATAATAGTAGTAACATTAGTAAGAAGTAATTGTTTATCAGAAATAGGATTCTTAAATGAAATTAGAAGAATTAATGTAGCAATAACCAGATGTAAATTAGGATTGATAATTATAGGAGATAGTAATAATTTCAAGGATAAATTTTATAAACAATTATTTTTATTTGTAAATACAAAAGGAATGGTATTAGATCCATCGATGCTCAAGGATTTATTAAATTAGATGGTGCATAATAATATGTGAGTGGAAGATTTTCTTCTCTTCTAATAGAAAATAAAAAAAAT |
| >DNA-directed RNA polymerase subunit B_from_transcript_Contig_32809_c0_seq1 | TTGGTAGATCCACATATAAAAAATTTTGAAAGTATATTTTGTGGAGATATAATGGAAAACATGATAGGAAATTTAGATTGTGTAGAGTTAAAAGATGATTTTAACAACGTATTAAGAATAGTGGTAGAAGATATAGAACTTCAAAAACCATATATTAAAAATTCAGATAATTCTTTTGATAGAAGATTACTTCCCATGGAATGTAGAGAAAGATTAATTACATACAAAGGTGGTGCTACCATACGTATAGCAGTAGTATTAAACGATAAAGTTATTGTAAGAGAAGATAAATCTATAGGAGGTATACCTATTATGGTAAATTCTACACTTTGTCATCTTAGAAATCAAGATTTTGTTAAATATAGAGAAGATGAAAATGAAACAGGTGGATATTTCATAATAAATGGATTGGAAAAAATATTTAGAAATCTGATAATTCAACGTAGAAATTATCCTATG |
| >uracil-DNA glycosylase_from_transcript_Contig_37209_c0_seq1 | CTGGACAATTTCTTATGTAATACATGGAAAGATGCTCTCAAAGAAGAATGTAAAAAACCATATTTCAATTTTATAAAGAAAACATTACATACAAAATCTACATTTTACCCACCGATAGACCGTATTTTTAGATTTTCCCATTATTTTAATCACCACGATACCAAGATTGTGATATTAGGTCAAGATCCGTATCATAATGAACACCAAGCAATGGGTTTATCATTTTCAGTTAATAAAGGTGTGAGAAC |
| >Contains_similarity_to_hypothetical protein ECU06_0590_at_5'_end_no_intact_ORF_from_transcript_Contig_39921_c0_seq1 | ATAATAATTATTTTTTATTACTAATATATTCTTTAGCTTTATTTTTAACATAATCTATATCACCATTATTCTTCTTTTCATATTCTAACCATAATTTATACCATTCTTTCATAATACCTATATTCCAATATATATTAGTTATTCTATTATATAAATAAGTAGAATATAATTTATTATTGTATTCTTCTTCTAAATATTTAGTAAATATTTTCTTTAAAATAATATTAGTAGTATGATTAGTGGTATGGTTAGGGTTAGTATGGGTTAGTGTATTAGTATGACTGTTAGTATTAGTTATAGTATTAGTAGTATGATTATCATACTTATTATTACCATCATTCCCATTATTACAATCATTACTATTGTTTATAAAATAATTTATTGTATTTTCAATTATCTCTCTTACTTCTATTGTATTATAATTTCTATATAATAATTTTATAATATAAATTATATATTCTTTATTAATATCTATTTCTTTTACAATAGAAGATGGTATAGTATTCTTAGAATTATTATTTATAATATATTTTATATTATTTATAAATGTATTTATATTATTAGTAGTAATATGATTGGTATGGTTGTTGGTAGTAGTGGTATTATTATTGGTAGTAGTGGTACTGTTAGTATTATTAGTAGTGGTATCTGTATTATTAGTATGGTTGTTATCATTATCATTACCATCACTCATATACTTATCGTTATTATCATTCATATCATTACTACCACTCATACACTTATTATCACTCATATGTTTATTACTATTAAATAATATATTATATTTATTTATAAAATTATATTCATTTGTATCTATTTCACTATCATCAGAAATATTTATTATTTTATCTTTTTTTTAATTCTTTTTTATCCTCTTTTCTATTTTCATTCTTATCTTTTTGTAATTCTTTTGTAAGAATAATTCTATCATTTTTTTCTTTTAATATTCTTAAATATAATATATCACCTATATTATATTTATTCATACTATTATTTATCATCTTTCTTATTACACTAGTAGTTTTATTGTTAGGGTTAGTATAGGTTAGTTTATTAGTATTTGTATTACTATTATCATACTTATTATTACTATTACCACTATATTTATTACTATACTTACTACTATTATTATTATCTTTATAAAGTATACCTCTTACTCCATTACTCTCTAATAAATAAAAACTATTATATTCTTTTATTATTTTATAATAATTAAAATTATAATTATATAATTTATTTTCTATAAAAT |
| >RNA-directed_DNA_polymerase,_Non_LTR_Retrotransposon_from_transcript_Contig_38326_c0_seq6 | ATGGGTTTCCATGGTAGAGTGCTCCAGTATATAAGTGCACTGTACAAAAATCCTAAAGCTGTAGTTAGGATTGGTGACACTGTATCAGAGAGCTTTGAGTATAGGATTGGTGTTAAACAAGGTTGTCCAGCGTCGCCAATTTTGTTTAACCTCTATATTAATGATCTCTTGGAAGATGTCACAGGTGTACAGGTAGATTCAAACCTACCACGTATACCTGGTCTTCTGTTTGCCGATGATGCTGTTATCTTGGCTGATTCTAAGCCTGACTTAGAGGACAGCATTAAAAGAATTGAGAAGTGGTGCCAGACTTGGAAGATGGCACTCAACATTGGGAAATGTGGAGTGATGGTAGTTGGAGGAGAAGACAGCAATGTCTTTGAATTCAATGGCAGTCCATTGCCAGCTGTGGATAGGTATACCTATCTCGGCATGCCATTTACTCAGACTCTCTGCCTTGATGCTATCATTGAGGATAGGAAAGAGAAAGCACGACGTGCTTATTTTTCAATGGCTGGATTTCTCAATAGGAAGAACGTTCCTGTACCAGTGAAGGTCCAGATAGTTAAGTCAGTACTTGTACCGATTGCAACTTATGGAGCTGAGCTGTACGGAATGTCAGCGTCTAGGGTCAGTCCCCTGCAGAGAGTAATGGACAACTCGGTTCGTACTATGCTTAGTGCACCAAGTAACTACTGCAGACGGGCTGCTTTTTCTGAACTTGGTCTTGAGTCTATTCAGACAAGAGTAGCTAAGTTGAGAACTAGAGGTTTTTTGAAGTGGAGAAATTCGAGTACTTGGATTTCTTCATTGATTGGAGCCTTCCCTAGAACAAGATGTGCCACTTGGGTCTCCGGATCCGCTAGATGGCTAAAAAGGTTTGGGCATGCAGTGCTTGAATCTTGTGTTCCTGATCATAAGAAAATGGAGTTAATCGCAGACGTGATCAGGAGCAGAGAGGTCTCTCGGGATAGGTCTGTCATAGGGAGGATCAGAGATGGTGGTAATATACGTCTGATCCAACCCAAGCTGTTAGCAGCTAAGAACCACCTCTATAGTGGATGGATGGCTTTGATACGTTTACGTATTGGAACATTTTATCTCTCTTATAGAATGGCTGTGTCAGGAAGGATTGACAGCAGATATAGACAGTCCTGTCCCTTCTGCGGTGGCGGAAGAGAGACGATGTTTCATCTCTTGTGGGTATGTCCTAGATGGGCTCTACAAAGAGAAGAAGCATTTAGTTGGATAAGGTCAGAGGACAATATTGTGCTACGAGCCTTATCTGCTAGCATTTCAAATCCACCAGAAGACAATCCAAGTCTTCTGGGGGTACTCTTAGGGGGAGAGTGGGAAGCTACTGGTCCGCAGCAATCAAAGGTAATTGTGGCTATGGCCACTTACCTAAAGTCCGTAATAAATCTAAGAAATAATTTGTTAAATGAGTATATAGAGAGCCACAGTAATCCTGATTCCGAACGTGGCCAGGGAACTAATTAG |
| >serine hydroxymethyltransferase_from_transcript_Contig_38154_c0_seq1 | CTGACCCAGAAGTATTTGAAATACTCAAAAAAGAAGAAAAAAAGACAAGAGGAAACAATAGAACTTATAGCCAGTGAAAATTATGTCTCAGTTGCTGTACTACAGGCCAATGGAAGTGTTTTTACGAACAAATATTCTGAAGGCGAAGTCGGAAGAAGATATTATGGTGGTAATGAATATATAGATGAAGTTGAAGGACTATGTAAAAAGCGCGCTCTTGAGCTTTTTAATTTAGATGAGGAAATATGGGGTGTTAATGTACAACCATACAGTGGTTCTGGTGCTAACTTTGCAGCTTATCTGGGAGCAGTAGGTGAAAATGGGAAAATCATGGGATTACATCTTTATCATGGTGGTCACTTAACTCATGGATTTGAAACCCCTAAAAGAAAGGTTTCTGCTACATCAATTTTCTTTAAATCACATCCATATTTTTTAAAGGATGATAAAATAGATTACGAGAGATTAGAAAAAGAAGCAAAAGAATTTAACCCTGATATGATTATAGCCGGTGCATCTGCATATCCATATGATTTTGACTATAAAAAATTACGCGAAATTGCAGGAGAAAAATATCTTTTGGTGGATATGGCACATATCAATGGTTTTGTAGCTACTGAAGTAATGAAAAATCCATTTGAATATGCTGATATTGTTACCTCAACAACTCATAAATTATTAAGAGGTCCTAGAGCAGGAATAATATTTTACAGAAAGAAAAAGGTGATTACTAAGAACAACACCCCTACAACAATTAATTTACAACAAAAGATCGACCAAGCAGTATTTCCAGGTCTTCAAGGAGGTCCTCACGAACATACAATAGCTGCATTAGCAGTTGCATTTAAGCAAGCAAATACTCCCGAATATAAAGAATATTGCAAACAAGTTCATCTTAACGCCAAAATAATGCTTAAAGAATTAGAAAATCGTGGAATACCATCATTTAACAAAGAAACAGATTCTCATTTATTTTTACCAGCCTTTGAAATCCCTATAGGTTACCATATTGAAAGAGTATGTGAATTAATTAACATTGCTCTCAATAGGAATTCCGTAGCAACGGATAAAAGTGCATTTGAACCATCAGCAATAAGAATAGGAACATGCGCTGTAACGACCAGAGGATTTAAAGAAAAAGAATGCATAAAGACAGTAGATTTTTTAATAAAAGCAATAGAAATAGCAAAGAAATATTATCATATCTCTAAAAATGATAAAAATAAATTTGAAGAATTAATTAAGGAAGATAAAGAGATACCTCTCTTAAAAACTGAAGTAATAAATTTCGTCAAAGGTTTTGCTTACCCAGTC |
| >DNAK-LIKE PROTEIN (HEAT SHOCK PROTEIN OF THE HSP70 FAMILY)_from_transcript_Contig_37149_c0_seq1 | TTGTTGTCAGTTACAAAAAAAAAGGAACCGTTTAAATTGGCTTTCTTTACTAATGAAGATATGATTGCAGAATTGGAAGTTAAAAAAGAAAATACAGAAGAATTGGAACATGTTAAAATAGATTTACAAATTTGTAACAAAGGTTTTATTGCTATTAATTCGATAAAAATAGACAATAATGATATTCCTTATGATTTTAGAACTTTAGGTTTTGAAGGTGGAATAAGAAAGCAAATAGAAGATTGTGAGAAAGTATATTTAAATTTAGAGAAAGAACAAGAGCAAATAGGAGAATTAAGATCTATTACAGAAAGATCTCTTCATATTTTATATGAAAGTTTATCATTCCCATGTTATAATTCATTATTTGAACGTAAATCTTTCGTAGAAGAAATTAAACAGGAATATTCATTTACTCCTGTAGGTAAAACATTGGCATGTGAAAAAAGTAACTCAGAAGAAGCATATAAGAAAATAGAAGATATTTATGATGCAATGAAAAAAATATTTGAACAAAGAAAAACCGATTTTGAAACAATAGTCATTCAAAGATTAGAATGTTTTACCAAAGATAAAAATGTATACACACCAACACTCTATAAGATGAAAGGTATGATACACATTCATAAGAAAAATTTAAAATTAGAATTTGATTTGAACAATATATTAAATTTGGATAATGTATTCAACCATGAGCAGATGGTAAAGGAAATAGAAACGATGGAGTGTGATATATTAAGAGAGATTAAAGAGAAGGAAGAAGAAGAAATAAGAAAAGCTGAAGAGGAGAAAAAAAGAGAAGAAGAACAGGAGAAGAAAGAAAAGGAGGCAAAAGAAGCAAAAAAC |
| >putative protein kinase domain-containing protein_from_transcript_Contig_38392_c0_seq1 | ATGCAATCAGAAGCATCTAAAGAGCATAAACCATTGATTCTCAAGAGGGATGACGCAGATCAACCAAAGCAGGAAGAATCCGAAAAACAAGGAAAGAAGGAGGATAAGCCGTTAAATCTTAGTAAGGAAAATGATTCTCCGAGTGATATACATTCAGAAGAATCTAAAGAACATAAACGTAAGGTAGGATTTGACACGGATAAAAATACGACACATAAAGTAGAAACTGATAAAGATAAAGCTTTAGATGAACCAGAAGAAGTAGTTAAACAGAGAGAAGAAATCTTACGAACAACAAATAAAGAAGAGAAAACATCTGCACCAGAACAACAACAAGAAGACAATAAAGAAAAAGAACCAAAAGCATCGGAACAAAAACCAGAAGCTGAAAAGAAAGAAGTAAATAATGCGACAGATGCATCTAACGATTTACAAAAGAATGAAGAAGAAAAAAAAACAGCAACAAATCCAAAAAAACAACAATCCAGCAAAAGATTTTTAGCCGCCGGATTTGGTTCTCCCACAAAAGATGATGAAAAGAATGTCCAACAGATGATAGATAAAAATTATGATCTACGAAAATTTGAAGGTGTCGATACGGGAAGATATGATGACACAGTAGAAGGTGAAATTAAAGAAGATAGAGACAACGATAATTCTGTTCTTGAAGGCTTTATTTTCAAAAGAAGATACTTTTTCTCTTGTTTCTGGCATGAAAGATATTTTGTTTTAACAAATGATGGAATTTTAAAATATTACGCCAATAAAAAATCCTCAAAGCGCAAGATAAACCTTGTAGACGTAATTGTAATGCACAGATTAAATAAACTAGATGATAAATATCCTTACAGAATTGTATTAAGATATGAAGATTCTGAAGATGAGCTAGCATTTAAGCAAAAGAAGCTTAGAGATCTTTGGGCATATCATCTCACTGGCATAAGAAATCAGGAAATAGAAAGAAATATTGAATCGGTTGATTCAAATGATGAATAA |
| >Similar_to_hypothetical_protein_EDEG_03949_from_transcript_Contig_38214_c0_seq1 | ATGGAAGATATATCATTATTTAAAATGCCTTCTGGAATACAGATATTAACCGTGTTAGCTTTAGCTTTTTCTACAGTGGTAATCATAGTGCTTGGATTTTTATATATTCCCGTCTTTCTTATGCATGCTTCTTTCTTAATAATGCCTGTAGCAAGTATTGCCATTCCGATATTTTTAAATATGCATGGTTCTGTTGTTGTAACAAGTATTGTTGGAGGAATTATAGGATTGTTATTTTATTTTTTTGTTGCTAGGAAGAATATAAAATTTTCAGCGAAAGTAGCGCAAGCCTCAACACGGATAATTCTAAAATATATGGGAGTGGTTATATTAATGATAACAATATTCATAAGTATTGCTATAACACTTGAATGTTGTTATCTTATTGCAGCAAAAAAACTCAAAGGAATTTGCGGCGGGAAATGGTATTCTAGGCATTGCGTTATTTTTTTCATTATCTTGGACTATGTTTGCTTTAATATATTTTATGAGAGTTTTTATTTCATCTGTTGTATGTCTAGAGATATTAACATATTCACATGTTTCTTCTATACTGACAGAGGCTCTATCTAA |
| >RNA-directed DNA polymerase, Non LTR Retrotransposon_from_transcript_Contig_38326_c0_seq5 | ATGGGTTTCCATGGTAGAGTGCTCCAGTATATAAGTGCACTGTACAAAAATCCTAAAGCTGTAGTTAGGATTGGTGACACTGTATCAGAGAGCTTTGAGTATAGGATTGGTGTTAAACAAGGTTGTCCAGCGTCGCCAATTTTGTTTAACCTCTATATTAATGATCTCTTGGAAGATGTCACAGGTGTACAGGTAGATTCAAACCTACCACGTATACCTGGTCTTCTGTTTGCCGATGATGCTGTTATCTTGGCTGATTCTAAGCCTGACTTAGAGGACAGCATTAAAAGAATTGAGAAGTGGTGCCAGACTTGGAAGATGGCACTCAACATTGGGAAATGTGGAGTGATGGTAGTTGGAGGAGAAGACAGCAATGTCTTTGAATTCAATGGCAGTCCATTGCCAGCTGTGGATAGGTATACCTATCTCGGCATGCCATTTACTCAGACTCTCTGCCTTGATGCTATCATTGAGGATAGGAAAGAGAAAGCACGACGTGCTTATTTTTCAATGGCTGGATTTCTCAATAGGAAGAACGTTCCTGTACCAGTGAAGGTCCAGATAGTTAAGTCAGTACTTGTACCGATTGCAACTTATGGAGCTGAGCTGTACGGAATGTCAGCGTCTAGGGTCAGTCCCCTGCAGAGAGTAATGGACAACTCGGTTCGTACTATGCTTAGTGCACCAAGTAACTACTGCAGACGGGCTGCTTTTTCTGAACTTGGTCTTGAGTCTATTCAGACAAGAGTAGCTAAGTTGAGAACTAGAGGTTTTTTGAAGTGGAGAAATTCGAGTACTTGGATTTCTTCATTGATTGGAGCCTTCCCTAGAACAAGATGTGCCACTTGGGTCTCCGGATCCGCTAGATGGCTAAAAAGGTTTGGGCATGCAGTGCTTGAATCTTGTGTTCCTGATCATAAGAAAATGGAGTTAATCGCAGACGTGATCAGGAGCAGAGAGGTCTCTCGGGATAGGTCTGTCATAGGGAGGATCAGAGATGGTGGTAATATACGTCTGATCCAACCCAAGCTGTTAGCAGCTAAGAACCACCTCTATAGTGGATGGATGGCTTTGATACGTTTACGTATTGGAACATTTTATCTCTCTTATAGAATGGCTGTGTCAGGAAGGATTGACAGCAGATATAGACAGTCCTGTCCCTTCTGCGGTGGCGGAAGAGAGACGATGTTTCATCTCTTGTGGGTATGTCCTAGATGGGCTCTACAAAGAGAAGAAGCATTTAGTTGGATAAGGTCAGAGGACAATATTGTCCTTCGAGCCTTATCTGCTAGCATTTCAAATCCACCAGAAGACAATCCAAGTCTTCTGGGGGTACTCTTAGGGGGAGAGTGGGGAGCTGCTGGTCCGCAGCAATCAAAGATACTTGTGGCTACGGCCACTTACCTAAAGTCCGTAATAAATCTAAGAAATAATTTGTTAAATGAGTATATAGAAAGCCACAGTAATCCTGATTCAGAACGTGGCCAGGGAACTAATTTTAATCTATAA |
| >Myb-like transcription factor_from_transcript_Contig_26791_c0_seq3 | ATGTTTTTTGACAATAGTAATATCAAAAGCGAAGATGAAGAAAATTCAATATCTACACACGATGAAGGAAGAATGAATAGAAAATGGAATATAGAAATGTGTGACGAAATGAATAATACAGAAAATATAAACAATAATTATGAAAATGTATTAAAAATGAAAAATGAGAAGAAGCATGGTCCAACATATATAAAAGGACCGTGGACACACGATGAAGACAATAAATTAAGAACATTGGTAAGTTTGTATTCTGGGAAAAATTGGTCGGATATAGGAAGATTGATGGGAACAAGAATAGGCAAACAGTGCAGAGAAAGATGGCATAACCATTTAAATCCAGGTATAAATAAACAACCATTCAGTTTAGAAGAAAATATTATGATATACAAACTTCATGATATTTTTGGAAATAAATGGTCAGAGATTAGTAAATACCTCCCAGGTAGAACAGACAATTCAATTAAAAATCAATGGAATTCTTCATTACAAAAAGAATATATCAGAAAAAGATCCATGTCAGCAGTATCCAACAAAGATATTGTTAAAATAGTAGAAAAATTATATTACAACAACGAAAATACCCAACCCACCAATCCTAACCCCGTACAACCAGAATTCAATCACCATAAACCCCATCTTCCAAAACCATCCACAAAACCATACAACCTCAAAAATCTAAACACACTAGCATACCATGCTTCAATACAAAGAATACCCATCAGAAAATCCATCGATGGATCAAATCTATTTCAAAGAAAAACACCCCAACCCAGCTTTCAACCATACACCAATAATAAAAAATCCATATTTCAATTTCAACCAGTACCAATGAATACCAAATTTGGACAACAACATAAAGTATTTTTTAATGACACACAGAAAAATGAAACAAAAGAACCATCTTCAGAAATCGAAAAAGAAAATAAACATTTTGATTCATGGAGTGAAGATGAAGAAAATCTAAAAAATATACTTACAGACATAGCTAGTAGTAAGAGTTAA |
