# Supplementary material for: The Genome of Spraguea lophii and the Basis of Host-Microsporidian Interactions
Source: PLoS Genet. 2013 Aug 22;9(8):e1003676. doi: 10.1371/journal.pgen.1003676 (PMC3749934; doi:10.1371/journal.pgen.1003676)
Supplement: Table S5 — A list of proteins identified from germinated and non-germinated S. lophii spores. (DOCX) [file pgen.1003676.s009.docx]

**Table S5**: **A list of proteins identified from germinated and non-germinated *S. lophii* spores.**

| **Germinated** | | **Non-germinated** | |
| --- | --- | --- | --- |
| ***S. lophii* ID** | **Annotation** | ***S. lophii* ID** | **Annotation** |
| SLOPH 10 | 20s proteasome subunit beta type- | SLOPH 10 | 20s proteasome subunit beta type-1 |
| SLOPH 101 | Actin | SLOPH 101 | Actin |
| SLOPH 108 | Adenylate kinase | SLOPH 108 | Adenylate kinase |
| SLOPH 1081 | Hypothetical | SLOPH 1081 | Hypothetical |
| SLOPH 11 | 26S protease regulatory subunit 6 | SLOPH 11 | 26S protease regulatory subunit 6 |
| SLOPH 1179 | Hypothetical | SLOPH 1179 | Hypothetical |
| SLOPH 12 | 26S protease regulatory subunit 6a | SLOPH 12 | 26S protease regulatory subunit 6a |
| SLOPH 1230 | Inorganic pyrophosphatase | SLOPH 1230 | Inorganic pyrophosphatase |
| SLOPH 1233 | Isoleucine tRNA synthetase | SLOPH 1233 | Isoleucine tRNA synthetase |
| SLOPH 13 | 26S protease regulatory subunit 8 | SLOPH 13 | 26S protease regulatory subunit 8 |
| SLOPH 1334 | Lysyl-tRNA synthetase | SLOPH 1334 | Lysyl-tRNA synthetase |
| SLOPH 136 | Argonaute protein | SLOPH 136 | Argonaute protein |
| SLOPH 1414 | Peptidase M1 | SLOPH 1414 | Peptidase M1 |
| SLOPH 1416 | Peptidase M18 | SLOPH 1416 | Peptidase M18 |
| SLOPH 1419 | Peptidyl-prolyl cis-trans isomerase | SLOPH 1419 | Peptidyl-prolyl cis-trans isomerase |
| SLOPH 143 | Asparaginyl-tRNA synthetase | SLOPH 143 | Asparaginyl-tRNA synthetase |
| SLOPH 1448 | Phosphomannomutase | SLOPH 1448 | Phosphomannomutase |
| SLOPH 1466 | Polar tube protein PTP2 | SLOPH 1466 | polar tube protein PTP2 |
| SLOPH 147 | Aspartyl-tRNA synthetase | SLOPH 147 | Aspartyl-tRNA synthetase |
| SLOPH 1488 | Prolyl-tRNA synthetase | SLOPH 1488 | Prolyl-tRNA synthetase |
| SLOPH 1540 | RAS GTPase | SLOPH 1540 | RAS GTPase |
| SLOPH 1572 | Ribosomal protein S26 | SLOPH 1572 | Ribosomal protein S26 |
| SLOPH 16 | 26S Proteasome core subunit-alpha-6 | SLOPH 16 | 26S Proteasome core subunit-alpha-6 |
| SLOPH 1662 | Hypothetical | SLOPH 1662 | Hypothetical |
| SLOPH 1788 | Hypothetical | SLOPH 1788 | Hypothetical |
| SLOPH 1854 | Hypothetical | SLOPH 1854 | Hypothetical |
| SLOPH 1923 | T complex protein 1 subunit beta | SLOPH 1923 | T complex protein 1 subunit beta |
| SLOPH 1925 | T-complex protein 1 subunit alpha | SLOPH 1925 | T-complex protein 1 subunit alpha |
| SLOPH 1945 | Thioredoxin | SLOPH 1945 | Thioredoxin |
| SLOPH 1976 | Transketolase | SLOPH 1976 | Transketolase |
| SLOPH 1979 | Translation elongation factor 1 alpha | SLOPH 1979 | Translation elongation factor 1 alpha |
| SLOPH 1980 | Translation elongation factor 2 | SLOPH 1980 | Translation elongation factor 2 |
| SLOPH 1995 | Trehalose-6-phosphate phosphatase | SLOPH 1995 | Trehalose-6-phosphate phosphatase |
| SLOPH 1996 | Triose phosphate isomerase | SLOPH 1996 | Triose phosphate isomerase |
| SLOPH 2039 | Ubiquitin/40s ribosomal protein S27a | SLOPH 2039 | Ubiquitin/40s ribosomal protein S27a fusion |
| SLOPH 204 | Chitin/polysaccharide deacetylase | SLOPH 204 | Chitin/polysaccharide deacetylase |
| SLOPH 2044 | UTP-glucose-1-phosphate uridylyltransferase | SLOPH 2044 | UTP-glucose-1-phosphate uridylyltransferase |
| SLOPH 2046 | V-type ATPase subunit A | SLOPH 2046 | V-type ATPase subunit A |
| SLOPH 2084 | Zinc finger protein | SLOPH 2084 | Zinc finger protein |
| SLOPH 2170 | Aldose reductase | SLOPH 2170 | Aldose reductase |
| SLOPH 2185 | Ubiquitin-activating enzyme E1 | SLOPH 2185 | Ubiquitin-activating enzyme E1 |
| SLOPH 2189 | 40S ribosomal protein S2 | SLOPH 2189 | 40S ribosomal protein S2 |
| SLOPH 2210 | 60S ribosomal protein L6 | SLOPH 2210 | 60S ribosomal protein L6 |
| SLOPH 2213 | Actin-depolymerizing factor | SLOPH 2213 | Actin-depolymerizing factor |
| SLOPH 2232 | Guanine nucleotide binding protein beta subunit | SLOPH 2232 | Guanine nucleotide binding protein beta subunit |
| SLOPH 2233 | Ribosomal protein L23 | SLOPH 2233 | Ribosomal protein L23 |
| SLOPH 2310 | Enolase | SLOPH 2310 | Enolase |
| SLOPH 2338 | Alpha trehalose-phosphate synthase | SLOPH 2338 | Alpha trehalose-phosphate synthase |
| SLOPH 2363 | Glucose-6-phosphate isomerase | SLOPH 2363 | Glucose-6-phosphate isomerase |
| SLOPH 2369 | 14-3-3 protein | SLOPH 2369 | 14-3-3 protein |
| SLOPH 24 | 26S proteosome subunit alpha-4 | SLOPH 24 | 26S proteosome subunit alpha-4 |
| SLOPH 25 | 40S ribosomal protein S0 | SLOPH 25 | 40S ribosomal protein S0 |
| SLOPH 2449 | Alpha-alpha-trehalase | SLOPH 2449 | Alpha-alpha-trehalase |
| SLOPH 255 | Cytosol aminopeptidase | SLOPH 255 | Cytosol aminopeptidase |
| SLOPH 2557 | Hypothetical | SLOPH 2557 | Hypothetical |
| SLOPH 263 | DEAD/DEAH box helicase | SLOPH 263 | DEAD/DEAH box helicase |
| SLOPH 2653 | metallopeptidase M24 | SLOPH 2653 | Metallopeptidase M24 |
| SLOPH 28 | 40S ribosomal protein S12 | SLOPH 28 | 40S ribosomal protein S12 |
| SLOPH 33 | 40S ribosomal protein S18 | SLOPH 33 | 40S ribosomal protein S18 |
| SLOPH 346 | Eukaryotic translation initiation factor 4A | SLOPH 346 | Eukaryotic translation initiation factor 4A |
| SLOPH 348 | Eukaryotic translation initiation factor 6 | SLOPH 348 | Eukaryotic translation initiation factor 6 |
| SLOPH 377 | Fructose biphosphate aldolase B | SLOPH 377 | Fructose biphosphate aldolase B |
| SLOPH 38 | 40S ribosomal protein S27 | SLOPH 38 | 40S ribosomal protein S27 |
| SLOPH 399 | Glyceraldehyde-3-phosphate dehydrogenase | SLOPH 399 | Glyceraldehyde-3-phosphate dehydrogenase |
| SLOPH 40 | 40S ribosomal protein S3 | SLOPH 40 | 40S ribosomal protein S3 |
| SLOPH 403 | Glycosyl transferase | SLOPH 403 | Glycosyl transferase |
| SLOPH 409 | GTP binding protein | SLOPH 409 | GTP binding protein |
| SLOPH 416 | Heat shock protein 70 | SLOPH 416 | Heat shock protein 70 |
| SLOPH 417 | Heat shock protein 90 | SLOPH 417 | Heat shock protein 90 |
| SLOPH 418 | Heat shock protein 90 | SLOPH 418 | Heat shock protein 90 |
| SLOPH 43 | 40S ribosomal protein S4 | SLOPH 43 | 40S ribosomal protein S4 |
| SLOPH 433 | Histone H4 | SLOPH 433 | Histone H4 |
| SLOPH 449 | Hsp70 protein | SLOPH 449 | Hsp70 protein |
| SLOPH 45 | 40S ribosomal protein S6 | SLOPH 45 | 40S ribosomal protein S6 |
| SLOPH 452 | Hydroxyacylglutathione hydrolase | SLOPH 452 | Hydroxyacylglutathione hydrolase |
| SLOPH 477 | Hypothetical | SLOPH 477 | Hypothetical |
| SLOPH 50 | 6-phosphogluconate dehydrogenase | SLOPH 50 | 6-phosphogluconate dehydrogenase |
| SLOPH 53 | 60S acidic ribosomal protein P0 | SLOPH 53 | 60S acidic ribosomal protein |
| SLOPH 54 | 60S acidic ribosomal protein P2 | SLOPH 54 | 60S acidic ribosomal protein P2 |
| SLOPH 55 | 60S ribosomal protein L10a | SLOPH 55 | 60S ribosomal protein L10a |
| SLOPH 57 | 60S ribosomal protein L12 | SLOPH 57 | 60S ribosomal protein L12 |
| SLOPH 577 | Hypothetical | SLOPH 577 | Hypothetical |
| SLOPH 59 | 60S ribosomal protein L18 | SLOPH 59 | 60S ribosomal protein L18 |
| SLOPH 596 | Hypothetical | SLOPH 596 | Hypothetical |
| SLOPH 6 | 20S proteasome component | SLOPH 6 | 20S proteasome component |
| SLOPH 607 | Hypothetical | SLOPH 607 | Hypothetical |
| SLOPH 61 | 60S ribosomal protein L2 | SLOPH 61 | 60S ribosomal protein L2 |
| SLOPH 62 | 60s ribosomal protein L21 | SLOPH 62 | 60s ribosomal protein L21 |
| SLOPH 63 | 60S ribosomal protein L22 | SLOPH 63 | 60S ribosomal protein L22 |
| SLOPH 655 | Hypothetical | SLOPH 655 | Hypothetical |
| SLOPH 66 | 60S ribosomal protein L27 | SLOPH 66 | 60S ribosomal protein L27 |
| SLOPH 68 | 60S ribosomal protein L3 | SLOPH 68 | 60S ribosomal protein L3 |
| SLOPH 69 | 60S ribosomal protein L3 | SLOPH 69 | 60S ribosomal protein L3 |
| SLOPH 691 | Hypothetical | SLOPH 691 | Hypothetical |
| SLOPH 7 | 20S proteasome component beta 3 | SLOPH 7 | 20S proteasome component beta 3 |
| SLOPH 723 | Hypothetical | SLOPH 723 | Hypothetical |
| SLOPH 73 | 60S ribosomal protein L4 | SLOPH 73 | 60S ribosomal protein L4 |
| SLOPH 749 | Hypothetical | SLOPH 749 | Hypothetical |
| SLOPH 75 | 60S ribosomal protein L5 | SLOPH 75 | 60S ribosomal protein L5 |
| SLOPH 78 | 60S ribosomal protein L8 | SLOPH 78 | 60S ribosomal protein L8 |
| SLOPH 82 | AAA ATPase | SLOPH 82 | AAA ATPase |
| SLOPH 888 | Hypothetical | SLOPH 888 | Hypothetical |
| SLOPH 99 | Acetyl-coenzyme A synthetase | SLOPH 99 | Acetyl-coenzyme A synthetase |
| SLOPH 1386 | NAD-dependent glycerol-3-phosphate dehydrogenase | SLOPH 1386 | NAD-dependent glycerol-3-phosphate dehydrogenase |
| SLOPH 139 | Arsenite-transporting ATPase | SLOPH 139 | Arsenite-transporting ATPase |
| *SLOPH 1395* | *Nonsense-mediated mRNA decay protein* | *SLOPH 1465* | *Polar tube protein 3* |
| *SLOPH 1429* | *Phosfructokinase* | *SLOPH 1490* | *Proteasome subunit beta* |
| *SLOPH 1437* | *Phosphoglycerate kinase* | *SLOPH 1539* | *RAN-specific GTPase activating protein* |
| *SLOPH 1438* | *Phosphoglyceromutase* | *SLOPH 1566* | *Ribosomal protein L15* |
| *SLOPH 1491* | *Proteasome subunit beta type 2* | *SLOPH 1628* | *Serine/threonine protein kinase* |
| *SLOPH 1517* | *Protein transport protein SEC23* | *SLOPH 1766* | *Hypothetical* |
| *SLOPH 1567* | *Ribosomal protein L34* | *SLOPH 18* | *26s proteasome regulatory subunit* |
| *SLOPH 1591* | *RNA recognition motif domain cotaining protein* | *SLOPH 1884* | *Spore wall protein 12* |
| *SLOPH 1608* | *SEC24-related protein* | *SLOPH 1949* | *Threonyl-tRNA synthetase* |
| *SLOPH 1639* | *Seryl-tRNA synthetase* | *SLOPH 2164* | *Glucosamine 6-phosphate N-acetyltransferase* |
| *SLOPH 1717* | *Hypothetical* | *SLOPH 2241* | *Hypothetical* |
| *SLOPH 1926* | *T-complex protein 1 subunit delta* | *SLOPH 2251* | *Peroxiredoxin* |
| *SLOPH 1928* | *T-complex protein 10* | *SLOPH 2268* | *NADH-cytochrome b5 reductase* |
| *SLOPH 1957* | *Transcription elongation complex subunit (Cdc68)* | *SLOPH 230* | *Hypothetical* |
| *SLOPH 1977* | *Transketolase* | *SLOPH 2344* | *Hypothetical* |
| *SLOPH 2050* | *Vacuolar ATP synthase subunit B* | *SLOPH 2373* | *Hypothetical* |
| *SLOPH 2178* | *Hypothetical* | *SLOPH 2431* | *20S proteasome alpha type-1 subunit* |
| *SLOPH 2217* | *WD-40 repeat-containing protein* | *SLOPH 2448* | *Hypothetical* |
| *SLOPH 2267* | *Hypothetical* | *SLOPH 34* | *40S Ribosomal protein S19* |
| *SLOPH 2334* | *T-complex protein 1 epsilon subunit* | *SLOPH 35* | *40S ribosomal protein S20* |
| *SLOPH 2418* | *T-complex protein 1 zeta subunit* | *SLOPH 392* | *Glucose-6-phosphate isomerase* |
| *SLOPH 2665* | *Arginine/alanine aminopeptidase* | *SLOPH 479* | *Hypothetical* |
| *SLOPH 2673* | *Proteasome B-type subunit* | *SLOPH 48* | *40S ribosomal protein S9* |
| *SLOPH 2686* | *Serine hydroxymethyltransferase* | *SLOPH 49* | *5'- 3'exoribonuclease* |
| *SLOPH 27* | *40S ribosomal protein S11* | *SLOPH 535* | *Hypothetical* |
| *SLOPH 29* | *40S ribosomal protein S13* | *SLOPH 548* | *Hypothetical* |
| *SLOPH 30* | *40S ribosomal protein S14* | *SLOPH 689* | *Hypothetical* |
| *SLOPH 31* | *40S ribosomal protein S15A* | *SLOPH 76* | *60S ribosomal protein L7* |
| *SLOPH 37* | *40s ribosomal protein s24* | *SLOPH 762* | *Hypothetical* |
| *SLOPH 393* | *Glutaminyl-tRNA synthetase* | *SLOPH 835* | *Hypothetical* |
| *SLOPH 394* | *Glutaminyl-tRNA synthetase* | *SLOPH 854* | *Hypothetical* |
| *SLOPH 415* | *Heat shock protein 101* | *SLOPH 887* | *Hypothetical* |
| *SLOPH 42* | *40S ribosomal protein S3A* | *SLOPH 962* | *Hypothetical* |
| *SLOPH 424* | *Histidyl-tRNA synthetase* | *SLOPH 1073* | *Hypothetical* |
| *SLOPH 44* | *40S ribosomal protein S5* | *SLOPH 1175* | *Hypothetical* |
| *SLOPH 473* | *Hypothetical* | *SLOPH 1347* | *Methionine aminopeptidase* |
| *SLOPH 580* | *Hypothetical* |  |  |
| *SLOPH 79* | *60S ribosomal protein L9* |  |  |

Three biological replicates were pooled and quality filtered giving a final list of 143 germinated and 141 non-germinated proteins. Comparative analysis revealed minimal difference between the two life cycle stages. Italicized IDs indicate different proteins retrieved between the two samples.
