# Supplementary material for: The Genome of Spraguea lophii and the Basis of Host-Microsporidian Interactions
Source: PLoS Genet. 2013 Aug 22;9(8):e1003676. doi: 10.1371/journal.pgen.1003676 (PMC3749934; doi:10.1371/journal.pgen.1003676)
Supplement: Table S7 — Expanded gene families that have proteins identified in our germination secretome. (DOCX) [file pgen.1003676.s011.docx]

**Table S7: Expanded gene families that have proteins identified in our germination secretome (starred). These were used as bait for a BLASTP search (e<1x10-5 cutoff) and were aligned with Clustal Omega:**

***S. lophii* gene family 1:**

*SLOPH_580_Contig_15878 MDLFINNNTKLFKHSTVSKHMLLNTKKNIKYILKSLLLSTIMLLCLKLLNXTKKNIKYIL

SLOPH_2487_Contig_11389 ------------------------------------------------------------

SLOPH_1048_Contig_6469 ----------------------MNSIHTTNHFYTNILNLNNTIIQHKIYNRDNFSIR---

SLOPH_956_Contig_5046 ------------------------------------------------------------

SLOPH_2571_Contig_7208 ------------------------------------------------------------

SLOPH_580_Contig_15878 KASFLSTIMLLCLK---LLNYTFIPTPYAKCIFTDKQRIEDLYNNHDSTITNLNFSRDDE

SLOPH_2487_Contig_11389 ---------------------TFIPTPYAKCIFTDKQRIEDLYNNHDSSITNLNFSRDDE

SLOPH_1048_Contig_6469 ------FSLIFCNTLCYFLFYLNIRMFYINCTSFNKERIIGLYENNGIALTNIRFSKYDE

SLOPH_956_Contig_5046 ------------------------------------------------------------

SLOPH_2571_Contig_7208 ------------------------------------------------------------

SLOPH_580_Contig_15878 IYLDYFDFNLHVDYMLPDSLNRPELLELFKEYCEIVKYPADELSQYENAINESITTENLL

SLOPH_2487_Contig_11389 IYLGYLGFNLHVDYMLPDSLNRPELLELFKEYCEIVKYPADELSQYENAINESVIIEKLL

SLOPH_1048_Contig_6469 IYIEHKEFNLHIDYLLPDFLDIPSVQRLYSKYKDHISFSKHISKEFELCINNSTTTKELY

SLOPH_956_Contig_5046 ------------------------------------------------------------

SLOPH_2571_Contig_7208 ------------------------------------------------------------

SLOPH_580_Contig_15878 ENLISYFAKYHEILSSKIKYIYNIMEKYHGEYGKIGDPNKIT---IEDFYYPSRPGDRLI

SLOPH_2487_Contig_11389 ENLINYFAKYHEILSSKIKYIYNIIKEYHGGHDRIGDPNRIT---IEDFYYPATPTCRFI

SLOPH_1048_Contig_6469 CNLIAYFAKSHTILNSKVREIYFRMKMYFYRTNLLPD---NCLITKEHFTHMIYYRKLKR

SLOPH_956_Contig_5046 -----------------------------------------------------------M

SLOPH_2571_Contig_7208 -----------------VREIYFRMKVYFDPTNLLPDPDNNCLITKEHFAHMIHYRKLKG

SLOPH_580_Contig_15878 FEKFCEESDKYIVPISILLQKIETTEIYTQILGYELIKVKAYSNIGSVPHLHNSLEMNIK

SLOPH_2487_Contig_11389 FEKFCEELDKYIVPISILLQKIETTKIYTQILGYE-------------------------

SLOPH_1048_Contig_6469 REDFIIVLKEYLLPLSIIMQKVETTQLYTRIIGYELLYLNIITQIQMVPHLFDIFHENIK

SLOPH_956_Contig_5046 NDNFNSDLRKYILPLSILLQKSQITEIYTRIIGYELLHCETMKIISDIPHLYYTFINYIK

SLOPH_2571_Contig_7208 RDDFSIILKEDLLPLFILMQKIETTQLYTRIIGYELLYLNIITQIQIIPHLFNIFHENIK

:.* : ::*: *::** : *::**:*:***

SLOPH_580_Contig_15878 NTFVQAIQTLTRIAALSVDMQSKHFEFRPSQNYFSLLKIHFFILRVKDILLCFCLNKENP

SLOPH_2487_Contig_11389 ------------------------------------------------------------

SLOPH_1048_Contig_6469 EEFTDSLQWLDNLFNIILHIGVEGRVKSRGFYSFSLIMLHKTFIKMKHFLKYFYTSQENI

SLOPH_956_Contig_5046 KEFIDGVVNLDKIINAMFRLDKKQTKRANSPHYLSLMKLHNTLIDMKQFLVPFYKNKNNP

SLOPH_2571_Contig_7208 KEFIDSLKCLGNIFSVILHVGIEGRITFRDCYSFSLIPVHQTFIKMKHFSKYFYIGQENI

SLOPH_580_Contig_15878 KFEPMLKKYMFSDLNSFISY-TIYDMYESVKTIFDLSDKTDEEFKK--RTIHKIIKLFSK

SLOPH_2487_Contig_11389 ------------------------------------------------------------

SLOPH_1048_Contig_6469 NLSEILGIPLKYIRSDFFKNNLLKNLLDAMSILYSAKEDENDISTNMTNKID-DIRKWP-

SLOPH_956_Contig_5046 DFKQILIRENTSNILNYIIHFTFKNVSDIMWILRDLENTK-SLYKNIDTKFKIIILTWTS

SLOPH_2571_Contig_7208 NLSEILRIPFYYNRSDFFKNDLCKNLLDAIWILYSENEGENDISTNMNNKIE-NIRKWP-

SLOPH_580_Contig_15878 TSLNIIDIYSLDSIELLNNILV----DVDDL-------

SLOPH_2487_Contig_11389 --------------------------------------

SLOPH_1048_Contig_6469 -DIHIKDLNLEKAKKIF-DMLFDKRFTSHDMEKEIFSK

SLOPH_956_Contig_5046 KSIEIKNLNIIKMKNFF-DMTFDEKYTVYDMKKKLFE-

SLOPH_2571_Contig_7208 -DIHIKDLNLEKAKKIF-DMLFDKRFTSHDMEKEIFSK

***S. lophii* gene family 2:**

*SLOPH_607_Contig_16670 -----------------------MLNRNRIDFIYGNEKNFHFF--------IKLYKNIKL

SLOPH_961_Contig_5050 MYLFFIYFDKKNKYLIFFLMGAEMLSRNRIDFIYENEKNFDFF--------IKLYKNIKL

SLOPH_507_Contig_11452 -----------------------MLSINKLNFIYEKEKNLNFF--------IKIYKSIKL

SLOPH_926_Contig_4765 -----------------------ML----------NRNESNFL--------IKLYRSIRL

SLOPH_786_Contig_2784 ------------------------------------------------------------

SLOPH_2513_Contig_15665 -----------------------MLNRNESDFLCRKEKNSNFF--------IKLHKYIKL

SLOPH_988_Contig_5460 -----------------------MINRNKISLIYENEKNFNLL--------IKLYKSVRL

SLOPH_1056_Contig_6587 -----------------------MLTRNEYDFFCREEKNFNFF--------IKLYRNIKL

SLOPH_2587_Contig_9268 ------------------------------------------------------------

SLOPH_635_Contig_17914 ------------------------------------------------------------

SLOPH_880_Contig_4187 -----------------------MLNREDFHTIYKEEIKTNTYVNLVAIKFIKISENNIF

SLOPH_607_Contig_16670 LGIIKIRKLPVFCLLYFMTIQSSTISNHGREANIEDVLIKIXEVNGTEKEDNGVVSNLDY

SLOPH_961_Contig_5050 LGMIKIRKLAVFCLIYFMNIQASNISNRGQETNIEDVLIKIQRKNGTKKEGEGIICDLGY

SLOPH_507_Contig_11452 LNMIRIKKLPILYLIYFMNMQASNIINRGKKANIKDVLIKIREKNRTERKGYEIIDNLGY

SLOPH_926_Contig_4765 LTMIRIKKLPVLCLISFINIQVSNINVCAEEANVKDILIRIKDKNEVEIKDNGIINNLGY

SLOPH_786_Contig_2784 ------------------------------------------------------------

SLOPH_2513_Contig_15665 LIMIRIKKLPVLCLIYFMNIQASNVSNLGEEANVKDVLIKIQTKNGIEIQSNGITNKLGY

SLOPH_988_Contig_5460 SLLIRIRKLPVFCLVYFMNIQAGNINNHSEEANIKDVLIKIQEEDGIEKEGNGIIFNLDY

SLOPH_1056_Contig_6587 LIMIRIKKLPVLCLISFMNIQASNISACGEEASVKDVLIKIRKKNGTEIRGNGIINKLGY

SLOPH_2587_Contig_9268 ------------------------------------------------------------

SLOPH_635_Contig_17914 -----------------MNIQASNINACGEETNVEDVLIKIKKGNKPEIQANGIINNLDH

SLOPH_880_Contig_4187 SYIVRISKLFSICFVFLLKVNSASISEEGKRARVQNITLELKKPNEVDNISNGIIENINI

SLOPH_607_Contig_16670 KAKLD-D-GENTEFTINSLEYSGIRIANGNCDADYGFSNLKLNDQGKLEGKNMNGNDIPG

SLOPH_961_Contig_5050 KARSA-C-GKSTELTINSLEYSGVKISNITPNGGRSFANLKLNDEGKLEGKGKNDINVSG

SLOPH_507_Contig_11452 SARLR-D-NKKIRFKINSLKYNGIRIDNTHNKTHHSFSKLKLNDKGKLEGKNKNGNDIPE

SLOPH_926_Contig_4765 DAKLK-Y-GKRTKFTINNLEYNGIRIANKNCNNYYGFSNLKLNGEGKLKGKYKNGDDVPE

SLOPH_786_Contig_2784 --------------------------INKDFNDSYAFSNLELNDQEKLEGTDKNGNYIPE

SLOPH_2513_Contig_15665 KVKLKNK-QMKLKIKIKRLEFSGVDVVIRKSMEYVYFSKLEINKYGMIEGVDQNGNMITE

SLOPH_988_Contig_5460 GAVFHED-DKQTRFTINNLEYNGIGIINKDFNDSY-------------------------

SLOPH_1056_Contig_6587 KAKLNNK-QMTCDIKIKRLEFSGVDVVIRKGMSYVYFSKLGINKDGMIEGVDENGNTITE

SLOPH_2587_Contig_9268 ----------------------------------------------------VNSNIILE

SLOPH_635_Contig_17914 NVKIKNI---KAQCKIKTLEFSGVDVVVRKDMDYVYFSRLKINKDGIIGYGK--EKMITE

SLOPH_880_Contig_4187 NAITNHSKLDGLILDILSFEYSGVEVFGKDVDRCIPLPNVESNTKGELEGKDISDNIIPE

SLOPH_607_Contig_16670 NEILIYYLLFYANITIGDNNINTFIRILPSN-HPVYNNV--D-IYEVKPS-HLWVYLRVD

SLOPH_961_Contig_5050 NEIMIVDILFYANITIDNNIINTFIDIMPED-YLSYNDIDND-IYEMKPY-QIWVYLRVD

SLOPH_507_Contig_11452 NKIMISYILFYANITIGNNTINTFIHIIPQQ-YIIYNNVVNT-TFEMEIY-DLRVCLDVD

SLOPH_926_Contig_4765 NKIVKYDMLFYANVTIGNKIINTFIRIIPEI-ILHIIILL---VILLKGNHIAYVFI---

SLOPH_786_Contig_2784 NEILIDYILFYANITICNKVINTFIRIQIPN-QLIYNNTVNN-IFEVKPK-CLYIYLDLD

SLOPH_2513_Contig_15665 NMLLTETITFKATIMIKDDEYETYLYFKYSD-QFKYNDAISS-EIPIDTR-YAIIYLPSK

SLOPH_988_Contig_5460 ------------------------------------------------------------

SLOPH_1056_Contig_6587 KELLTETITFKATI----------------------------------------------

SLOPH_2587_Contig_9268 NKMMITNILFYANITINSTTINTVINVKLTE-NIAYNNIYND-IYKVKLG-YFYIYLDND

SLOPH_635_Contig_17914 NXHQRKHMKKYTENHLKNHT-EKYMKKYTKN-HL-----KNH-TVK--------------

SLOPH_880_Contig_4187 DEMYINTIIFKIKMKVNNHIYETYMLLIDMNKNYKYIDVTDNFTLTFKTF-VHHVFLPPK

SLOPH_607_Contig_16670 SNGIHSGTHYDCTAKCYESEFFK---NCKLFTLNNKIISIKISNFKNAGYIYYPNINSQK

SLOPH_961_Contig_5050 SNGGYSGKNYDDMAGLYKSKFFK---NYKLFTFDNKISSIEISNFKNAGYIYYPNINSQK

SLOPH_507_Contig_11452 SNGVYSNKDYDYIAGSTYKKSSL---NYNSFILANKPSSIKISNFEKTGYIYYFNSRSEK

SLOPH_926_Contig_4765 ------------------------------------------------------------

SLOPH_786_Contig_2784 LNDVYYSRNYNYTDGLVTLSFFT---ECKQFTLDNKPNSIKISKFKNAGYIYYYNTKSNK

SLOPH_2513_Contig_15665 IKNKYFPVEFDNIKGCYIDNLGNILKDKYIIEFTDKNWDMKISNIK--------------

SLOPH_988_Contig_5460 ------------------------------------------------------------

SLOPH_1056_Contig_6587 ------------------------------------------------------------

SLOPH_2587_Contig_9268 SNNVYSSIDYDYMVGLNILKYFK---NFKVFTFDNKIISIEMSNFKNAGYXIMILMK---

SLOPH_635_Contig_17914 -----YRKKYKKK-----------------------------------------------

SLOPH_880_Contig_4187 IPNHNFYVRFDNSRGEYIDQNNI---KCYSYKLSFKCESMSISNIHKNGYIYFYDKPREK

SLOPH_607_Contig_16670 FERITVKPETSSELDKKI------------------------------------------

SLOPH_961_Contig_5050 FERIAVKPETSSERDING------------------------------------------

SLOPH_507_Contig_11452 LERITLKPEATFNESEGERTLQPEAT-----------------------------FNESE

SLOPH_926_Contig_4765 ------------------------------------------------------------

SLOPH_786_Contig_2784 LRRITVKPETTSDRKDVDEPTNQETSSQRDVKEIINQEITPDKKEIEETKNQKTIFKRCK

SLOPH_2513_Contig_15665 ------------------------------------------------------------

SLOPH_988_Contig_5460 ------------------------------------------------------------

SLOPH_1056_Contig_6587 ------------------------------------------------------------

SLOPH_2587_Contig_9268 ------------------------------------------------------------

SLOPH_635_Contig_17914 ------------------------------------------------------------

SLOPH_880_Contig_4187 RQRFIPVQITQ-------EPLQNKFEM---------------------------------

SLOPH_607_Contig_16670 -----------------------------------------------------------Q

SLOPH_961_Contig_5050 -----------------------------------------------------------T

SLOPH_507_Contig_11452 GERTL------------------------Q-----------------PEATFNENEGEGI

SLOPH_926_Contig_4765 ------------------------------------------------------------

SLOPH_786_Contig_2784 GNNNQEITSQRDVKEIINSEIISDKNKTDEPTKQETISDIKEIEETTKPETIPKRDVKEI

SLOPH_2513_Contig_15665 ------------------------------------------------------------

SLOPH_988_Contig_5460 ------------------------------------------------------------

SLOPH_1056_Contig_6587 ------------------------------------------------------------

SLOPH_2587_Contig_9268 ------------------------------------------------------------

SLOPH_635_Contig_17914 ------------------------------------------------------------

SLOPH_880_Contig_4187 ------------------------------------------------------------

SLOPH_607_Contig_16670 DNVKRRLHGKKKRGNKARNHF----------------------

SLOPH_961_Contig_5050 TKPETISDGKEKEVIRNLGSLLK--------------------

SLOPH_507_Contig_11452 TKSETSSDGKRKEVASKNNIKIGNTMIILISIQIIFWLFIQIY

SLOPH_926_Contig_4765 -------------------------------------------

SLOPH_786_Contig_2784 TNSEIISDKKETEETKKNNIKIGNIIITLISVQIIFWLFMQIY

SLOPH_2513_Contig_15665 -------------------------------------------

SLOPH_988_Contig_5460 -------------------------------------------

SLOPH_1056_Contig_6587 -------------------------------------------

SLOPH_2587_Contig_9268 -------------------------------------------

SLOPH_635_Contig_17914 -------------------------------------------

SLOPH_880_Contig_4187 -------------------------KHTMILPSMIIWVIMLLY

***S. lophii* gene family 3:**

*SLOPH_1121_Contig_782 --------MIFN----KFVYLMSLNYIFLSRIVCATYE----EYEFPEEKIENVKNIWNN

SLOPH_1122_Contig_782 ----------------------MRLYLFLSKILCAYKV----YEEYEENNIENIKEIWDD

SLOPH_1080_Contig_7159 MSLNALYIKLFKKLSMYYLYLFQINIIFL-LIKCSDNETGTNDKSLSFEMIENVKNIWNN

SLOPH_652_Contig_1894 MSLKYI-NTGENTISMYYFYILKLL----TISRCSSLNGEYSNKINYDEKIDYLKNLWNE

*: : *: :*::*::

SLOPH_1121_Contig_782 DLQTQTYLTYEGDNFWLNDTEFMVDGIGISLTFDHNE--LFEICKSTNQNDIKRSFDDMN

SLOPH_1122_Contig_782 NTHIITYISCENGEFWLNDTEFIRKGVALSID--YKE--FFKNSKYIDKESLVKSFNDMI

SLOPH_1080_Contig_7159 DAITTTNLFYESNNFWLYDTEFNREEFGINIELEDSY--Y-DISD-KSKDEIENVLKYAK

SLOPH_652_Contig_1894 NEATTTYLSYEGNDFWLNETGYNRENIFYDINLREINAYFKNECK----NILNIKIQLIE

: * : * :*** :* : . . .: . .. : : :.

SLOPH_1121_Contig_782 NYKNEILNSDNIIDLVEALTNYNVFRIEYLKYVVNEFNNES-------NLKKCAFKKAGT

SLOPH_1122_Contig_782 YWKNCILSSDNILELGEALINYNVFRIEYLEYIINELERLK-------YAKENEFNKNRA

SLOPH_1080_Contig_7159 TLEDNIFKSKDIIQLLESLCEYNVYRIKYYELLVQAHGDIINDFYKNLNAKSSSIHRENP

SLOPH_652_Contig_1894 EQQDKFMKSEDLFELFENVYEYNESRLKYLDEVLEFYKNDQ--------ENGIEFTYDNL

:: ::.*.::::* * : :** *::* . ::: : :

SLOPH_1121_Contig_782 KKLTEMGGYYYLNIDEQKINLNNMSYGEEEYKLSDIARCNLYKSEIMRFKLAGEYPKIRI

SLOPH_1122_Contig_782 NGIYENGKSYYFYKTDKFGKLKHISGCEYEEKNLAIVKYNFFRSKIMSSKLAGEYPKIKL

SLOPH_1080_Contig_7159 E-----RGY----NTQNINGESSESYKKKNSKIFYITKYNLFRSKNMQLKLVAEYPKVKP

SLOPH_652_Contig_1894 YTYSEML------GKTQISVIAEQTYEEKQTVTVNILRFNLYRSKMMQETLINELDNLKT

: : : : * : *:::*: * .* * :::

SLOPH_1121_Contig_782 SSMQIIPHITSTFNNYMIAFTHCDEYIGYLKELRECYGSIRRNKLSTPEEKIYSLFWFIL

SLOPH_1122_Contig_782 LSIQNIPHVAYQFNNYVD-----SNYEEYKKETTNCLEIIKNSDLTTPAEWFYVNFEHSF

SLOPH_1080_Contig_7159 SSMEHIPCVASISGQTANAYDNLLDSKWMVDAMNGCISYLSNNKFCTDIEKMYAKFDAVF

SLOPH_652_Contig_1894 THGDNQQCMKFINYNIKEIFLKYSKNEWPEEAEMICIWNIR--AYPKPPTKTLGFFETKY

: : : . . * : . *

SLOPH_1121_Contig_782 CDVEQVLKDALYGKLIDRKISK-TIDWEKLMENINGDLSVDDVKVYIYLSERFRNLALHE

SLOPH_1122_Contig_782 KIIERTLKNLKHGKPFKKEIYE-KFNWDKLLNDTGGNFSVTDIKLYIYLCKYLNLISMDE

SLOPH_1080_Contig_7159 LELEQILKNVSQGIPETTTNDQHHLNLSLICIELDNGTTENVLLLYVRINKYLRKLAKIE

SLOPH_652_Contig_1894 RTIEKILIQRKNNIPIVNRYLSGSYNIEEICENEKNGLSELEVTLYIFLCEIIKEIAMLE

:*: * : . : . : : : : :*: : : :. :: *

SLOPH_1121_Contig_782 RGYFSKKGDQDLLDMVMEKFKFFSKYLVVGKDISYKSLLKFCEDFTFKTFNYENYQIFIQ

SLOPH_1122_Contig_782 RLYCLKKTWYDLYMMAVKKADFFSEYFAVGKDASYESVLEFCNDFSLKNFNSEKYQIFLK

SLOPH_1080_Contig_7159 EQLYKYNENKDLRKIAIEKSNFFDNNMVLCENCDYKNILQLYLDFSVRKFNYAKYQVIPK

SLOPH_652_Contig_1894 QEHYLLEGKMDLFEMAKSKRELFTKNIILGNGANYKDYLEFCNDFSFGKCDDTKYQKFEI

. : ** :. .* .:* : : : : .*:. *:: **:. . : :** :

SLOPH_1121_Contig_782 KDYIK-----

SLOPH_1122_Contig_782 STYIMRN---

SLOPH_1080_Contig_7159 NEIHRMVINY

SLOPH_652_Contig_1894 EKMLMYF---

.

***S. lophii* gene family 4:**

SLOPH_962_Contig_5060 MIFAPLLFSTVKSLSQELVTDIASNIANSEKMKTDNPLSNTSLLLMQIQLLVVESATDYM

*SLOPH_2557_Contig_3146 MLFFILFLLSIKSISEEVYSDIYYNMENIEKMESQNPLSNENILLMEIQLLCAQSDYYRY

*:* *:: ::**:*:*: :** *: * ***:::***** .:***:**** .:*

SLOPH_962_Contig_5060 ARKKLVDLRENILALETRDVDVEKIQMTLN--SSNDIITAKTTARIFITTLSTCFDKFKA

SLOPH_2557_Contig_3146 IGDLKKTLLEFIQNLESSKVDTDRIIKYIENLDDPDPIVSRTATKAMIVTLKEAYDKFKA

. * * * **: .**.::* :: .. * *.::*::: :*.**. .:*****

SLOPH_962_Contig_5060 SGEILNDEDKNQLKNLLKEYTEIIEKSGSKMEAPEEKDNVMRLVKLLGEKELEEKMESSF

SLOPH_2557_Contig_3146 TKQELSLEDKRQLINILMDWSIFLKETGQKIYIPKEKDALVAVLQLCDNAELKDKVNDVI

: : *. ***.** *:* ::: :::::*.*: *:*** :: :::* : **::*::. :

SLOPH_962_Contig_5060 K

SLOPH_2557_Contig_3146 E

:
